# Author Correction: A local tumor microenvironment acquired super-enhancer induces an oncogenic driver in colorectal carcinoma

**DOI:** 10.1038/s41467-023-37640-4

**Published:** 2023-04-06

**Authors:** Royce W. Zhou, Jia Xu, Tiphaine C. Martin, Alexis L. Zachem, John He, Sait Ozturk, Deniz Demircioglu, Ankita Bansal, Andrew P. Trotta, Bruno Giotti, Berkley Gryder, Yao Shen, Xuewei Wu, Saul Carcamo, Kaitlyn Bosch, Benjamin Hopkins, Alexander Tsankov, Randolph Steinhagen, Drew R. Jones, John Asara, Jerry E. Chipuk, Rachel Brody, Steven Itzkowitz, Iok In Christine Chio, Dan Hasson, Emily Bernstein, Ramon E. Parsons

**Affiliations:** 1 Department of Oncological Sciences, The Tisch Cancer Institute, Icahn School of Medicine at Mount Sinai, New York, NY 10029 USA; 2 Graduate School of Biomedical Sciences, Icahn School of Medicine at Mount Sinai, New York, NY 10029 USA; 3 Medical Scientist Training Program, Icahn School of Medicine at Mount Sinai, New York, NY 10029 USA; 4 Department of Genetics and Genomic Sciences, Icahn School of Medicine at Mount Sinai, New York, NY 10029 USA; 5 Department of Genetics and Genome Sciences, Case Western Reserve University, Cleveland, OH 44106 USA; 6 Division of Colon and Rectal Surgery, Department of Surgery, Icahn School of Medicine at Mount Sinai, New York, NY 10029 USA; 7 Metabolomics Core Resource Laboratory, NYU Langone Health, New York, NY 10016 USA; 8 Mass Spectrometry Core, Beth Israel Deaconess Medical Center, Boston, MA 02215 USA; 9 Mount Sinai Biorepository, Department of Pathology, Icahn School of Medicine at Mount Sinai, New York, NY 10029 USA; 10 Division of Gastroenterology, Department of Medicine, Icahn School of Medicine at Mount Sinai, New York, NY 10029 USA; 11 Institute for Cancer Genetics, Department of Genetics and Development, Columbia University Medical Center, New York, NY 10032 USA

**Keywords:** Cancer, Gastroenterology

Correction to: *Nature Communications*; 10.1038/s41467-022-33377-8; published online 17 October 2022

The original version of the Supplementary Information omitted Supplementary Tables 1-10. The HTML has been updated to include a corrected version of the Supplementary Information.

## Supplementary information

Updated Supplementary Information

