# Supplementary material for: A local tumor microenvironment acquired super-enhancer induces an oncogenic driver in colorectal carcinoma
Source: Nat Commun. 2022 Oct 17;13:6041. doi: 10.1038/s41467-022-33377-8 (PMC9576746; doi:10.1038/s41467-022-33377-8)
Supplement: Supplementary file 1 — Supplementary Information [file 41467_2022_33377_MOESM1_ESM.pdf]

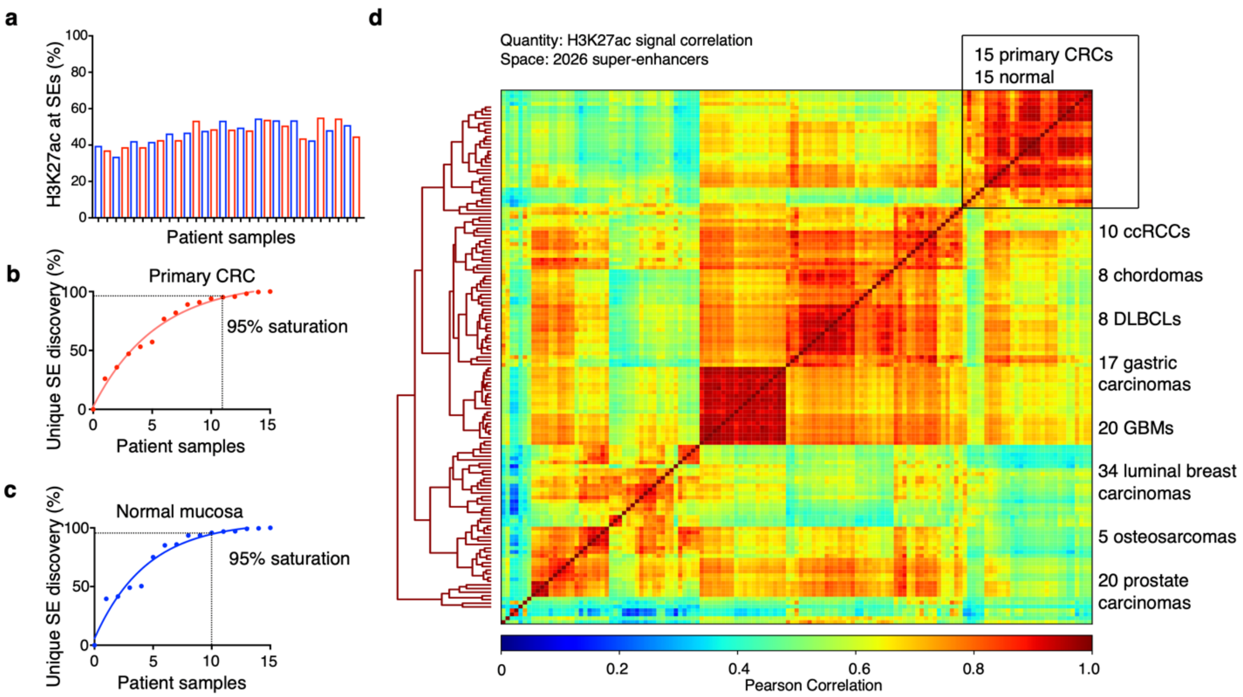

Fig. S1: The super-enhancer landscape in primary CRC and patient-matched adjacent normal colon epithelium. **a** Percentage of H3K27ac signal deposited solely at SEs. Each red or blue bar is an independent primary CRC (*n* = 15 independent samples) or normal colon epithelium sample (*n* = 15 independent samples), respectively. **b, c** Power analyses indicating cumulative percentage saturation of unique super-enhancer discovery with the addition of each patient in cohort. CRC in red, normal mucosa in blue. **d** Unsupervised hierarchical clustering, H3K27ac signal Pearson correlations at 2026 SEs from 30 colorectal samples in our cohort (*n* = 15 independent CRC, *n* = 15 independent normal mucosae) against clear cell renal cell carcinomas (ccRCC, *n* = 10 independent tumors), chordomas (*n* = 8 independent tumors), diffuse large B-cell lymphomas (DLBCL, *n* = 4 independent tumors), gastric carcinomas (*n* = 17 independent tumors), glioblastomas (*n* = 20 independent tumors), luminal breast cancer (*n* = 34 independent tumors),

osteosarcoma (n = 5 independent tumors), and prostate carcinomas (n = 20 independent tumors).

Please see Methods for accessions. Source data are provided as a Source Data file.

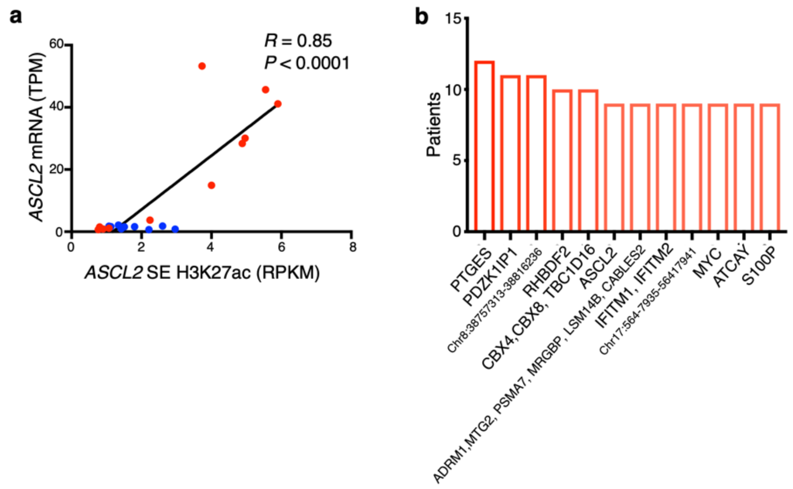

Fig. S2: The super-enhancer landscape in CRC identifies target genes. a Simple linear regression analysis of H3K27ac signal deposition at the *ASCL2* SE and *ASCL2* gene expression using sample matched CRC (n = 11 patients) and normal epithelium (n = 10 patients) Although 15 primary CRCs and 15 normal colons were used for ChIP-seq analyses, sample-matched RNA-seq data was only available for 11 CRCs and 10 normal colons, respectively. All available data was used in this analysis. Please see Methods for details. **b** Histogram of 12 candidate SEs rank-ordered by gained recurrence (number of patients exhibiting SE gain, out of a total of 15). Please see Methods for accessions. Source data are provided as a Source Data file.

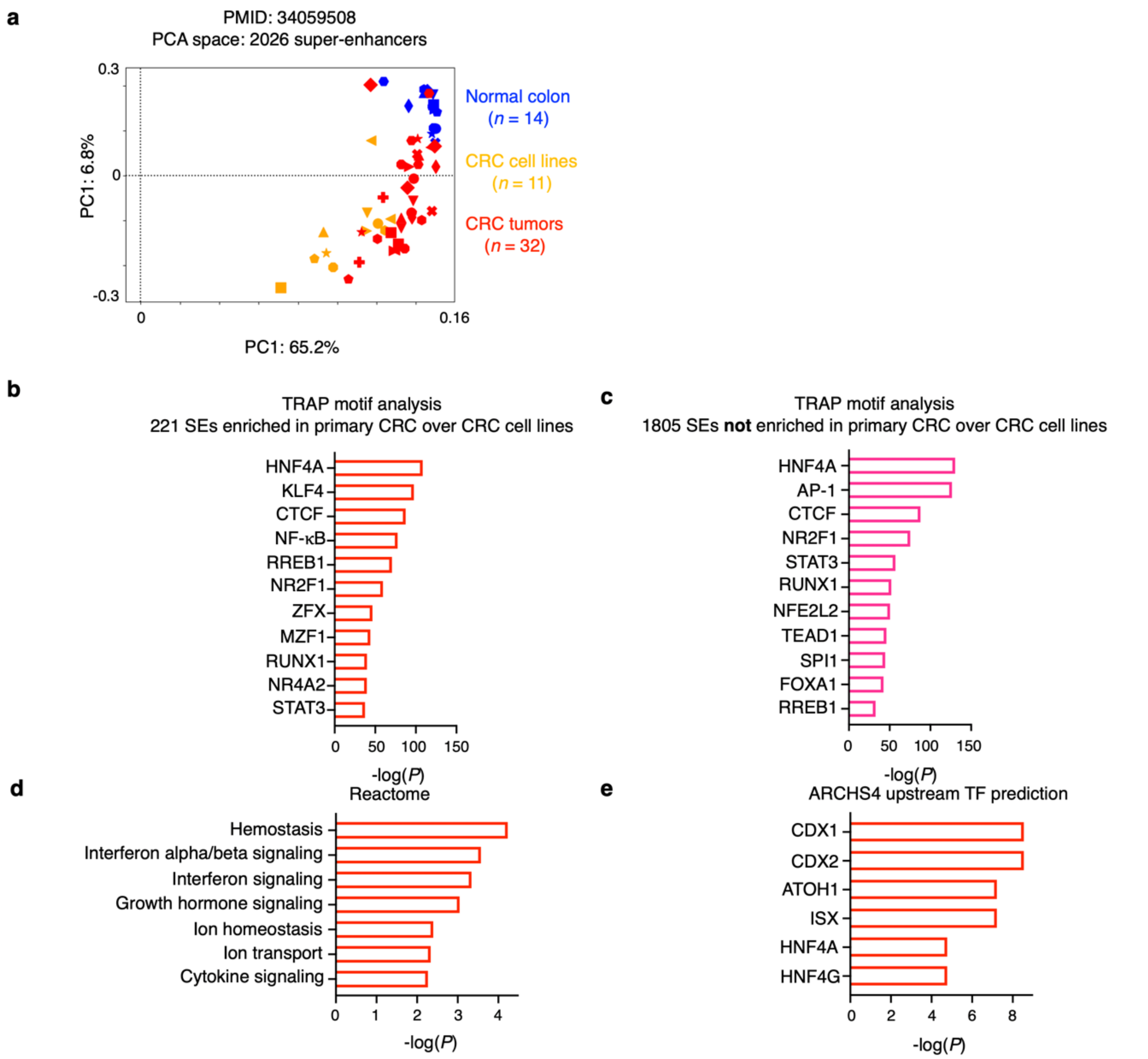

Fig. S3: Characterization of super-enhancers specific to primary CRC under-represented in CRC cell lines. a PCA of H3K27ac signal at 2026 SEs in CRC (*n* = 32 independent tissue samples), CRC cell lines (*n* = 11 independent cell lines), and normal colon mucosa (*n* = 14 independent tissue samples). Data from Orouji et al, please see Methods for accessions. **b** TRAP motif analysis of open chromatin regions within primary CRC-enriched SEs. Open chromatin regions defined by ATAC-seq data from 81 independent primary CRC tumors from TCGA. Please see Methods for accessions. Sequences are compared against all human promoters with a Benjamini-Hochberg correction to generate a *P*-value. **c** TRAP motif analysis of open chromatin regions within primary

CRC non-enriched SEs. Sequences are compared against all human promoters with a Benjamini-Hochberg correction to generate a *P*-value. **d** ENRICHR pathway analysis of a primary CRC-enriched SE gene signature, using the Reactome gene set database. The Fisher's exact test is used for *P*-value. **e** ENRICHR prediction of upstream transcription factors of the primary CRC-enriched SE gene signature using the ARCHS4 tool. The Fisher's exact test is used for *P*-value. Source data are provided as a Source Data file.

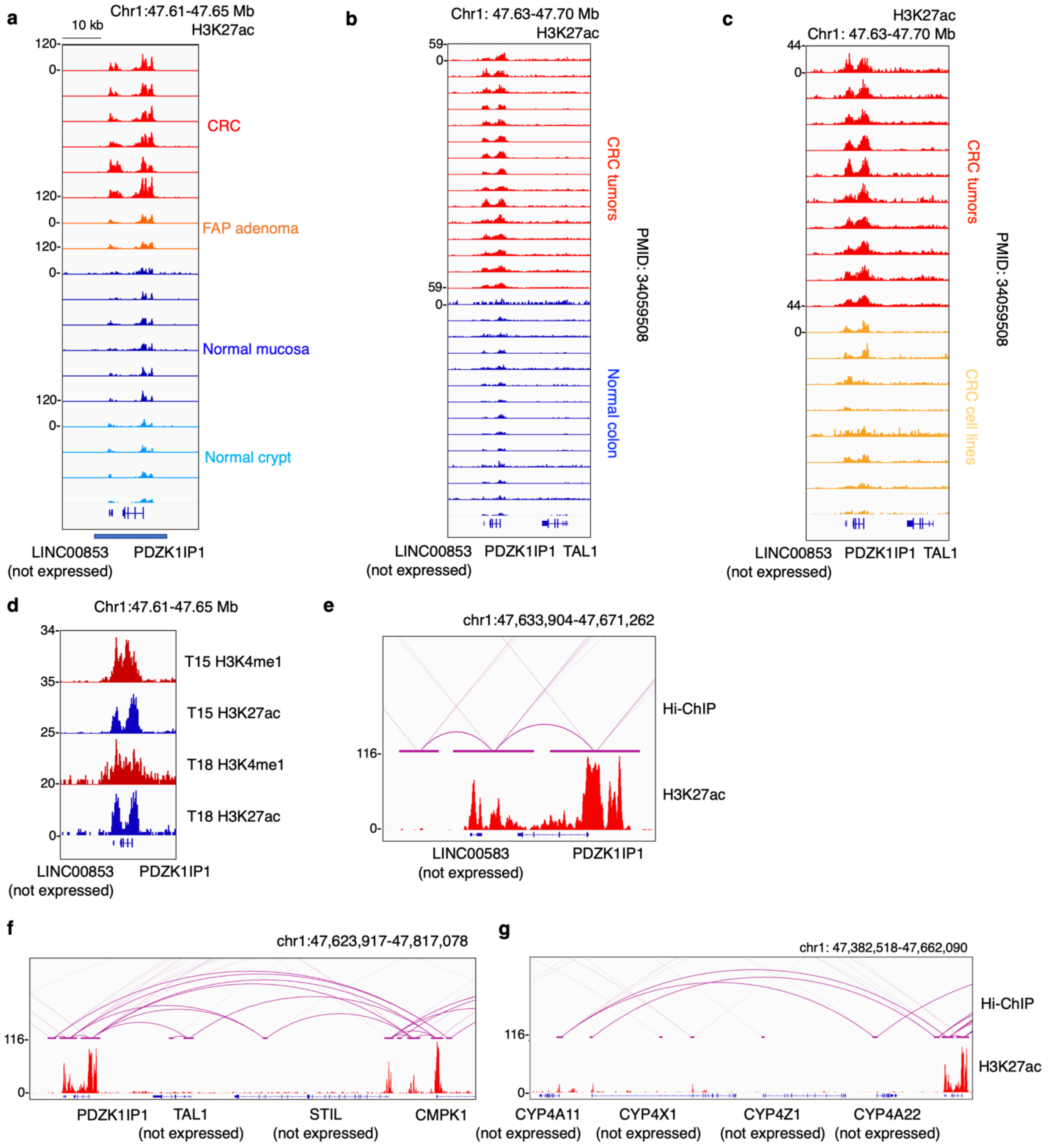
a
Chr1:47.61-47.65 Mb
10 kb
H3K27ac
120-
0-
CRC
FAP adenoma
Normal mucosa
Normal crypt
LINC00853
(not expressed)
PDZK1IP1
b
Chr1: 47.63-47.70 Mb
H3K27ac
59-
0-
CRC tumors
PMID: 34059508
Normal colon
LINC00853
(not expressed)
PDZK1IP1
TAL1
c
H3K27ac
Chr1: 47.63-47.70 Mb
44-
0-
CRC tumors
PMID: 34059508
CRC cell lines
LINC00853
(not expressed)
PDZK1IP1
TAL1
d
Chr1:47.61-47.65 Mb
34-
35-
25-
20-
0-
T15 H3K4me1
T15 H3K27ac
T18 H3K4me1
T18 H3K27ac
LINC00853
(not expressed)
PDZK1IP1
e
chr1:47,633,904-47,671,262
Hi-ChIP
116-
H3K27ac
0-
LINC00583
(not expressed)
PDZK1IP1
f
chr1:47,623,917-47,817,078
116-
0-
PDZK1IP1
TAL1
(not expressed)
STIL
(not expressed)
CMPK1
g
chr1: 47,382,518-47,662,090
Hi-ChIP
116-
H3K27ac
0-
CYP4A11
(not expressed)
CYP4X1
(not expressed)
CYP4Z1
(not expressed)
CYP4A22
(not expressed)

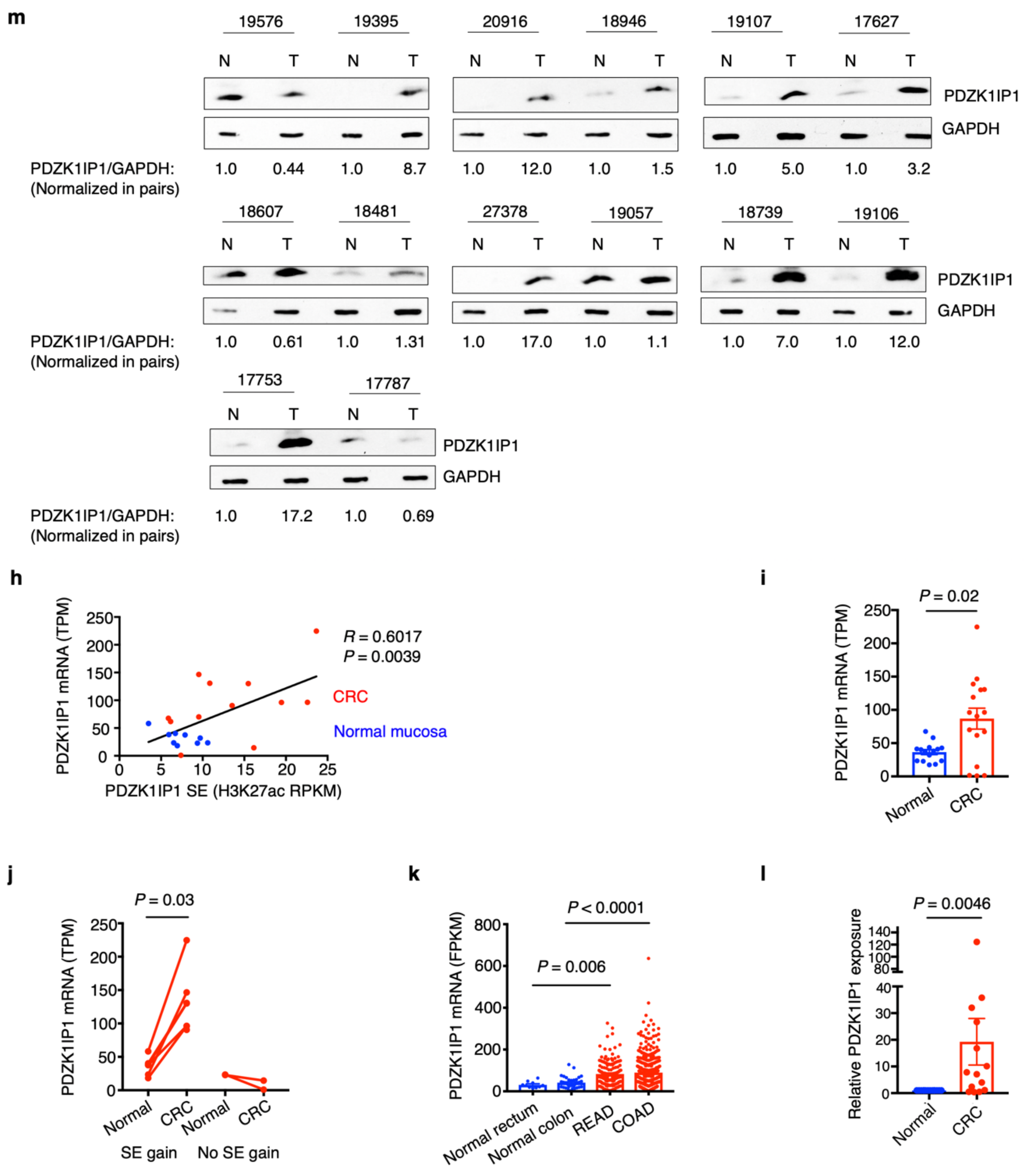

Fig. S4: ***PDZK1IP1*** **and its super-enhancer are up-regulated in CRC. a** H3K27ac ChIP-seq track at the *PDZK1IP1* SE (underlined) in 6 representative independent primary CRCs, 2 FAP adenomas, 6 representative independent normal colon epithelium samples, and 4 normal colon crypts. **b** H3K27ac ChIP-seq track at the *PDZK1IP1* SE in 15 representative independent primary CRCs and normal colon epithelium samples. Data from Orouji et al. **c** H3K27ac ChIP-seq track at

the *PDZK1IP1* SE in 10 representative independent primary CRCs and 8 representative CRC cell lines. Data from Orouji et al. **d-g** H3K27ac ChIP-seq and Hi-ChIP integration showing genome interactions at the *PDZK1IP1* super-enhancer. Y-axes for all ChIP-seq tracks are scaled to the same range. Please see Methods for accessions. All data presented as mean ± s.e.m and significance determined using two-sided Student's *t*-test. **h** Simple linear regression analysis of *PDZK1IP1* SE H3K27ac signal and *PDZK1IP1* mRNA expression using sample-matched sequencing data. For CRC n = 11 independent patient samples, for normal mucosa n = 10. Although 15 primary CRCs and 15 normal colons were used for ChIP-seq analyses, sample-matched RNA-seq data was only available for 11 CRCs and 10 normal colons, respectively. All available data was used in this analysis. **i, j** *PDZK1IP1* mRNA expression by RNA-seq in our cohort of patients. For CRC n = 16 independent tumors; for normal mucosa n = 15 independent tissue samples. Two-sided Student's *t*-test. **k** *PDZK1IP1* mRNA expression by RNA-seq between normal colon (n = 42 independent cases), normal rectum (n = 10 independent cases) rectal adenocarcinoma (READ, n = 166 independent cases), and colon adenocarcinoma (COAD, n = 475 independent cases) from TCGA. Two-sided Student's *t*-test. **l, m** *PDZK1IP1* protein levels by immunoblot (fold change, CRC over normal for each pair). n = 14 de-identified patients, each with a matched tumor normal pair. T – tumor, N – matched normal. Five-digit de-identified patient number. Two-sided Student's *t*-test. **j** H3K4me1 and H3K27ac ChIP-seq tracks at the PDZK1IP1 super-enhancer in two CRC samples, which met ROSE criteria for super-enhancer calling using either as input. Source data are provided as a Source Data file.

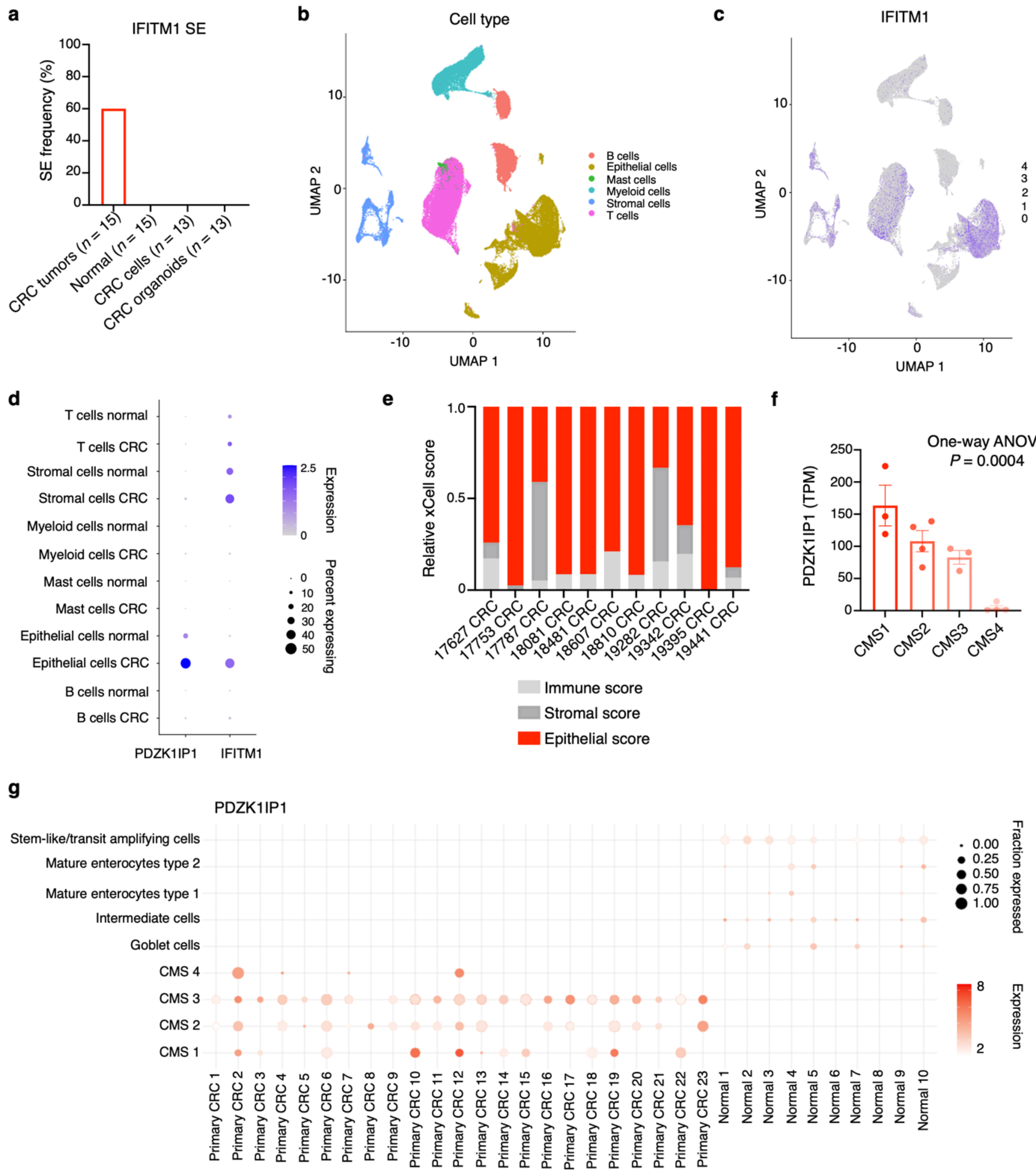

Fig. S5: Characterization of CRC-enriched SEs at *IFITM1* and *PDZK1IP1*. a Frequency of samples meeting ROSE criteria for SE calling at the *IFITM1* locus. n = 15 independent CRC tumors, n = 15 independent patient-matched normal colon mucosae, n = 13 independent CRC cell lines, n = 9 independent 3D CRC organoids. **b, c** UMAP of single cells from 23 independent

primary CRC tumors and 10 independent adjacent patient-matched normal colons merged into a single plot, annotated with cell type and *IFITM1* expression. Please see Methods for accessions. **d** Dot plot of *PDZK1IP1* or *IFITM1* expression in single cells from primary CRC or adjacent normal colon epithelium. **e** xCell de-convolution of bulk tumor RNA-seq into putative epithelial, immune, and stromal compositions. Out of the 15 primary CRCs for which H3K27ac ChIP-seq data is available, only 11 have sample-matched bulk tumor RNA-seq data. All available data was used in this analysis. **f** RNA-seq expression of *PDZK1IP1* by CMS. For CMS1 (n = 3 patients), for CMS2-4 (n = 4 patients per group). Data presented as mean ± s.e.m. **g** Dot plot of *PDZK1IP1* expression in single cells of epithelial origin, from either primary CRC (n = 23 independent cases) or patient-matched adjacent normal epithelium (n = 10 independent cases). CMS refers to the consensus molecular subtypes of CRC as previously assigned. Please see Methods for accessions. Source data are provided as a Source Data file.

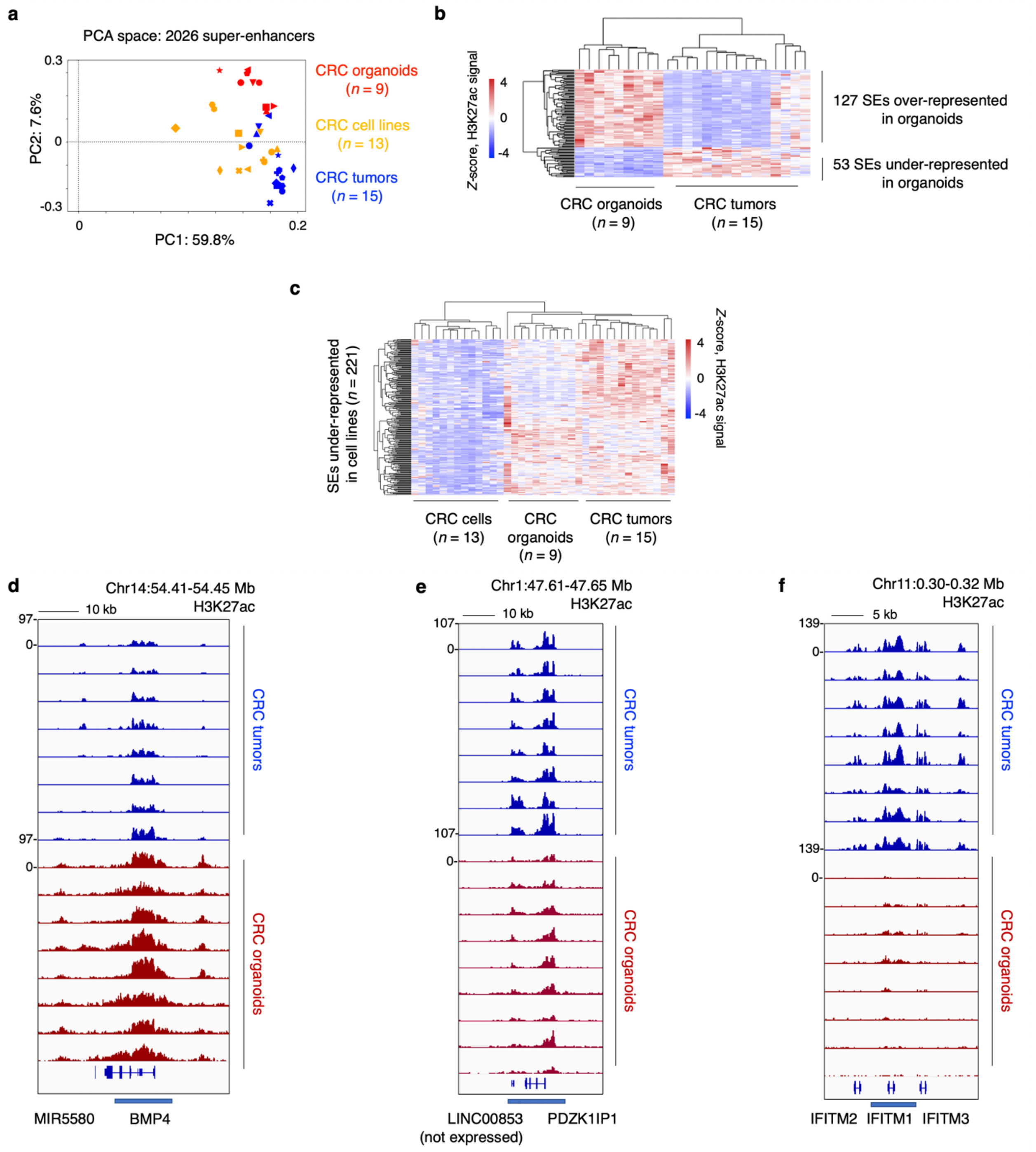

Fig. S6: Super-enhancers specific to primary CRC specimens and not recapitulated in 3D CRC organoids. **a** PCA of H3K27ac signal at 2026 SEs in CRC (*n* = 15 independent tissue samples), CRC 3D organoids (derived from *n* = 9 independent patients), and CRC cell lines (*n* = 13 independent lines). Please see Methods for accessions. **b** Heatmap of H3K27ac signal (*Z*-score)

between CRC tumors and CRC 3D organoids with unsupervised hierarchical clustering at 180 differentially expressed SEs (absolute mean $\log_2$ fold change >1, $P < 0.01$). **c** Incorporation of 3D CRC organoids into the heatmap of 221 SEs down-regulated in 2D CRC cell lines from Figure 2. **d** H3K27ac ChIP-seq track of the SE at *BMP4* (underlined) in $n = 8$ representative primary CRCs and $n = 8$ representative 3D CRC organoid cultures. Y-axes of all ChIP-seq tracks are scaled to the same range [0-97]. **e** H3K27ac ChIP-seq track of the SE at *PDZK1IP1* (underlined) in $n = 8$ representative primary CRCs and $n = 8$ representative 3D CRC organoid cultures. Y-axes of all ChIP-seq tracks are scaled to the same range [0-107]. **f** H3K27ac ChIP-seq track of the SE at *IFITM1* (underlined) in $n = 8$ representative primary CRCs and $n = 8$ representative 3D CRC organoid cultures. Y-axes of all ChIP-seq tracks are scaled to the same range [0-139].

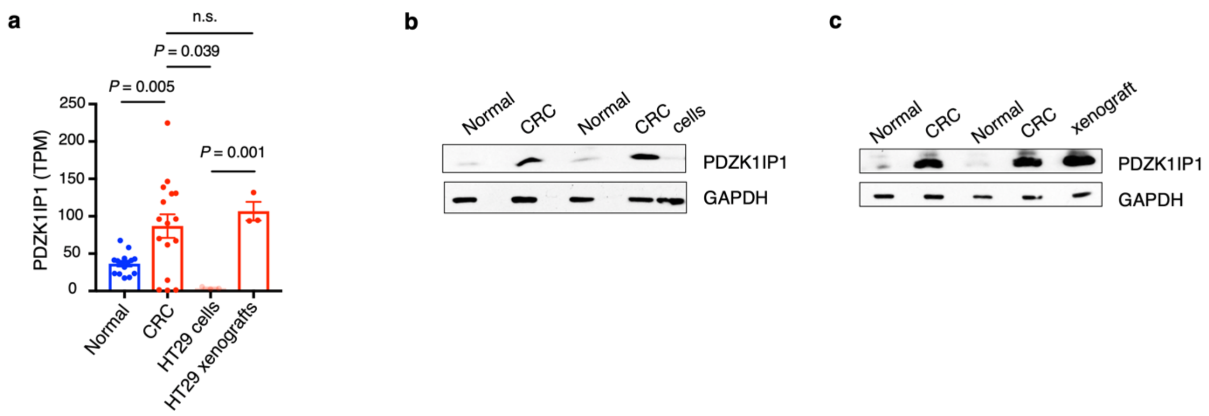

Fig. S7: *PDZK1IP1* expression levels in HT29 xenografts recapitulate those in primary CRC. **a** *PDZK1IP1* expression by RNA-seq, HT29 cells (*n* = 3 biological replicates), HT29 xenografts (*n* = 3 independent tumors), normal mucosa (*n* = 15 independent tissue samples), and primary CRC (*n* = 16) independent tumors Data presented as mean ± s.e.m. Significance was determined using two-sided Student's *t*-test. **b, c** *PDZK1IP1* expression by immunoblot across four independent patient-matched CRC-normal colon pairs, versus HT29 cells or HT29 xenograft tumors. Source data are provided as a Source Data file.

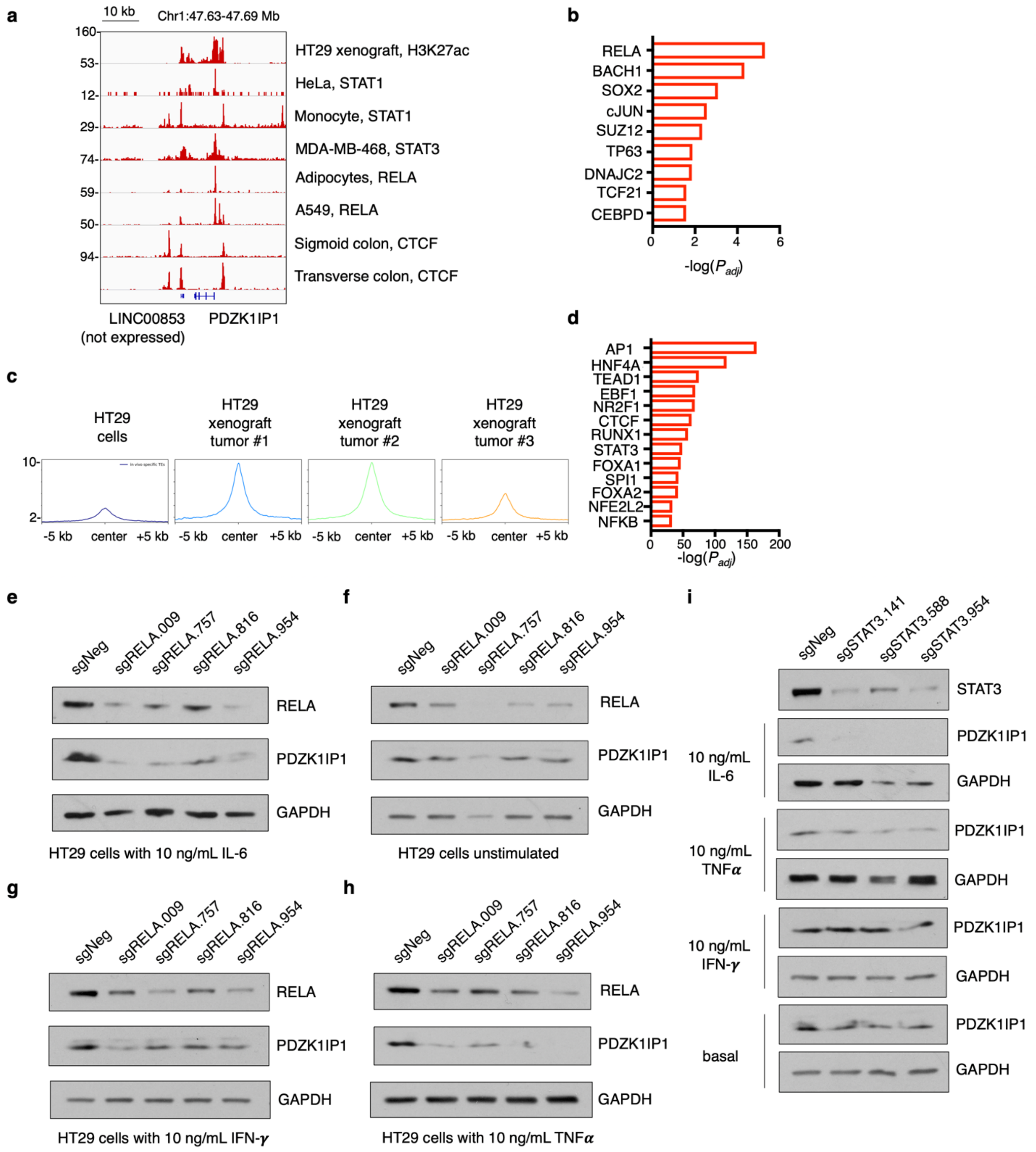

Fig. S8: *PDZK1IP1* is regulated by inflammation. a H3K27ac, STAT1, STAT3, RELA, and CTCF ChIP-seq tracks at the *PDZK1IP1* SE. See Methods for accessions. The y-axes bottom limit for all ChIP-seq tracks is 0. **b** ChEA TF predictions upstream of up-regulated genes (log_2 fold change >2) from RNA-seq data in HT29 xenograft tumors over HT29 parental cells in culture. **c**

Metagene of up-regulated H3K27ac enhancer peaks (mean $\log_2$ fold change > 1) in HT29 xenograft tumors (n = 3 independent tumors) over HT29 parental cells maintained in culture. The Fisher's exact test is used for P-value. **d** TRAP TF motif analysis output for H3K27ac enhancers shown in **c**. Sequences are compared against all human promoters with a Benjamini-Hochberg correction to generate a P-value. **e-h** Immunoblot of PDZK1IP1 levels in sgRELA or sgNeg HT29 cells treated with cytokines. **i** Immunoblot of PDZK1IP1 levels in sgSTAT3 or sgNeg HT29 cells treated with cytokines. Source data are provided as a Source Data file.

a

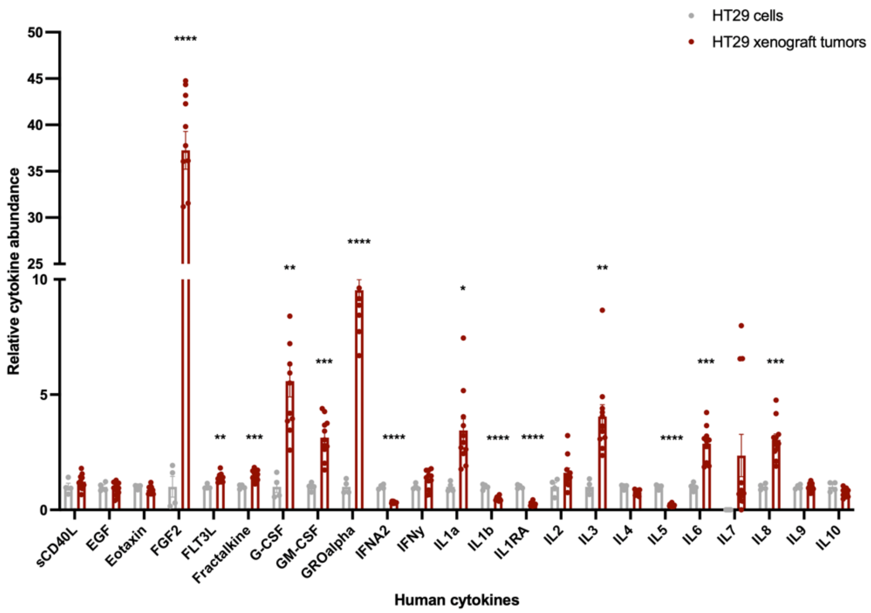
HT29 cells
HT29 xenograft tumors
Relative cytokine abundance
50
45
40
35
30
25
10
5
0
sCD40L
EGF
Eotaxin
FGF2
FLT3L
Fractalkine
G-CSF
GM-CSF
GROalpha
IFNA2
IFNy
IL1a
IL1b
IL1RA
IL2
IL3
IL4
IL5
IL6
IL7
IL8
IL9
IL10
Human cytokines

b

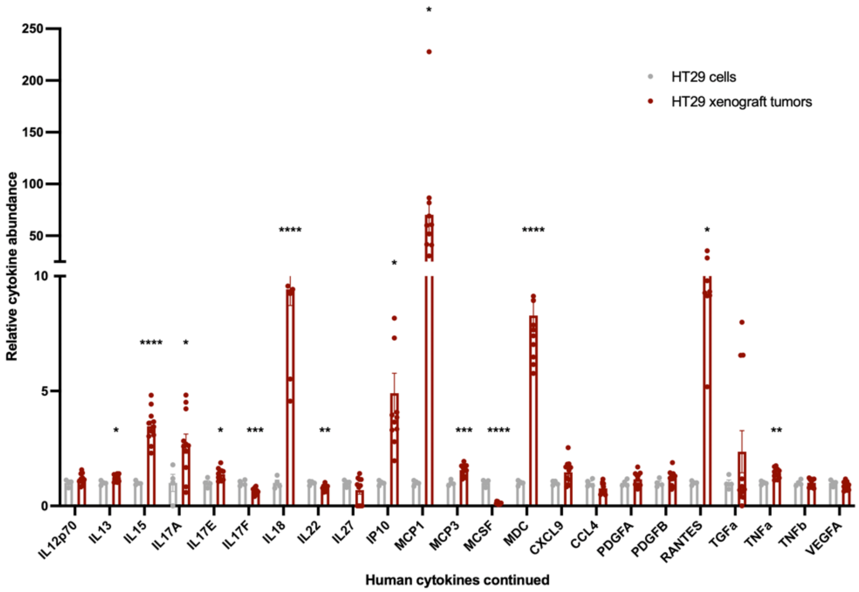
HT29 cells
HT29 xenograft tumors
Relative cytokine abundance
250
200
150
100
50
10
5
0
IL12p70
IL13
IL15
IL17A
IL17E
IL17F
IL18
IL22
IL27
IP10
MCP1
MCP3
MCSF
MDC
CXCL9
CCL4
PDGFA
PDGFB
RANTES
TGFa
TNFa
TNFb
VEGFA
Human cytokines continued

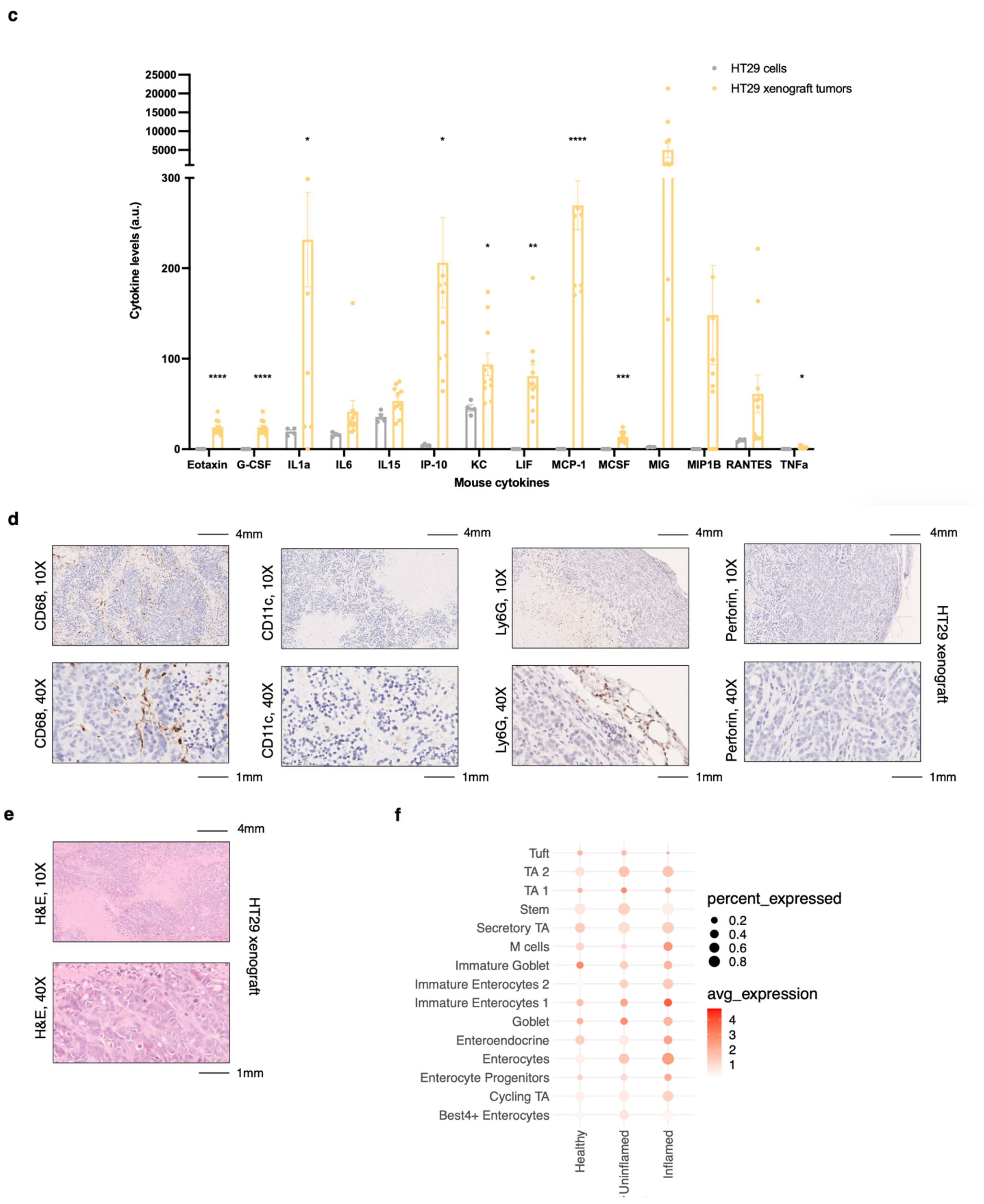

Fig. S9: Xenograft tumors exhibit increased pro-inflammatory cytokines, chemokines, and growth factors upstream of *PDZK1IP1*. a-b Luminex-based human and **c** mouse cytokine

profiling of HT29 xenograft tumor lysates (n = 11 biological replicates) and HT29 parental cells (n = 4 biological replicates) in culture (performed by EveTechnologies). Data presented as mean ± s.e.m. Significance was determined using two-sided Student's t-test. * $P < 0.05$, ** $P < 0.01$, *** $P < 0.001$, **** $P < 0.0001$. Human IL-7 and mouse eotaxin, G-CSF, LIF, MCP-1, MCSF, MIP1B, and TNFa were undetectable in HT29 cells. **d, e** IHC and H&E staining of immune cell markers in HT29 xenograft tumors. **f** Dot plot of *PDZK1IP1* expression in single cells of epithelial origin from healthy patients without colitis, uninflamed colon of patients with colitis, and inflamed colon of patients with colitis. Source data are provided as a Source Data file.

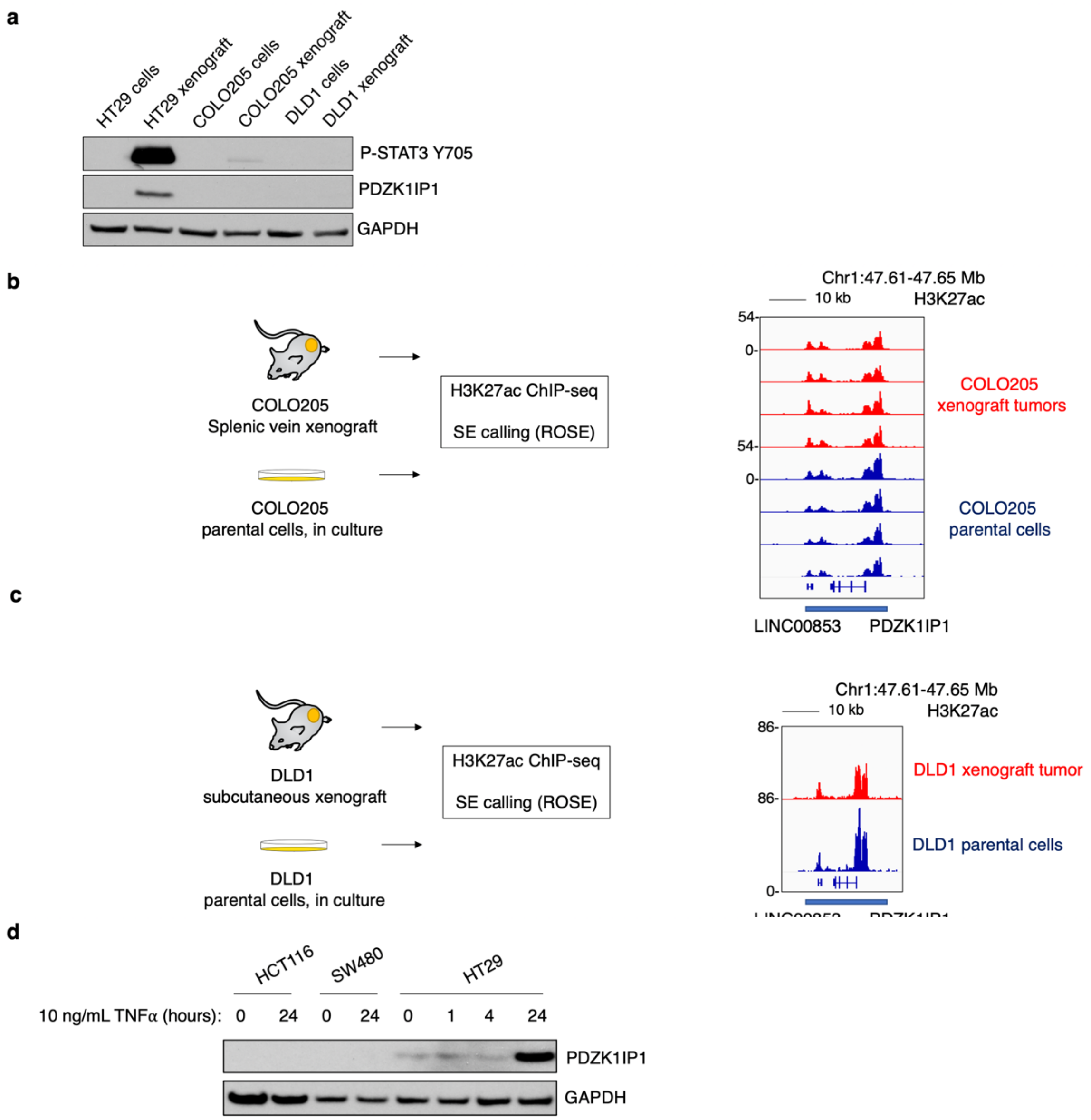

Fig. S10: Cell-specific super-enhancer reprogramming in response to microenvironment. a *PDZK1IP1* protein levels correlated with phosphorylation of STAT3 at the Y705 residue in parental cells maintained in culture or subcutaneous xenografts in nude mice. **b** Experiment overview. A xenograft model of COLO205 liver metastases in nude mice via splenic vein injection. H3K27ac ChIP-seq of COLO205 xenograft tumors in nude mice (n = 4 independent tumors) and COLO205 parental cells maintained in culture (n = 4 biological replicates). Y-axes of all ChIP-seq tracks are scaled to the same range [0-54]. **c** Experiment overview. A subcutaneous xenograft

tumor model of DLD1 cells in nude mice. H3K27ac ChIP-seq of DLD1 xenograft tumors or DLD1 parental cells maintained in culture. Y-axes of all ChIP-seq tracks are scaled to the same range [0-86]. **d** *PDZK1IP1* protein levels by immunoblot with or without TNFα stimulation in HCT116, SW480, or HT29 cells. Panels **b, c** contain medical illustrations from SMART Servier Medical Art, reproduced with permission, licensed under a Creative Commons Attribution 3.0 unported license.

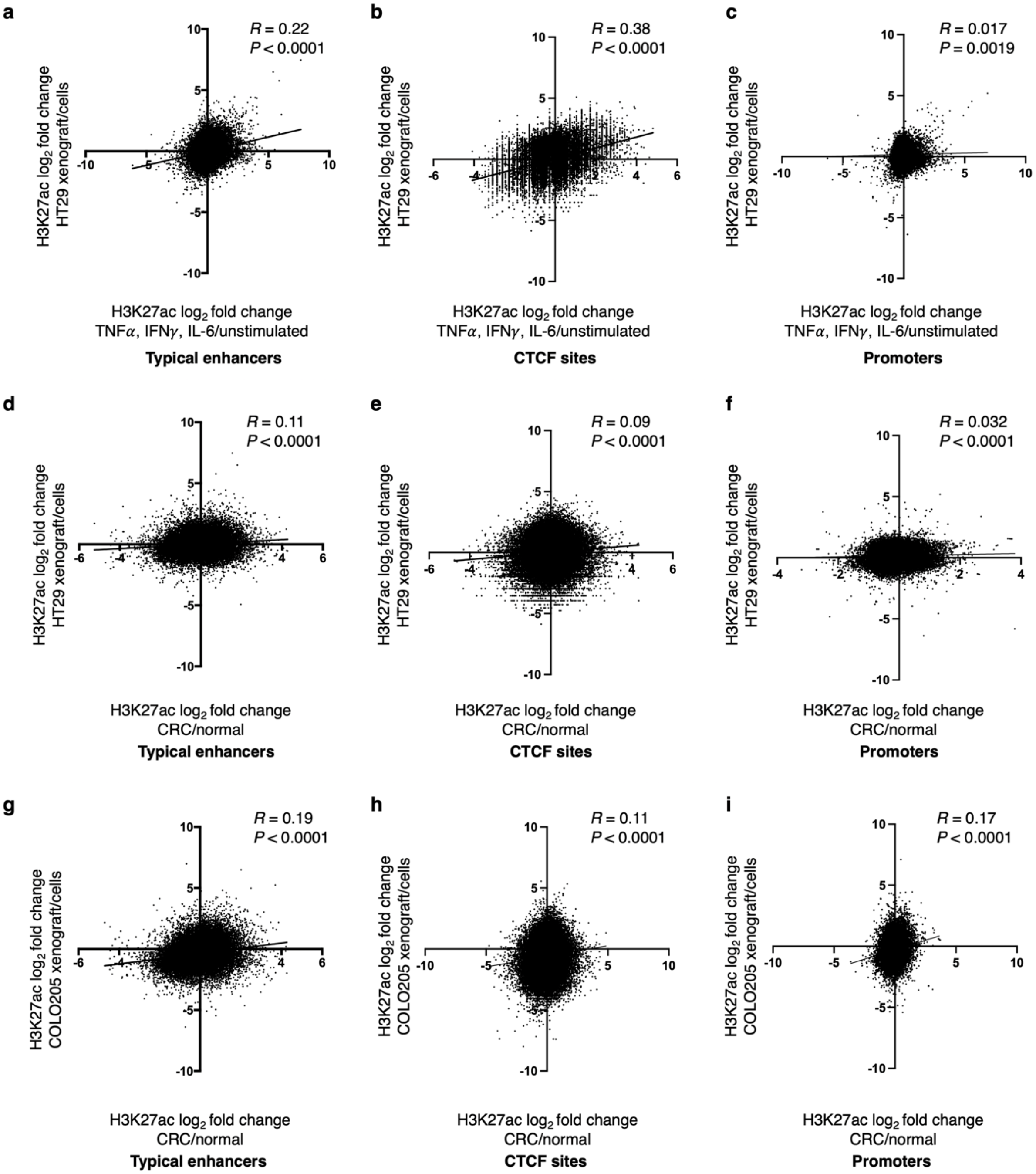

Figure S11. The tumor microenvironment recapitulates enhancer reprogramming in cancer.

a-c Simple linear regression analysis between $\log_2$ fold change H3K27ac signal in HT29 nude mice subcutaneous xenografts (n = 3 independent tumors) over parental cells *in vitro*, and TNFα, IFNγ, IL-6 stimulated cells (10 ng/mL, 16 hours) over unstimulated cells. Each dot represents a unique typical enhancer, CTCF site, or promoter. **d-f** Simple linear regression analysis between $\log_2$ fold change H3K27ac signal in HT29 xenografts/parental cells and CRC/normal mucosa. Each dot represents a unique typical enhancer, CTCF site, or promoter. **g-i** Simple linear regression analysis between $\log_2$ fold change H3K27ac signal in COLO205 xenografts/parental cells and CRC/normal mucosa. Each dot represents a unique typical enhancer. Source data are provided as a Source Data file.

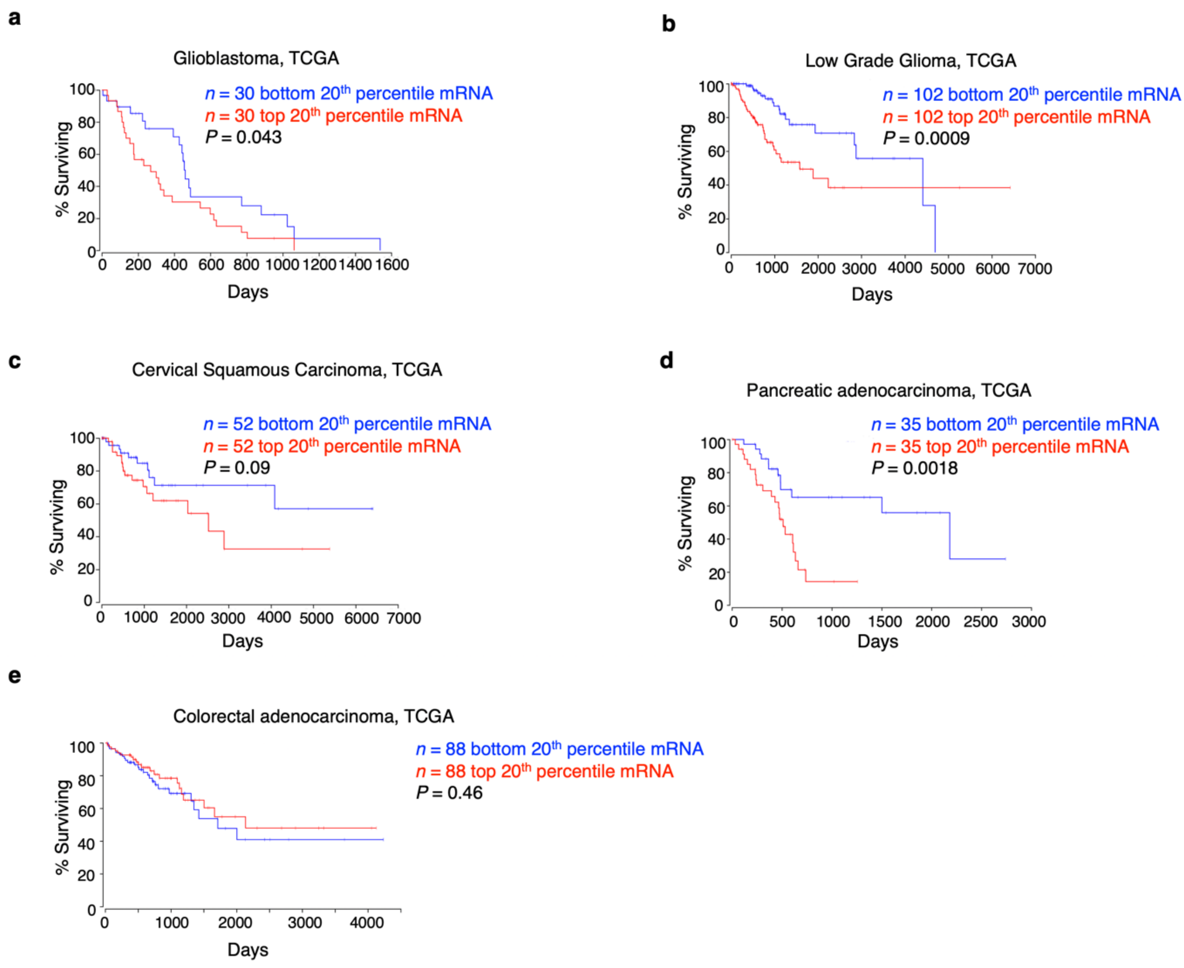

Fig. S12: High *PDZK1IP1* expression negatively prognosticates patient survival in multiple cancers but not CRC. a-e Kaplan-Meier analysis and survival curves of *PDZK1IP1* high (top 20% mRNA, in red) and low (bottom 20% mRNA, in blue) cancer cases. Patient survival and gene mRNA expression data from TCGA, visualized using OncoLnc.

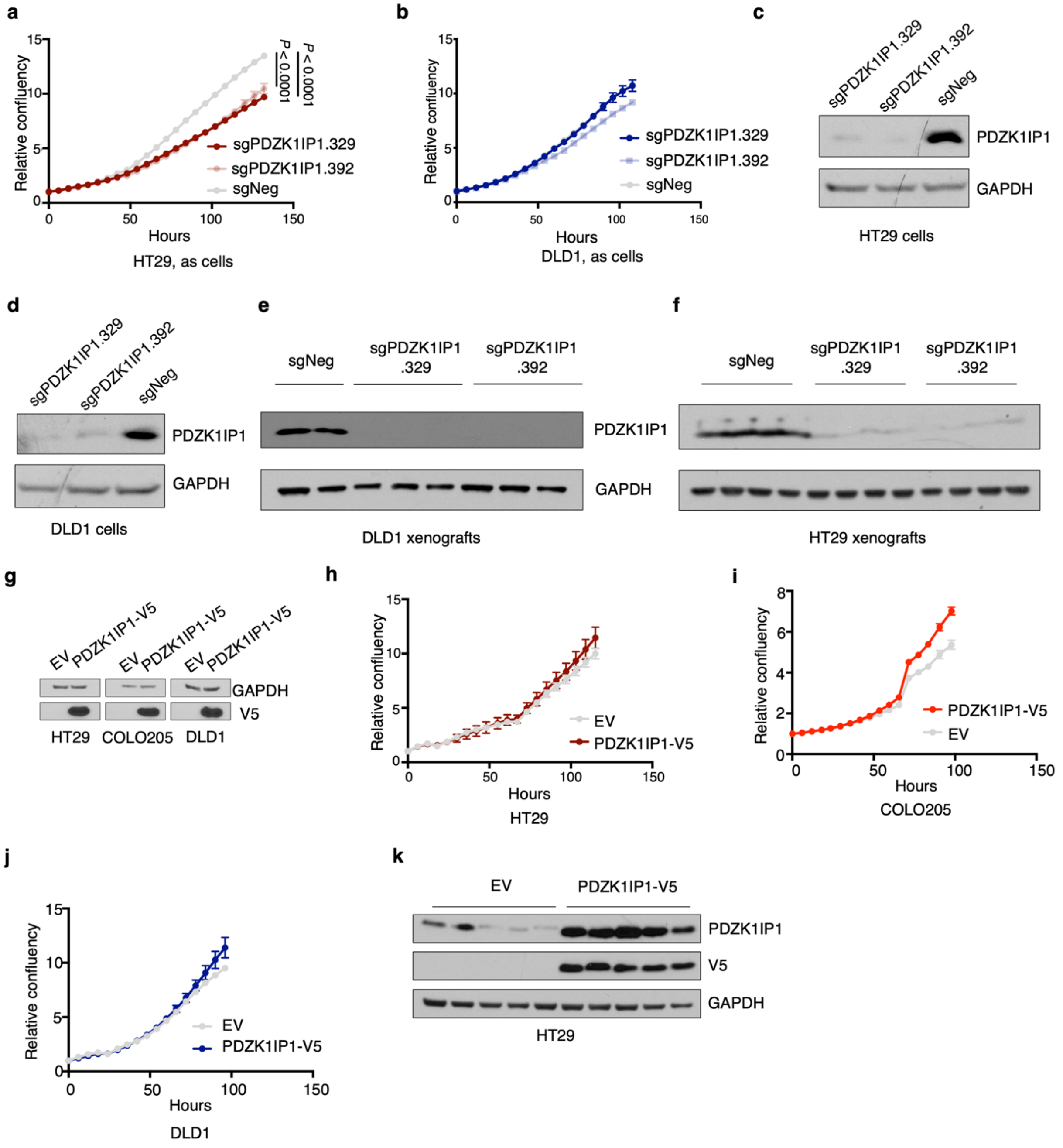

Fig. S13: *PDZK1IP1* does not affect CRC proliferation in culture. a, b Incucyte 2D growth curves of sgNeg or sgPDZK1IP1 cells (n = 6 biological replicates per group). Data presented as

mean ± s.e.m. Significance was determined using two-sided Student's *t*-test. **c, d** *PDZK1IP1* protein levels by immunoblot in HT29 and DLD1 cells between sgPDZK1IP1 and sgNeg. Data presented as mean ± s.e.m. **e, f** *PDZK1IP1* protein levels by immunoblot in HT29 and DLD1 subcutaneous xenograft tumors in nude mice between sgPDZK1IP1 and sgNeg. **g** Re-introducing exogenous *PDZK1IP1* expression *in vitro*. **h-j** Incucyte 2D growth curves of EV or PDZK1IP1-V5 expressing cells in HT29, COLO205, or DLD1 (n = 6 biological replicates per group). Data presented as mean ± s.e.m. **k** *PDZK1IP1* protein levels by immunoblot in HT29 subcutaneous xenograft tumors in nude mice between EV and PDZK1IP1-V5 expressing cells. Source data are provided as a Source Data file.

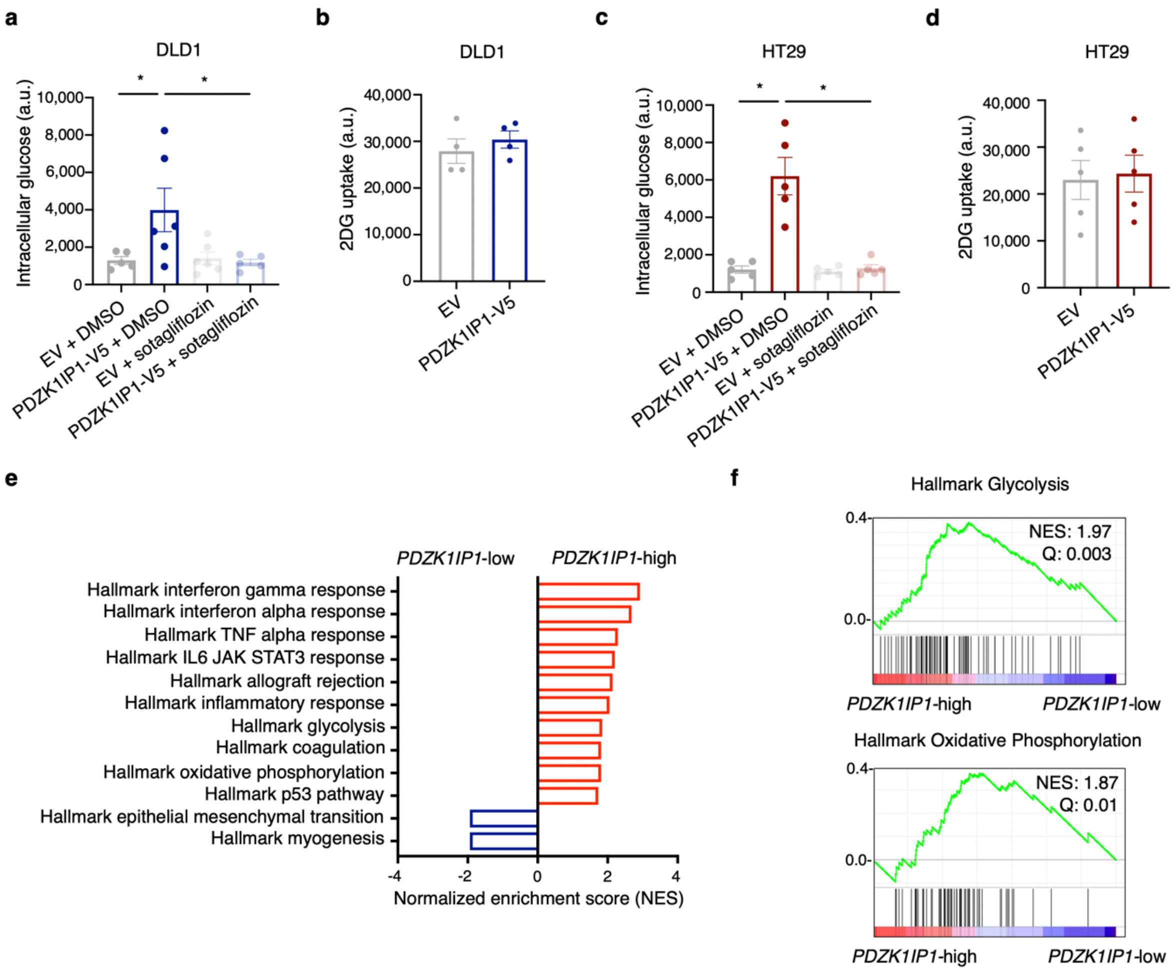

Fig. S14: *PDZK1IP1* increases glucose uptake and is linked to cancer metabolism. a, c Cellular glucose levels by fluorometric assay in cell lysates in the presence or absence of 25 μM sotagliflozin. For HT29 (*n* = 5 biological replicates for per group) and DLD1 (*n* = 5 biological replicates for EV + DMSO and PDZK1IP1-v5 + sotagliflozin; *n* = 6 for PDZK1IP1-V5 + DMSO and EV + sotagliflozin). Data presented as mean ± s.e.m. Significance was determined using two-sided Student's *t*-test. * $P < 0.05$ **b, d** 2DG uptake by luminescent assay in cell lysates. For HT29 (*n* = 5 biological replicates per group) and DLD1 (*n* = 4 biological replicates per group). Data

presented as mean ± s.e.m. **e, f** Hallmarks of Cancer GSEA of RNA-seq expression data from *PDZK1IP1*-high (top 50% mRNA expression) versus *PDZK1IP1*-low (bottom 50% mRNA expression) primary CRCs from TCGA (n = 342 independent tumors). Source data are provided as a Source Data file.

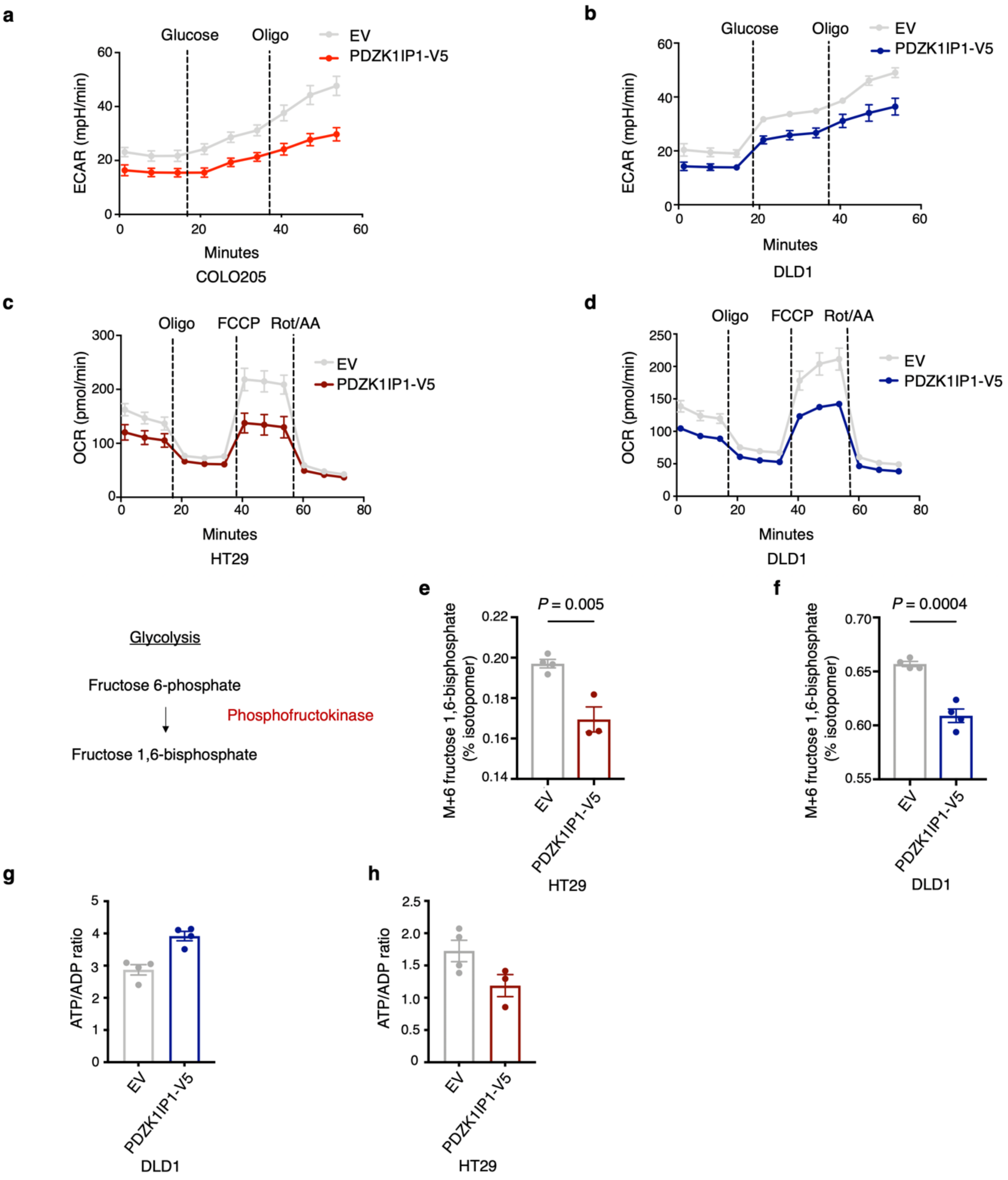

Fig. S15: *PDZK1IP1* decreases glycolytic and mitochondrial respiration rate. a, b Seahorse glycolytic stress test. ECAR – extracellular acidification rate, a readout of glycolysis rate ($n = 6$

biological replicates per group). Data presented as mean ± s.e.m. **c, d** Seahorse mitochondrial stress test. OCR – oxygen consumption rate, a readout of oxidative respiration rate (*n* = 6 biological replicates per group). Oligo – oligomycin, an ATP synthase inhibitor which decreases OCR and increase ECAR. FCCP – trifluoromethoxy carbonylcyanide phenylhydrazone, an inner mitochondrial membrane gradient decoupler that increases OCR. Rot/AA – Rotenone and antimycin A, complex I and complex III inhibitors, respectively, which decrease OCR. Data presented as mean ± s.e.m. **e, f** Relative peak area of uniformly labeled fructose 1,6-bisphopshate, reported as % of M+6 isotopomer of total labeled and unlabeled metabolite, from U-C^{13} glucose tracing (*n* = 4 biological replicates per group) as a readout of phosphofructokinase enzyme live-cell activity. Data presented as mean ± s.e.m. Significance was determined using two-sided Student's *t*-test. **g, h** ATP/ADP ratio from total polar metabolite peak area in HT29 and DLD1 cells (*n* = 4 biological replicates per group). Data presented as mean ± s.e.m. Significance was determined using two-sided Student's *t*-test. Source data are provided as a Source Data file.

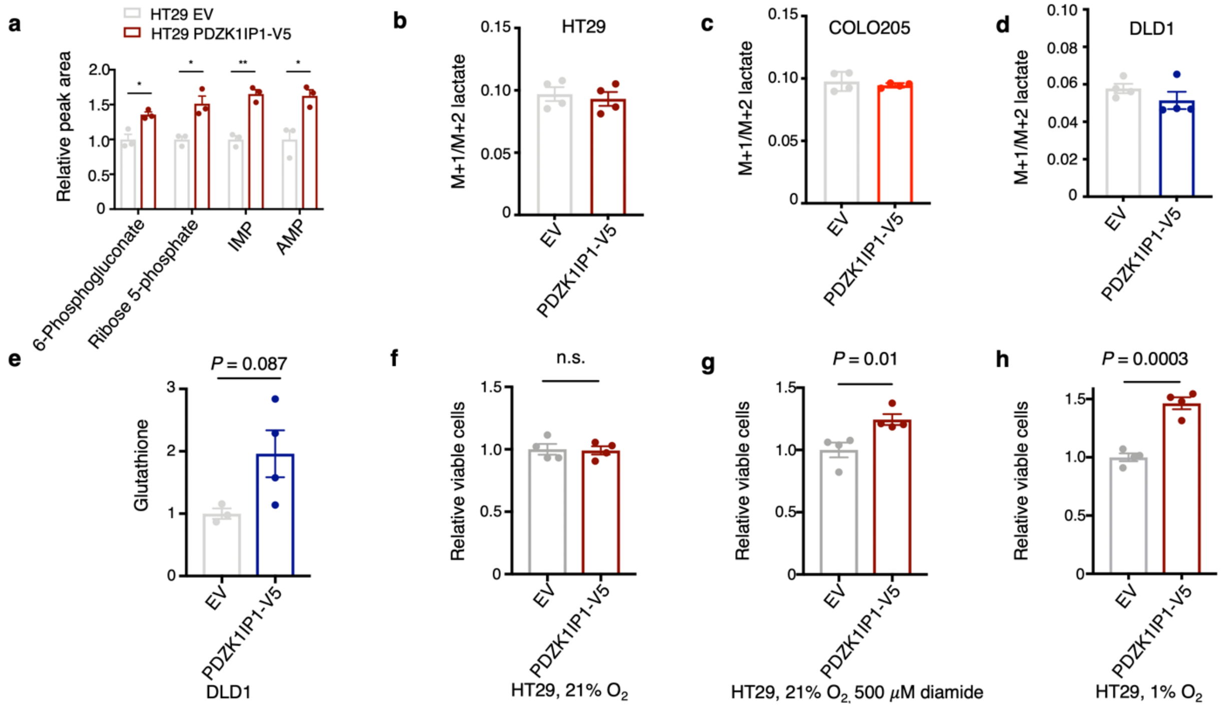

Fig. S16: *PDZK1IP1* maintains redox homeostasis. a Steady state levels (total peak are) of PPP intermediates and downstream metabolites (n = 3 per group). Data presented as mean ± s.e.m. Significance was determined using Student's t-test. * $P < 0.05$, ** $P < 0.01$. **b-d** Ratio of M+1/M+2 lactate levels by total peak area, from 5 mM 1,2C^{13}-glucose labeling for 4 hours (n = 4 for all groups). Data presented as mean ± s.e.m. **e** Steady state levels of total glutathione peak area (n = 4 per group). Data presented as mean ± s.e.m. Significance was determined using Student's t-test. **f-h** Cell viability by colorimetric MTT assay under normoxia, hypoxia for 48 hours, or with 30 minutes of diamide treatment (n = 4 per group). Data presented as mean ± s.e.m. Significance was determined using Student's t-test. Source data are provided as a Source Data file.

Supplementary Table 1: 2026 super-enhancers and putative gene associations by H3K27ac ChIP-seq signal and RNA-seq mRNA expression correlation.

#'chr'	'start'	'end'	Genes associated with super-enhancer by H3K27ac and mRNA correlation
chr1	839207	879441	CPSF3L
chr1	931032	942910	HES4
chr1	958033	1016775	
chr1	1056306	1073787	RNF223
chr1	1076920	1083409	RNF223
chr1	1092834	1107947	RNF223
chr1	1278680	1285456	CPSF3L

chr1	1364974	1379781	ACAP3, C1orf222, SCNN1D, MXRA8, VWA1, TNFRSF4, SLC35E2B, MIB2, TMEM240, SLC35E2, MMP23A, ANKRD65
chr1	1530021	1565186	
chr1	1680282	1729724	PRKCZ
chr1	1820906	1828473	PRKCZ
chr1	1954672	1985568	PLCH2
chr1	2063722	2092933	C1orf222, SLC35E2B, SLC35E2
chr1	2157893	2167812	PLCH2
chr1	2346593	2438208	PLCH2
chr1	2473058	2481551	PLCH2
chr1	2485426	2494310	PLCH2
chr1	3228780	3277512	MEGF6
chr1	3356135	3431583	LRRC47
chr1	6326718	6362599	ESPN
chr1	6379836	6456346	
chr1	6506270	6571895	ESPN
chr1	6639352	6664607	RPL22
chr1	8120867	8182464	
chr1	8207520	8285533	PARK7
chr1	8456022	8488406	
chr1	8931738	8960596	CA6
chr1	9221417	9243600	TMEM201
chr1	9326975	9508779	SPSB1
chr1	9858949	9909954	NMNAT1
chr1	10726087	10897857	MASP2
chr1	11766429	11867807	MAD2L2
chr1	12183318	12269678	MIIP
chr1	12651250	12690803	PRAMEF1, TNFRSF8

chr1	15455486	15557536	CASP9
chr1	15634011	15698833	CASP9
chr1	15735516	15760330	CASP9
chr1	16059949	16070046	CELA2A, TMEM82
chr1	16155997	16164227	CELA2A
chr1	16465112	16554574	SPATA21
chr1	17019382	17051807	ESPNP
chr1	17215262	17307660	NBPF1
chr1	17423509	17460118	PADI2, SDHB
chr1	17551677	17582041	SDHB
chr1	17828633	17961597	PADI2
chr1	19233804	19289265	ALDH4A1
chr1	19333613	19427107	ALDH4A1
chr1	19661379	19808674	
chr1	19909319	19947764	NBL1
chr1	19965334	19984929	AKR7L
chr1	20113568	20144846	RNF186
chr1	20796697	20823686	CAMK2N1
chr1	21574800	21669795	
chr1	21932541	22000272	CDC42
chr1	22213631	22265779	CDC42
chr1	22554966	22591190	CELA3B
chr1	23000040	23122584	
chr1	23744351	23780387	ASAP3, FUCA1, TCEA3
chr1	23840199	23920440	ID3
chr1	24117166	24138236	ZNF436
chr1	24426788	24449990	
chr1	25217166	25289408	C1orf63
chr1	25856096	25901176	C1orf63
chr1	26592392	26629600	
chr1	26662449	26705144	PIGV
chr1	26855685	26870766	PDIK1L
chr1	27017777	27034137	
chr1	27155163	27208661	PIGV
chr1	27316744	27360530	SYTL1
chr1	27424877	27489140	PIGV

chr1	27813161	27927216	XKR8
chr1	28184862	28219220	STX12
chr1	31202847	31297732	SNRNP40
chr1	31885596	31919499	SERINC2
chr1	31974209	32057981	TINAGL1
chr1	32127999	32154793	COL16A1
chr1	32387789	32429778	BAI2
chr1	32754249	32819404	BSDC1
chr1	33177547	33241527	KIAA1522
chr1	33342282	33373754	ADC
chr1	33390274	33490740	TMEM54
chr1	33772194	33816350	
chr1	36614482	36653881	
chr1	37936562	37964770	
chr1	39570164	39599196	
chr1	40388079	40456214	
chr1	40494460	40525992	
chr1	41823076	41899496	
chr1	41916513	41984388	
chr1	42611417	42639350	GUCA2B, RIMKLA, GUCA2A
chr1	43389469	43447054	C1orf210
chr1	43810360	43839648	C1orf210
chr1	43971999	44054819	SLC6A9
chr1	44448905	44509737	SLC6A9
chr1	44701316	44760395	ERI3
chr1	45097037	45154968	
chr1	45270933	45276806	ERI3
chr1	46631993	46660693	
chr1	46766371	46773007	
chr1	47644434	47659486	PDZK1IP1
chr1	47787864	47802416	
chr1	47896527	47918279	FOXD2
chr1	48150864	48195520	
chr1	51761624	51802404	
chr1	53758670	53794340	NDC1
chr1	54713922	54891305	TCEANC2
chr1	55024214	55073822	C1orf177

chr1	55241442	55282938	TTC22, ACOT11, C1orf177
chr1	59220979	59252104	TACSTD2
chr1	59268562	59291681	JUN
chr1	59314538	59409852	
chr1	61508480	61523688	
chr1	65530848	65534992	
chr1	68154381	68236593	
chr1	68268228	68299628	
chr1	85740062	85774046	MCOLN2
chr1	94031857	94090070	BCAR3
chr1	95239197	95290129	
chr1	109674773	109729623	GNAT2
chr1	110299715	110366988	GNAI3
chr1	110419656	110490886	
chr1	111734321	111748176	
chr1	113230530	113250444	
chr1	113733834	113799569	
chr1	116914777	116952673	ATP1A1
chr1	116996706	117060164	MAB21L3
chr1	120306701	120361067	
chr1	145415373	145457367	PDZK1
chr1	150530373	150553183	C1orf51
chr1	150568356	150595388	C1orf51
chr1	150942968	150952107	SETDB1, SEMA6C, PSMD4
chr1	151466905	151491681	SEMA6C
chr1	151502988	151522989	CELF3
chr1	151907359	151974283	OAZ3
chr1	152006566	152024217	CRNN
chr1	153478933	153511730	S100A6
chr1	153534043	153550588	S100A4
chr1	153571029	153590531	S100A6
chr1	153735777	153778969	
chr1	153915344	153937491	NUP210L
chr1	154374178	154461718	IL6R
chr1	154942465	154949259	GBA
chr1	154961047	154995970	ZBTB7B

chr1	155009246	155026555	ZBTB7B
chr1	155050463	155059415	EFNA3
chr1	155095442	155113225	GBA
chr1	155161643	155165270	GBA
chr1	155899428	155917420	SLC25A44
chr1	155931349	155979411	C1orf85
chr1	156046329	156117267	C1orf85
chr1	156425720	156498221	C1orf85
chr1	156625898	156664775	RHBG, NES, BCAN, CRABP2, PAQR6, PEAR1, PMF1-BGLAP
chr1	156711981	156730297	C1orf85
chr1	159815583	159860425	CCDC19
chr1	159891304	159896086	
chr1	160981786	160992848	APOA2
chr1	161042898	161060144	USP21
chr1	161358989	161380324	ITLN2
chr1	164545582	164653369	
chr1	167032473	167105969	GPA33
chr1	167180129	167199590	
chr1	167681244	167727398	
chr1	169013166	169091553	
chr1	180123204	180143474	QSOX1
chr1	181057187	181130317	
chr1	182352118	182367034	TEDDM1
chr1	197740123	197757457	
chr1	199990289	200023200	NR5A2
chr1	200856680	200899634	C1orf106
chr1	200961014	200998342	
chr1	201260301	201282790	PKP1
chr1	201322392	201383899	PKP1
chr1	201417175	201486897	
chr1	201973808	202104958	ELF3
chr1	202752017	202784691	
chr1	203236601	203316915	FMOD

chr1	204042149	204107886	REN
chr1	204228245	204368586	
chr1	204412490	204482785	
chr1	205216898	205291308	
chr1	205399713	205519696	LEMD1
chr1	205532653	205568500	SLC45A3
chr1	205628438	205650044	ELK4, MFSD4
chr1	206831654	206910943	IL19
chr1	207098037	207154650	
chr1	207174748	207208974	IL19
chr1	207223454	207244245	
chr1	207911155	207929904	
chr1	207979272	208043352	
chr1	208274061	208359483	
chr1	208373939	208424650	PLXNA2
chr1	209769236	209833030	
chr1	211502176	211527688	
chr1	211687472	211723080	
chr1	211776506	211828545	TRAF5
chr1	212768614	212783470	ANGEL2
chr1	223892515	223938872	
chr1	225596010	225668822	DNAH14
chr1	226029826	226110273	
chr1	226288057	226325426	TMEM63A
chr1	226815255	226927099	
chr1	228072907	228141375	ZNF678
chr1	228246419	228278681	ZNF678
chr1	228326981	228331088	ZNF678
chr1	228349132	228354871	RNF187
chr1	228940289	229004627	RHOU
chr1	229228138	229309977	
chr1	229356331	229390079	SPHAR
chr1	230209480	230297332	
chr1	230864436	230904943	
chr1	231531350	231559064	
chr1	232714463	232771391	
chr1	233743752	233761980	
chr1	234657523	234695975	SLC35F3
chr1	234724833	234760650	SLC35F3

chr1	234834348	234888304	SLC35F3
chr1	234951286	235069151	
chr1	235088748	235158297	
chr1	244209587	244240054	
chr1	244995993	245002365	C1orf101
chr1	245115380	245136153	C1orf101
chr1	249137064	249168535	
chr10	634473	710051	
chr10	972598	1001352	
chr10	3777459	3830408	
chr10	3846361	3854638	
chr10	3867604	3950023	
chr10	5506961	5706245	ASB13
chr10	11704540	11759069	
chr10	14564566	14667661	
chr10	15331568	15397491	FAM171A1
chr10	21455548	21464438	
chr10	24737364	24784244	
chr10	25004669	25021540	KIAA1217
chr10	27519398	27532774	
chr10	30706423	30726113	
chr10	32329666	32349392	
chr10	32383993	32479292	
chr10	32621022	32674058	
chr10	33227489	33272835	
chr10	43832131	43917416	
chr10	46990958	47058393	NPY4R
chr10	70976222	70993459	CCAR1
chr10	71081085	71149948	VPS26A
chr10	71164584	71267921	
chr10	71595936	71678030	PPA1
chr10	71885733	71903754	EIF4EBP2
chr10	72969762	73056436	UNC5B
chr10	73356961	73422741	UNC5B
chr10	73444381	73542810	
chr10	74002542	74100641	ANAPC16
chr10	74420932	74463640	
chr10	75603323	75677591	
chr10	76946014	76973090	VDAC2

chr10	76985609	77056330	ZNF503
chr10	77154927	77169743	ZNF503
chr10	79623645	79682923	
chr10	80710759	80737653	ZMIZ1
chr10	80804683	80922165	
chr10	80981446	81119139	EIF5AL1
chr10	81135308	81202484	SFTPA1
chr10	81895869	81968780	DYDC1
chr10	82187931	82269537	
chr10	85918778	85965078	GHITM, C10orf99
chr10	88424408	88476592	WAPAL
chr10	88727013	88732486	ADIRF
chr10	88851230	88865926	GLUD1
chr10	89621087	89629308	CFL1P1
chr10	90639024	90664381	
chr10	95172495	95236263	
chr10	95301521	95351810	FFAR4
chr10	96990557	97074536	
chr10	98337456	98394081	
chr10	99086316	99097482	FRAT2
chr10	99457428	99486837	FRAT2
chr10	102122439	102137454	
chr10	102755594	102779103	PAX2
chr10	102796517	102832180	
chr10	103872182	103881363	FGF8
chr10	104158359	104183191	ARL3
chr10	104353343	104438983	
chr10	105302757	105380116	CNNM2
chr10	105395066	105612788	NEURL1
chr10	105646533	105683374	NEURL1
chr10	106059240	106110982	GSTO1
chr10	111753970	111773182	
chr10	111962979	111981869	MXI1
chr10	112113616	112191012	
chr10	112562258	112639452	PDCD4
chr10	114090057	114173118	
chr10	114705275	114722724	
chr10	114760323	114857045	
chr10	115704372	115722023	

chr10	116254043	116284052	
chr10	119102004	119135732	
chr10	121011545	121080495	BAG3, NANOS1, GRK5
chr10	123686351	123696391	NSMCE4A
chr10	123773041	123824543	FGFR2
chr10	123860764	123910041	ATE1
chr10	126275072	126437053	METTL10
chr10	126687348	126851492	METTL10
chr10	129670011	129721503	
chr10	134197375	134245029	
chr10	134257773	134301784	
chr10	134384253	134426321	LRRC27
chr10	135070841	135093527	ZNF511
chr10	135156522	135179866	CALY, MTG1, PRAP1
chr11	199803	210282	
chr11	312970	318010	ANO9, IFITM2, IFITM1
chr11	390974	418670	ANO9
chr11	438101	448828	ATHL1, DEAF1, TMEM80, CEND1, ANO9
chr11	487117	519196	
chr11	557515	564610	ANO9
chr11	606195	622242	
chr11	701921	711865	ANO9
chr11	818110	822789	ANO9
chr11	823355	835562	CEND1
chr11	1027798	1158380	
chr11	1214325	1285146	
chr11	1525034	1597870	TOLLIP
chr11	1772068	1825281	ASCL2
chr11	1850339	1864639	ASCL2

chr11	2007426	2018993	KCNQ1, CD81, ASCL2, KRTAP5-5
chr11	2222838	2227981	ASCL2
chr11	2228439	2233334	ASCL2
chr11	2233511	2240175	ASCL2
chr11	2277708	2293051	ASCL2
chr11	2531969	2557416	ASCL2
chr11	2745510	2769952	CD81, ASCL2
chr11	2782893	2865875	CD81
chr11	2922397	2926733	TRPM5
chr11	2930168	2936397	OSBPL5
chr11	2947884	2955533	SLC22A18
chr11	3143708	3192459	ZNF195
chr11	7593139	7644873	
chr11	8708696	8755650	ST5
chr11	8768419	8836787	
chr11	10306635	10374218	
chr11	12065965	12303424	
chr11	16921358	16947833	KCNJ11
chr11	16967632	17032859	KCNJ11
chr11	27485463	27494502	
chr11	34066224	34077172	
chr11	34171953	34296972	
chr11	34605603	34627752	
chr11	34641466	34676856	
chr11	35159997	35201316	
chr11	36366405	36403697	
chr11	44589671	44656439	CD82
chr11	44952460	45004436	TP53I11
chr11	45167037	45204540	PRDM11, TSPAN18
chr11	46258923	46340902	ATG13
chr11	47395923	47437968	ATG13
chr11	48004523	48088987	
chr11	57037148	57094593	ZDHHC5
chr11	57528674	57569477	

chr11	58939074	58975027	DTX4, GLYATL1
chr11	59521144	59568809	
chr11	60885373	60933207	ZP1
chr11	61298131	61396818	DAK
chr11	61460952	61469382	CPSF7
chr11	61519574	61526232	MYRF
chr11	61721960	61749932	FADS3
chr11	62303222	62328979	HNRNPUL2
chr11	63529313	63537924	C11orf84
chr11	63602327	63659507	
chr11	63682671	63690515	RTN3
chr11	64004653	64012011	MARK2
chr11	64084259	64091917	MARK2
chr11	64654508	64663543	SNX15
chr11	65043034	65085130	DPF2, CDC42EP2, TM7SF2, LTBP3, MAP4K2, KCNK7, EHBP1L1, SIPA1
chr11	65146042	65153316	MAP3K11
chr11	65182698	65196358	MAP3K11
chr11	65235731	65278300	KCNK7
chr11	65336369	65346081	SYVN1
chr11	65541984	65595884	DPF2
chr11	65609879	65638159	OVOL1
chr11	66621798	66651079	
chr11	66799161	66863232	PITPNM1
chr11	66880913	66897995	RBM14
chr11	67000685	67058673	CABP4
chr11	67118214	67125134	POLD4
chr11	67138085	67142641	CABP4
chr11	67231049	67237376	CABP4
chr11	67253402	67276815	PITPNM1
chr11	67396050	67415560	CABP4
chr11	67775272	67783134	UNC93B1

chr11	67803411	67811072	
chr11	67857262	67933103	CHKA
chr11	67963386	67984625	SUV420H1
chr11	68063591	68237978	
chr11	68577616	68638315	CPT1A, TPCN2
chr11	68861560	68905716	IGHMBP2, TPCN2, MRGPRF
chr11	69036308	69080715	IGHMBP2, ORAOV1, TPCN2, MRGPRF
chr11	69142617	69324571	CCND1, ORAOV1, TPCN2, FGF19, MRGPRF
chr11	69451044	69483535	CCND1, ORAOV1
chr11	69777158	69847417	CTTN, PPFIA1, ORAOV1, FGF19, FADD
chr11	70961547	71022977	
chr11	71932563	71952314	
chr11	72414607	72501733	ATG16L2
chr11	72518642	72544162	
chr11	72884579	72953894	
chr11	73076627	73104220	
chr11	73668076	73731342	
chr11	74854195	74915339	SLCO2B1
chr11	75012037	75064571	MOGAT2
chr11	75176812	75248626	GDPD5, NEU3
chr11	75264295	75306314	SERPINH1
chr11	76447841	76517502	TSKU
chr11	76773786	76787721	CAPN5
chr11	76796563	76803020	TSKU
chr11	77170698	77186470	

chr11	94258920	94283685	FOLR4
chr11	117052240	117100620	APOA5
chr11	117679662	117714818	CEP164, FXYD6, SCN2B, SCN4B
chr11	117944260	117963999	
chr11	118480089	118513590	MPZL3
chr11	118659132	118663498	
chr11	118740466	118811184	DDX6
chr11	119227146	119247355	USP2, PDZD3, C2CD2L
chr11	119533622	119620178	
chr11	119980432	120018196	TRIM29
chr11	120039014	120105066	OAF
chr11	121315953	121353712	
chr11	124615370	124639616	SIAE
chr11	126180616	126350742	ST3GAL4
chr11	128317351	128389642	
chr11	129856355	129874646	
chr11	130011761	130090730	APLP2
chr12	607333	620505	SLC6A12
chr12	633556	685948	SLC6A12
chr12	718228	756396	
chr12	1682523	1718555	
chr12	1764325	1791236	LRTM2
chr12	1904657	1956059	
chr12	2210461	2280312	CACNA2D4, DCP1B, CACNA1C, LRTM2
chr12	3813953	3867210	EFCAB4B
chr12	4378093	4417824	
chr12	6267425	6351305	PLEKHG6
chr12	6418899	6423651	LPAR5
chr12	6441984	6453122	PLEKHG6
chr12	6468473	6473930	PLEKHG6
chr12	6477590	6487215	SCNN1A
chr12	6641233	6661870	

chr12	6712215	6724398	
chr12	7033601	7038885	
chr12	7063241	7074431	
chr12	11801391	11839716	
chr12	12669886	12716384	
chr12	12856536	12911664	
chr12	12928123	12963912	GPRC5D
chr12	13023563	13064122	GPRC5A
chr12	13515032	13544386	
chr12	14338390	14374087	
chr12	26253869	26288438	
chr12	31470814	31479941	DDX11
chr12	32543708	32556907	FGD4
chr12	33022694	33073384	
chr12	46120396	46129994	
chr12	46649613	46664328	SLC38A1
chr12	46761555	46797433	RPAP3
chr12	48193261	48233528	RPAP3
chr12	48252290	48307206	VDR
chr12	48332134	48377560	ASB8
chr12	49147496	49219129	ADCY6
chr12	49450777	49455187	
chr12	50260667	50295089	AQP6
chr12	50632112	50672288	
chr12	50901291	50962394	
chr12	51656594	51670845	HIGD1C
chr12	51779978	51802516	GALNT6
chr12	52205241	52265790	
chr12	52403001	52481069	KRT82
chr12	52536284	52564415	KRT84
chr12	53253487	53322649	MFSD5
chr12	53335421	53403264	MFSD5
chr12	53437709	53467279	CSAD
chr12	53609386	53636317	ESPL1
chr12	53717912	53784473	MFSD5
chr12	56117619	56139964	
chr12	56320233	56334550	RDH5
chr12	56472617	56481929	RDH5
chr12	56536023	56559443	RDH5
chr12	57016619	57034040	

chr12	57063053	57094665	
chr12	57479842	57500211	R3HDM2
chr12	57519739	57575138	KIF5A
chr12	57910582	57936584	
chr12	58225534	58293317	R3HDM2
chr12	71546847	71560616	
chr12	89727142	89785423	
chr12	102084835	102107721	CHPT1
chr12	106620916	106644657	TCP11L2
chr12	106667456	106705140	TCP11L2
chr12	109116029	109129394	
chr12	109871572	109906385	
chr12	110662371	110708258	
chr12	110994563	111043503	
chr12	111827599	111888807	ACAD10
chr12	112179011	112230760	TMEM116
chr12	112243969	112289496	TMEM116
chr12	113338786	113379601	CCDC42B
chr12	113554756	113592779	RASAL1, CCDC42B
chr12	113631740	113709983	CCDC42B
chr12	115093425	115142393	TBX3, TESC, RNFT2
chr12	117463599	117557001	TESC, RNFT2
chr12	118489390	118502783	
chr12	120105481	120132791	PRKAB1
chr12	120636380	120680464	
chr12	121096466	121131147	C12orf43
chr12	121392155	121423524	SPPL3
chr12	121650947	121685811	P2RX4
chr12	122223345	122245252	BCL7A
chr12	122467247	122530502	DIABLO
chr12	122580629	122625739	DIABLO
chr12	123310119	123406755	CCDC62
chr12	123543736	123636760	CCDC62
chr12	124836999	124951266	
chr12	124964249	125050163	DHX37
chr12	125066524	125263109	SCARB1

chr12	125299360	125428074	SCARB1
chr12	132951183	133031675	EP400
chr12	133048434	133101921	ZNF605
chr12	133396203	133415898	PXMP2
chr13	20691989	20703211	
chr13	27522316	27597035	USP12
chr13	28016432	28030077	GSX1
chr13	28479626	28498912	PDX1
chr13	28526733	28555860	
chr13	30944754	30996971	
chr13	31354551	31446668	HSPH1, USPL1
chr13	41237892	41242018	WBP4
chr13	41537775	41597025	
chr13	42600548	42616099	AKAP11
chr13	50695590	50705124	KPNA3
chr13	72437884	72449334	
chr13	73611749	73666270	
chr13	74703519	74710680	
chr13	80910634	80917643	
chr13	97873693	97931632	
chr13	99083225	99245634	
chr13	99841992	99885096	SLC15A1
chr13	100608044	100649785	PCCA
chr13	106736061	106757196	
chr13	107172698	107188987	
chr13	110419448	110455803	IRS2
chr13	111155382	111234492	CARKD
chr13	111252364	111299825	CARKD
chr13	111559728	111581992	CARKD
chr13	113324635	113382209	F7, ATP11A, TUBGCP3, MCF2L, F10, PCID2

chr13	113526714	113566003	F7, ATP11A, TUBGCP3, MCF2L, F10, PCID2, CUL4A, LAMP1
chr13	113607947	113677177	MCF2L, F10, PCID2, CUL4A, DCUN1D2, LAMP1, KARSP2
chr13	114427335	114521485	DCUN1D2, TMEM255B, LAMP1, TFDP1
chr13	114534188	114584381	CDC16, DCUN1D2, UPF3A
chr13	114739908	114932567	CDC16, UPF3A, TMEM255B, GRK1, CHAMP1
chr14	21131506	21158048	TMEM253
chr14	21564681	21573947	ZNF219
chr14	23006813	23040130	
chr14	24885885	24909709	
chr14	34482763	34533357	NPAS3
chr14	35340799	35344775	
chr14	35799919	35875246	NFKBIA
chr14	38052216	38072548	TTC6
chr14	50090494	50110091	
chr14	50233313	50238684	
chr14	50328116	50387669	KLHDC2
chr14	50412382	50490957	
chr14	50499354	50535797	

chr14	54409834	54430681	BMP4
chr14	55031455	55035147	
chr14	55543370	55601049	LGALS3
chr14	55744059	55825561	LGALS3
chr14	61739169	61749050	
chr14	61927818	62037651	SYT16
chr14	64955758	64976110	
chr14	65085747	65233880	HSPA2
chr14	65395006	65440194	HSPA2
chr14	65689140	65772058	CHURC1-FNTB
chr14	67864295	67955825	TMEM229B
chr14	68968180	68996099	ACTN1
chr14	69013307	69079250	
chr14	69225159	69291477	
chr14	69380265	69447558	
chr14	70072262	70194859	
chr14	71786661	71807764	
chr14	74185477	74274163	ENTPD5
chr14	74427852	74495599	ENTPD5
chr14	75401472	75454960	PGF
chr14	75599429	75674050	FOS
chr14	75717115	75782305	FOS
chr14	75904523	75944066	FOS
chr14	77412867	77432371	
chr14	77461667	77568858	
chr14	89879722	89896100	FOXN3
chr14	90847362	90851754	
chr14	90966987	91023449	TTC7B
chr14	91817703	91881574	RPS6KA5
chr14	92958237	93040449	
chr14	93468082	93583489	TMEM251
chr14	94848020	94862793	SERPINA1
chr14	95696947	95828465	CLMN
chr14	95906301	95990836	GLRX5
chr14	99696766	99741599	BCL11B
chr14	99983921	100089112	SETD3
chr14	100569955	100660583	
chr14	100704039	100720064	

chr14	100848393	100910735	
chr14	100989114	101036015	
chr14	101971588	102025141	
chr14	102171937	102201097	
chr14	102547539	102564353	
chr14	102930302	103061763	
chr14	103227150	103294455	
chr14	103367432	103415466	AMN
chr14	103978637	104018685	ZFYVE21
chr14	104158621	104197021	ZFYVE21
chr14	104320927	104376746	RD3L
chr14	104546791	104586533	C14orf180
chr14	104686749	104713574	RD3L
chr14	105116452	105174971	
chr14	105309426	105362522	
chr14	105423465	105449860	C14orf79
chr14	105498203	105561011	C14orf79
chr14	105621866	105674110	JAG2
chr14	105758950	105830896	
chr14	105944150	105959031	
chr14	107250879	107259790	
chr15	29962135	29982408	FAM189A1
chr15	31493186	31528909	
chr15	31546526	31573312	
chr15	31616912	31698718	
chr15	31725421	31791946	
chr15	34651240	34660801	LPCAT4
chr15	39876202	39938272	FSIP1
chr15	40328510	40401905	BMF, C15orf52
chr15	40634403	40643032	PHGR1
chr15	40727561	40759825	IVD
chr15	41054990	41088148	
chr15	41135119	41140730	CHP1
chr15	41765875	41806408	ITPKA
chr15	42325351	42447162	
chr15	43414495	43430084	TGM7
chr15	44998105	45023192	
chr15	45454839	45480972	SHF

chr15	45716708	45749759	SLC30A4
chr15	45923497	45953467	SLC30A4
chr15	57576544	57636050	MYZAP
chr15	57829356	57911847	
chr15	59535671	59672981	
chr15	59821634	59863374	GCNT3
chr15	60654602	60701728	
chr15	63329419	63385012	
chr15	63637454	63689603	CA12
chr15	63729366	63814439	APH1B
chr15	64257574	64322400	DAPK2
chr15	65127549	65191429	
chr15	65372000	65393928	SLC51B
chr15	66997057	67073974	SMAD6
chr15	67315989	67483410	
chr15	68479899	68503417	FEM1B
chr15	69104516	69114591	
chr15	69583168	69623396	PAQR5
chr15	70381141	70406522	
chr15	70767028	70825700	
chr15	72514662	72530599	
chr15	74666150	74735360	PML
chr15	74890326	74911842	CSK
chr15	75470043	75516959	SIN3A
chr15	75938741	75996895	UBE2Q2
chr15	77280658	77331255	RCN2
chr15	78326848	78398281	
chr15	80951964	81004924	
chr15	85276709	85307822	SLC28A1
chr15	85359945	85411716	SLC28A1
chr15	86293117	86344492	
chr15	89158014	89193681	
chr15	89631585	89694599	RLBP1
chr15	90540912	90650466	AP3S2
chr15	90752587	90759016	AP3S2, GDPGP1, ANPEP, C15orf38, C15orf38-AP3S2

chr15	91356656	91420850	
chr15	93157113	93211680	
chr15	93344399	93400683	
chr15	93437871	93469422	ST8SIA2
chr15	96864752	96904833	
chr15	99942642	99995817	
chr16	118069	136722	RHBDF1, NPRL3
chr16	356946	415139	
chr16	428699	437016	WFIKKN1
chr16	573964	582164	WFIKKN1
chr16	582919	587823	WFIKKN1
chr16	636097	645846	RAB40C
chr16	690358	701750	GNG13
chr16	724323	731964	WFIKKN1
chr16	845690	892971	UBE2I
chr16	1308919	1363314	UBE2I, SSTR5
chr16	1457757	1491537	UBE2I
chr16	1942780	2000825	RPL3L
chr16	2027583	2037544	MEIOB
chr16	2131635	2144296	MEIOB
chr16	2205619	2214366	PDPK1
chr16	2561938	2567661	
chr16	2805025	2847185	CCDC64B
chr16	3054054	3061124	CLDN6, CLDN9
chr16	3069834	3074514	TNFRSF12A, PKMYT1
chr16	3193126	3248938	ZNF200
chr16	3686587	3707943	DNASE1
chr16	4335195	4398116	TFAP4
chr16	4644232	4669625	GLYR1
chr16	4684153	4753034	CDIP1
chr16	4963908	4998478	PPL
chr16	8943472	8986349	METTL22
chr16	9002905	9063032	
chr16	10641327	10723661	
chr16	11290981	11360138	SOCS1

chr16	11651637	11735371	PRM2
chr16	11816017	11848057	PRM2
chr16	11876173	11893946	
chr16	14728240	14767544	NTAN1
chr16	17352730	17375255	
chr16	18999769	19019920	
chr16	19119247	19148441	
chr16	19394248	19449678	
chr16	22199573	22251193	PDZD9
chr16	22364488	22386854	
chr16	23302955	23377994	SCNN1B, CHP2
chr16	23698811	23724946	CHP2
chr16	27200578	27256196	IL4R
chr16	27324440	27342571	
chr16	28488682	28512371	SULT1A1, SULT1A2
chr16	29815835	29822485	
chr16	30123335	30135353	MAPK3
chr16	30667396	30681967	
chr16	30816439	30856714	KAT8
chr16	31137030	31147852	C16orf58
chr16	31149383	31154958	C16orf58
chr16	31487207	31500133	C16orf58
chr16	48635742	48662738	
chr16	49886677	49893398	
chr16	50278321	50347909	PAPD5
chr16	50572354	50667579	NKD1
chr16	50698862	50754682	
chr16	52578178	52613011	
chr16	53064422	53096863	
chr16	53119101	53136836	
chr16	53163214	53168804	
chr16	56945242	56968944	MT1JP
chr16	56998302	57076712	
chr16	57118390	57188393	RSPRY1
chr16	57294197	57351317	HERPUD1
chr16	57503872	57521139	RSPRY1
chr16	57611459	57682895	KIFC3

chr16	57791408	57861419	CSNK2A2, CCL22, KIFC3
chr16	58055117	58083295	
chr16	66542493	66561415	CKLF
chr16	67264501	67281550	ATP6V0D1
chr16	67423853	67447927	LRRC36
chr16	67460809	67504574	ATP6V0D1, HSD11B2
chr16	68103403	68122765	NRN1L
chr16	68260190	68274411	SMPD3
chr16	68290935	68324768	
chr16	68384973	68432029	SMPD3
chr16	68444955	68469934	
chr16	68730192	68828253	SMPD3
chr16	69596240	69602314	TANGO6
chr16	69856684	69897011	
chr16	70413518	70485050	IL34
chr16	70713976	70803361	VAC14
chr16	71924300	71932810	PHLPP2
chr16	73066783	73106221	ZFHX3
chr16	74747247	74815204	LDHD
chr16	75085989	75147825	
chr16	75258266	75302516	TMEM170A
chr16	75540824	75584967	CHST5, LDHD, CHST6
chr16	81299560	81312418	ATMIN
chr16	81465362	81687328	
chr16	81718415	81789855	
chr16	84362904	84424162	ATP2C2
chr16	85013578	85303424	GSE1
chr16	85317614	85431400	GSE1
chr16	85449100	85526770	GSE1
chr16	85546638	85715065	
chr16	85925110	86032329	
chr16	87394373	87423188	

chr16	87489743	87552690	ZCCHC14
chr16	87860460	87932015	SLC7A5, ZCCHC14
chr16	87978709	88000830	
chr16	88443224	88621560	ZC3H18
chr16	88681562	88714823	
chr16	88971595	89008487	
chr16	89114297	89190040	
chr16	89208436	89277923	ANKRD11
chr16	89360311	89573034	
chr16	89630327	89708513	DPEP1, GAS8, SPATA33, MC1R
chr16	89786044	89790774	
chr16	89887868	89930922	CDK10
chr17	170735	207830	GLOD4
chr17	964335	1050931	PITPNA
chr17	1101108	1135946	PITPNA
chr17	1386109	1397462	ABR
chr17	1411167	1421098	TUSC5
chr17	1449335	1525362	YWHAE
chr17	1543204	1555138	ABR
chr17	1633308	1691678	HIC1
chr17	1956802	2005409	
chr17	2286152	2316398	
chr17	2607521	2634988	PAFAH1B 1
chr17	2658328	2718439	PAFAH1B 1, SRR, OR1G1, OR3A2, OR1D5, OR1D2
chr17	3763128	3839913	ATP2A3
chr17	3856496	3892930	ATP2A3
chr17	4033536	4048892	ATP2A3
chr17	4377104	4447820	MYBBP1A
chr17	4847470	4854915	ZFP3
chr17	6448696	6478672	PITPNM3
chr17	6915193	6919266	

chr17	7157918	7195844	YBX2
chr17	7455758	7472180	EFNB3
chr17	7736374	7755771	SLC25A35
chr17	8053741	8059831	KRBA2
chr17	10676053	10719009	TMEM220
chr17	15847554	15895545	TTC19
chr17	16188246	16209563	
chr17	16346060	16393603	TTC19
chr17	16884214	17030840	ZNF287
chr17	17365141	17483447	COPS3
chr17	17566759	17696758	DRG2, PEMT, COPS3, GID4
chr17	17710689	17763585	GID4
chr17	17795500	17864058	
chr17	18864569	18923164	PRPSAP2
chr17	19268613	19306669	PRPSAP2
chr17	21147218	21201645	CCDC144NL
chr17	25641314	25681602	
chr17	25782737	25977397	
chr17	26120397	26151772	NOS2
chr17	26797440	26877241	NLK
chr17	27056274	27088768	FAM222B
chr17	27275350	27323531	TLCD1
chr17	27451350	27508908	ERAL1
chr17	27892391	27896886	TIAF1
chr17	27913646	27922097	GIT1
chr17	29776133	29841499	SUZ12
chr17	29868692	29926601	
chr17	31094931	31201094	TMEM98
chr17	34090986	34129390	
chr17	34947717	34965729	
chr17	36022324	36112743	
chr17	36570831	36623254	RPL23
chr17	36713468	36774104	
chr17	36857346	36863504	
chr17	37024425	37079151	
chr17	37779956	37790775	ERBB2

chr17	37827160	37837462	FBXL20
chr17	37851220	37876195	RAPGEFL1
chr17	37892232	37913020	THRA
chr17	38214419	38291144	THRA
chr17	38327873	38351879	RAPGEFL1
chr17	38462778	38514141	LRRC3C
chr17	38598504	38717200	TNS4
chr17	39682460	39687590	KRT9
chr17	39773577	39847775	KRT9
chr17	39911008	39965227	RAB5C
chr17	40660962	40711847	RAMP2
chr17	40819122	40832643	G6PC
chr17	41437009	41447440	MEOX1
chr17	41604728	41670017	VAT1
chr17	42142360	42196719	SOST
chr17	42267937	42300185	C17orf105
chr17	43203803	43210810	ARHGAP27, PLCD3
chr17	43246646	43251926	DBF4B
chr17	43298334	43345181	
chr17	43361630	43419461	PLCD3
chr17	43448737	43525469	PLCD3
chr17	44257573	44291311	
chr17	45886253	45900391	PRR15L
chr17	45919982	45966551	CDK5RAP3, SP6
chr17	46021753	46046385	SP6
chr17	46652361	46661153	SKAP1
chr17	46678428	46685618	SKAP1
chr17	46685827	46707205	HOXB6, HOXB5, SKAP1, HOXB9
chr17	46707317	46725544	SKAP1
chr17	46795579	46807735	PRAC1, HOXB13
chr17	47928174	48041573	DLX3, DLX4
chr17	48104548	48157833	PDK2

chr17	48176137	48195174	PDK2
chr17	48223171	48230448	DLX4
chr17	48334907	48368245	TMEM92
chr17	48609481	48629133	PDK2
chr17	48696124	48781740	SPATA20
chr17	48909993	48947019	SPATA20, RSAD1
chr17	48960368	49034364	SPATA20
chr17	49195287	49210944	
chr17	54958902	54994593	
chr17	55161780	55198568	
chr17	55478973	55541126	MRPS23
chr17	55928476	56033391	RNF43
chr17	56407935	56417941	RNF43
chr17	57442508	57459901	
chr17	57904169	57934071	
chr17	59467114	59503640	INTS2, BRIP1
chr17	59520418	59574250	TBX4
chr17	61505427	61530049	TCAM1P
chr17	62763527	62791069	POLG2
chr17	63510723	63591127	
chr17	70106480	70124078	
chr17	70367242	70537149	
chr17	70556570	70632645	
chr17	71263731	71313768	
chr17	72368428	72480556	
chr17	72736969	72766975	
chr17	72937453	72988378	
chr17	73069985	73125224	SLC16A5
chr17	73519791	73524830	LLGL2
chr17	73529524	73542716	ACOX1
chr17	73542870	73547822	ACOX1
chr17	73568797	73577073	LLGL2, ACOX1, SMIM5, SMIM6, CDK3, MYO15B

chr17	73604926	73617522	ACOX1, CDK3, MYO15B
chr17	73634586	73644264	ACOX1
chr17	73674822	73751967	
chr17	73765374	73783587	UBALD2
chr17	73805462	73845266	
chr17	73865675	73875420	
chr17	73990524	74001796	ACOX1, CDK3
chr17	74233734	74276263	ACOX1
chr17	74485442	74490614	RHBDF2
chr17	74625305	74713030	ST6GALNAC1
chr17	75093997	75143308	METTL23
chr17	75275552	75486474	
chr17	76120080	76130605	
chr17	76164033	76173337	
chr17	76309704	76362807	
chr17	76377008	76417713	DDC8
chr17	76988200	76995813	CANT1
chr17	77064697	77166637	USP36
chr17	77763241	77790058	CBX8
chr17	77803067	77841269	CBX8, CBX4
chr17	77881082	77925857	CARD14, CBX8, CBX4, TBC1D16
chr17	77948497	78005576	CBX8, TBC1D16, CBX2, CBX4
chr17	78226455	78263038	CBX8
chr17	78419066	78486066	TBC1D16
chr17	78932703	78978711	
chr17	78991762	79075666	BAIAP2

chr17	79301713	79306416	BAIAP2, TSPAN10, TMEM105, HGS, C17orf70, OXLD1, FAM195B
chr17	79310370	79321005	ARL16
chr17	79369621	79374838	SIRT7
chr17	79435289	79505004	SIRT7
chr17	79667522	79709019	
chr17	79763639	79788092	CCDC137
chr17	79801055	79830999	
chr17	79948174	79980814	ASPSCR1, NOTUM
chr17	80051877	80066204	MYADML2
chr17	80151157	80206536	
chr17	80223855	80233218	
chr17	80249989	80257778	
chr17	80284309	80293682	SIRT7
chr17	80482483	80562717	
chr17	80585291	80669969	RAB40B
chr17	80793052	80880974	TBCD, ZNF750
chr17	81005827	81069196	
chr18	2635791	2659462	
chr18	3008916	3088358	
chr18	3256936	3266371	
chr18	3446204	3467197	
chr18	3583274	3626174	
chr18	11972481	12059708	IMPA2, MPPE1, CHMP1B
chr18	19597999	19642477	
chr18	19741865	19781074	
chr18	28676993	28682939	
chr18	29064876	29094028	
chr18	45550577	45704288	
chr18	46276601	46378172	
chr18	46443881	46487731	SMAD7

chr18	46500457	46527954	
chr18	47337489	47345951	C18orf32
chr18	47372132	47392148	ACAA2
chr18	55395874	55467803	NEDD4L
chr18	55842452	55915151	NEDD4L
chr18	59988044	60025459	KIAA1468
chr18	60189376	60194365	
chr18	60381757	60385355	KIAA1468
chr18	67953211	67961246	
chr18	72904616	72943333	TSHZ1
chr18	74202614	74208480	
chr18	74764624	74842527	
chr18	77438452	77483985	TXNL4A
chr18	77694286	77736594	HSBP1L1
chr19	282076	296359	ODF3L2
chr19	509538	559375	GRIN3B
chr19	656677	664392	PALM
chr19	718453	803563	STK11
chr19	923608	929931	FSTL3, PALM, ARID3A, SHC2, MUM1
chr19	950367	958117	FSTL3, CIRBP, PALM, APC2, ARID3A, SHC2, MUM1, R3HDM4
chr19	1025797	1029184	GRIN3B
chr19	1129240	1134649	WDR18
chr19	1247317	1256284	GRIN3B
chr19	1256513	1273670	MIDN
chr19	1282741	1294598	REEP6
chr19	1406202	1413839	CNN2
chr19	1744843	1808368	RPS15
chr19	1851612	1865911	
chr19	1929069	1948219	AMH

chr19	2021565	2096704	
chr19	2153810	2192227	
chr19	2232428	2238391	MKNK2
chr19	2268933	2274704	AMH
chr19	2326726	2361944	BTBD2
chr19	2476152	2681557	SF3A2
chr19	3093622	3109716	GNA11
chr19	3171695	3192696	NCLN
chr19	3334567	3405371	
chr19	3428287	3484190	TBXA2R, APBA3
chr19	3547728	3578659	
chr19	3687575	3727076	CREB3L3
chr19	4034556	4150644	MATK
chr19	4327469	4346051	CREB3L3
chr19	4361007	4395075	ATCAY
chr19	4800346	4843360	PLIN4
chr19	5843424	5892814	
chr19	6055439	6093630	
chr19	7447585	7494263	MAP2K7
chr19	8549191	8597085	CD320
chr19	9893946	9906881	ZNF561
chr19	10609398	10625040	RAVER1
chr19	10821894	10924477	DNM2
chr19	10973604	11012392	
chr19	11195872	11213766	DNM2
chr19	11230955	11263367	SMARCA4
chr19	11353793	11380147	DOCK6
chr19	12892449	12906321	IER2
chr19	13112500	13216575	
chr19	13255009	13322263	IER2
chr19	13945967	13956449	
chr19	13956585	13967073	NANOS3
chr19	13991072	14030133	CACNA1A
chr19	14088643	14135034	C19orf57
chr19	14153611	14196841	
chr19	14444164	14497035	
chr19	14615622	14634731	
chr19	15429981	15445397	
chr19	15485544	15491673	OR1I1

chr19	15939797	15949157	CYP4F8
chr19	16175036	16239139	
chr19	17189056	17268464	
chr19	17331090	17366461	CPAMD8
chr19	17422229	17468806	
chr19	17875523	17914613	PDE4C
chr19	18388904	18394729	
chr19	18428289	18435391	PDE4C
chr19	18611824	18621473	
chr19	18942170	18961749	
chr19	19475156	19601302	
chr19	33513199	33593993	CEBPA
chr19	33723635	33815979	SLC7A10
chr19	33855368	33904395	PEPD, CHST8, SLC7A10, LRP3, KCTD15
chr19	35485150	35505427	ZNF302
chr19	35599346	35623141	FXYD3, LSR
chr19	35755412	35761152	TMEM147
chr19	36182954	36231499	TMEM147
chr19	38404755	38496194	C19orf33
chr19	38752966	38759461	CATSPER G
chr19	39125397	39230893	CATSPER G
chr19	39282919	39338169	LGALS4
chr19	39616222	39661839	
chr19	39887706	39902964	
chr19	40419549	40457265	
chr19	40898619	40941282	
chr19	41105830	41131955	
chr19	41219923	41227824	PRX
chr19	41295809	41338520	CYP2B7P
chr19	41664915	41683352	RAB4B
chr19	41914345	41942398	CYP2B7P
chr19	42210448	42221456	CEACAM6
chr19	42233945	42247383	CEACAM6

chr19	42366254	42445578	
chr19	42676789	42723237	CEACAM8
chr19	42754754	42760715	LIPE
chr19	42781416	42790645	DMRTC2
chr19	44171684	44217864	
chr19	44267547	44289647	KCNN4
chr19	45183522	45207578	CEACAM16
chr19	45220249	45288652	BLOC1S3
chr19	45347676	45389453	CKM
chr19	45593294	45677813	NKPD1
chr19	45922666	45989212	FOSB
chr19	46008524	46032748	
chr19	46262286	46278443	FOSB
chr19	46365538	46386839	
chr19	47103387	47124861	
chr19	47216789	47247041	
chr19	47260797	47307254	
chr19	47578444	47636755	C5AR2
chr19	47728441	47736909	ZNF541
chr19	47983534	48027142	
chr19	48101934	48171365	EHD2, SULT2A1, MEIS3, PLA2G4C, ZNF541, SLC8A2, C5AR2, PRR24
chr19	48262056	48284829	
chr19	48811905	48837687	PLA2G4C
chr19	48983477	49006295	LMTK3
chr19	49043282	49072203	SULT2B1, LMTK3
chr19	49114785	49123342	TMEM143
chr19	49178577	49202140	ZNF114, SEC1P
chr19	49374781	49379649	SYNGR4
chr19	49463258	49480601	CGB8
chr19	49627767	49675265	
chr19	50046431	50075143	FCGRT

chr19	50136758	50146335	
chr19	50668992	50738128	NR1H2
chr19	51320574	51347864	KLK3
chr19	53425844	53450400	ERVV-2
chr19	54345892	54393382	CACNG6
chr19	54690066	54696355	KIR3DX1
chr19	54969550	54977800	KIR2DL4
chr19	54979246	54985496	KIR2DL4
chr19	55577080	55602899	TMEM150 B
chr19	55756765	55767392	
chr19	55848994	55853783	NLRP4
chr19	55894375	55899811	
chr19	56168399	56214725	PTPRH
chr19	56592157	56669915	
chr19	59065229	59071596	
chr2	1587869	1662339	
chr2	8613832	8688760	
chr2	8705570	8785328	
chr2	8816205	8834116	ID2
chr2	9349698	9425572	
chr2	9878806	9972083	ITGB1BP1, CPSF3, YWHAQ
chr2	10146514	10201123	KLF11
chr2	10259805	10311039	
chr2	10357524	10446140	CYS1
chr2	10465173	10642070	CYS1
chr2	11487649	11547488	
chr2	11882894	11903569	ROCK2
chr2	14762896	14776676	FAM84A
chr2	20291248	20442689	TTC32
chr2	20615422	20650844	SDC1
chr2	20767397	20842076	HS1BP3, GDF7
chr2	25554944	25566165	DNAJC27
chr2	26214174	26299626	
chr2	26672847	26745981	DRC1

chr2	27924636	27989801	GCKR, CAD, MPV17, EIF2B4, FNDC4, SUPT7L
chr2	28542414	28677214	
chr2	28806993	28914993	
chr2	28970251	28978251	FOSL2
chr2	36582240	36651757	
chr2	39346751	39352498	ARHGEF3 3
chr2	42273903	42369775	
chr2	43035897	43038998	EML4
chr2	43132650	43314387	HAAO
chr2	43327804	43504749	
chr2	46503123	46543729	ATP6V1E2
chr2	47072749	47110258	
chr2	47173078	47336580	
chr2	47535049	47614017	TTC7A
chr2	54750916	54832239	
chr2	60693031	60784118	
chr2	62514847	62536640	
chr2	64828384	64877868	CEP68
chr2	65080428	65095143	
chr2	65604444	65664937	
chr2	68477086	68480722	
chr2	69971637	70035271	
chr2	70159060	70239677	
chr2	70295063	70336961	ASPRV1
chr2	70367342	70371248	
chr2	74197943	74265345	MOB1A
chr2	74666483	74670292	TTC31
chr2	75060443	75103512	
chr2	85049037	85116562	RETSAT
chr2	85146148	85223840	CAPG
chr2	85531154	85555765	ELMOD3
chr2	85623710	85699394	ATOH8
chr2	88421901	88459969	FABP1
chr2	96797386	96830711	

chr2	97189470	97223131	FAM178B
chr2	97424315	97439514	CNNM4
chr2	97526110	97598121	FAM178B
chr2	98265837	98290977	TMEM131
chr2	101429505	101442449	RPL31
chr2	101850423	101884755	
chr2	103234963	103285465	SLC9A2
chr2	105986237	106029596	
chr2	106042446	106085752	
chr2	109573243	109609883	EDAR
chr2	110417091	110474383	
chr2	110855017	110874614	10-Sep
chr2	111874466	111893676	
chr2	113914222	114010081	IL36RN
chr2	114026412	114049132	IL36RN
chr2	114632201	114660496	
chr2	120160007	120199997	SCTR
chr2	120961029	121004791	RALB
chr2	121279843	121391313	TMEM185B
chr2	121942134	122061482	
chr2	127807485	127893131	PROC, BIN1, GYPC, ERCC3
chr2	128144372	128181433	LIMS2
chr2	128377586	128436836	MAP3K2
chr2	129018217	129083764	UGGT1
chr2	134876211	134947511	
chr2	151328605	151344209	
chr2	157176182	157207548	
chr2	159979586	160017653	
chr2	161061150	161085083	PLA2R1
chr2	171567847	171574960	MYO3B, GAD1, ERICH2, SP5
chr2	173087737	173119964	
chr2	173193391	173226870	
chr2	173291291	173332405	

chr2	174824174	174832589	
chr2	178103901	178138996	
chr2	190364993	190424764	COL5A2
chr2	191744139	191748184	
chr2	200294447	200349163	SATB2
chr2	201726459	201732633	
chr2	201979988	202023465	
chr2	206948329	206952121	ADAM23
chr2	208027703	208032208	CPO
chr2	208392709	208405950	PLEKHM3
chr2	208600224	208735780	PLEKHM3
chr2	218762724	218876498	RUFY4
chr2	219148537	219162345	CYP27A1
chr2	219258673	219268353	CYP27A1
chr2	219720208	219774230	TTLL4, STK36
chr2	219860927	219868129	SLC23A3
chr2	219921304	219926326	ANKZF1
chr2	219972239	219993603	FAM134A
chr2	220036739	220049138	CYP27A1
chr2	220111229	220119671	WNT10A
chr2	220290802	220360906	ANKZF1
chr2	223288466	223305254	
chr2	227655649	227666540	COL4A4
chr2	231523335	231586149	
chr2	231728493	231807493	CAB39, C2orf72
chr2	232226292	232290809	C2orf72
chr2	232461975	232581372	
chr2	233718083	233766855	ATG16L1
chr2	233842332	233883448	NEU2
chr2	233904834	233990319	
chr2	234239821	234294333	
chr2	234307968	234395713	UGT1A10
chr2	235859293	235962661	SH3BP4
chr2	236401625	236503968	
chr2	236567075	236633872	
chr2	238380339	238457019	
chr2	238568988	238653737	
chr2	239005365	239084053	SCLY

chr2	239169490	239219160	
chr2	239310380	239348264	ILKAP, ESPNL, TWIST2
chr2	240123018	240271815	
chr2	241503593	241519545	C2orf54
chr2	241540948	241546332	CAPN10, C2orf54
chr2	241810618	241873778	CAPN10, C2orf54, AQP12A, AQP12B
chr2	241895868	241951777	PASK
chr2	242242531	242260148	KIF1A
chr2	242280263	242316360	AGXT
chr2	242429052	242445571	FARP2
chr2	242704267	242763347	GAL3ST2
chr2	242784751	242844988	ING5
chr20	1305575	1319030	SDCBP2
chr20	1783026	1879655	TGM3
chr20	3775883	3789909	
chr20	5621987	5640483	CRLS1, TRMT6, GPCPD1, GPCPD1
chr20	5684386	5770892	CRLS1, GPCPD1, C20orf196
chr20	10633038	10656729	SLX4IP
chr20	16501069	16561521	SNRPB2
chr20	17546421	17553286	BFSP1
chr20	17583250	17665867	CSRP2BP
chr20	17787417	17877043	ZNF133
chr20	17902067	17921342	
chr20	17986049	18040038	
chr20	19715270	19741176	
chr20	19840549	19975049	NAA20
chr20	22548732	22567099	FOXA2
chr20	23083553	23147505	NXT1
chr20	23336843	23343803	

chr20	24997114	25072191	ABHD12, APMAP, ACSS1
chr20	25175129	25241923	
chr20	25257781	25341866	ZNF337
chr20	30147263	30200833	ID1
chr20	30248842	30311886	FOXS1
chr20	30789745	30845916	MYLK2, CCM2L, PLAGL2, C20orf112
chr20	31033456	31129997	POFUT1
chr20	32357743	32387396	RALY
chr20	32401625	32459298	NECAB3
chr20	33100110	33150719	MYH7B, MAP1LC3A
chr20	33290652	33301074	TP53INP2
chr20	33466933	33548685	
chr20	33839610	33917414	TRPC4AP, GDF5, MMP24, GGT7, CPNE1, NFS1
chr20	34651878	34737744	CPNE1, NFS1
chr20	35064315	35122508	
chr20	35887970	36040998	NNAT, MANBAL
chr20	36737140	36802296	TTI1, CTNNBL1, KIAA1755, TGM2
chr20	39744328	39802691	
chr20	39945365	39973640	PLCG1
chr20	42978793	43001116	GDAP1L1
chr20	43015616	43047283	WISP2
chr20	43060069	43094649	GDAP1L1
chr20	43964313	43977507	PABPC1L, SNX21

chr20	44034062	44065730	WFDC2
chr20	45932127	45991840	
chr20	46355492	46411358	
chr20	47200410	47315383	STAU1
chr20	47332704	47422242	CSE1L
chr20	47528689	47566298	
chr20	48290359	48335090	CEBPB
chr20	48380228	48462350	CEBPB
chr20	48524357	48561400	CEBPB
chr20	48720013	48789586	
chr20	48803102	49101366	FAM65C, CEBPB
chr20	49344337	49364919	MOCS3
chr20	50348503	50389782	SALL4
chr20	52194348	52241570	
chr20	52255892	52296764	
chr20	52352178	52423273	
chr20	52474079	52566458	
chr20	55950022	56056724	RAE1
chr20	56104107	56204341	PCK1
chr20	56229753	56295325	BMP7, RAE1, PMEPA1
chr20	56554953	56599579	
chr20	57463250	57471189	NELFCD
chr20	57571301	57591696	NELFCD, SLMO2
chr20	60625295	60645708	MTG2, PSMA7, RBBP8NL, ADRM1, LSM14B, CABLES2, SS18L1
chr20	60709691	60731948	PSMA7
chr20	60783185	60829682	SS18L1
chr20	60866815	60895837	LSM14B

chr20	60912703	60956778	MTG2, PSMA7, MRGBP, TAF4, ADRM1, LSM14B, CABLES2, SS18L1
chr20	60979569	60991115	TCFL5
chr20	61270055	61300727	TCFL5
chr20	61424298	61430672	DIDO1
chr20	61505801	61562562	NKAIN4
chr20	61583036	61629300	OGFR, DIDO1, GID8, SLC17A9, BIRC7, ARFGAP1, YTHDF1
chr20	62150329	62155053	PPDPF
chr20	62164679	62175099	PTK6
chr20	62312233	62336348	PRPF6, C20orf195, HELZ2, SAMD10, YTHDF1, ABHD16B, RP4-583P15.14
chr20	62357798	62383498	SAMD10
chr20	62493840	62498077	ABHD16B
chr20	62578464	62591413	
chr20	62837634	62876976	TPD52L2, DNAJC5, SLC2A4RG, SAMD10, ABHD16B, ZGPAT, UCKL1, PCMTD2, LIME1

chr21	18865047	18900691	
chr21	34774817	34815129	
chr21	36236111	36283282	
chr21	37485227	37581944	DOPEY2
chr21	38737141	38747131	TTC3
chr21	40138244	40204595	PSMG1
chr21	40278501	40306294	ETS2
chr21	40320708	40407095	ETS2, WRB
chr21	41004658	41060316	
chr21	42525684	42552403	
chr21	42866102	42955540	TMPRSS2
chr21	43012823	43106321	C2CD2
chr21	43119523	43150298	RIPK4
chr21	43171945	43206984	C2CD2, UMODL1
chr21	43429153	43433521	
chr21	43718828	43759283	C2CD2
chr21	43870340	43914220	
chr21	43932564	43959840	UMODL1
chr21	44139132	44182145	PDE9A
chr21	44375437	44401511	U2AF1
chr21	44554821	44616839	PKNOX1, RRP1B
chr21	44694400	44783664	RRP1B
chr21	44858977	44873702	AGPAT3
chr21	45116091	45203714	CSTB
chr21	45279081	45324680	PFKL, TRAPPC10
chr21	45397210	45445209	TRAPPC10
chr21	45556368	45643372	RRP1
chr21	45658980	45670752	PFKL, CSTB, TRAPPC10
chr21	45712521	45727180	TRAPPC10
chr21	46255495	46302436	
chr21	46711365	46810661	PCBP3, POFUT2
chr21	46917222	46976311	SLC19A1
chr22	18243372	18270193	

chr22	18312985	18485260	PEX26
chr22	18538170	18575590	PRODH, GGT3P
chr22	19156076	19173402	HIRA
chr22	19829475	19890253	C22orf29
chr22	19940999	20001192	ZDHHC8
chr22	20160883	20234381	ZDHHC8
chr22	20856835	20944344	
chr22	21289918	21337350	AIFM3
chr22	22290909	22316911	SDF2L1
chr22	23524799	23631169	
chr22	23860500	23880998	SLC2A11
chr22	24536186	24571357	SUSD2
chr22	24815198	24842330	SNRPD3
chr22	24938039	24952572	GUCD1
chr22	25338068	25482898	GUCD1
chr22	25505870	25565876	KIAA1671
chr22	25759644	25802946	KIAA1671
chr22	28026276	28083459	TTC28
chr22	29177960	29239632	XBP1
chr22	29399573	29445886	ZNRF3
chr22	30631895	30659464	SLC35E4
chr22	30673897	30714644	SEC14L3
chr22	31440389	31501305	
chr22	31603912	31650966	
chr22	31666929	31694556	
chr22	31735466	31744151	PATZ1
chr22	32008931	32042435	
chr22	35695169	35726874	
chr22	35822544	35885660	MB
chr22	36695421	36870195	
chr22	37406071	37423323	TST
chr22	37558206	37596906	TST
chr22	37662994	37830188	CSF2RB
chr22	37894340	37901230	CARD10
chr22	37955509	37961165	GGA1
chr22	38031888	38040007	SH3BP1
chr22	38122338	38150255	SH3BP1
chr22	38170192	38215249	CARD10
chr22	38493842	38517899	H1F0

chr22	38539997	38666282	
chr22	38678745	38715980	JOSD1
chr22	39085277	39114315	JOSD1
chr22	39128208	39162004	
chr22	39316861	39364502	APOBEC3 B
chr22	39629563	39706904	PDGFB
chr22	39824725	39871784	CBX7, PDGFB, SYNGR1, TAB1, MGAT3
chr22	40380994	40420706	
chr22	41397098	41419171	MKL1
chr22	41791846	41815145	ACO2
chr22	41839294	41844713	TEF
chr22	42217956	42273543	ACO2, TEF
chr22	42299203	42340539	CYP2D7P
chr22	42667765	42797051	CYP2D7P
chr22	43038895	43046055	PACSIN2
chr22	43264661	43439013	
chr22	43513737	43526402	SCUBE1
chr22	46262795	46352015	GTSE1, CELSR1
chr22	46406761	46411260	PPARA
chr22	46445694	46452947	CELSR1
chr22	46458772	46478191	
chr22	46478760	46487142	CELSR1
chr22	46497766	46507175	CELSR1
chr22	46542554	46569987	TTC38, GRAMD4, PKDREJ
chr22	46748627	46787408	CELSR1
chr22	46824524	46862283	
chr22	46937738	47092435	CERK
chr22	47118572	47226600	
chr22	50209411	50261230	
chr22	50319146	50364826	
chr22	50421427	50473904	PLXNB2
chr22	50617874	50646284	

chr22	50711151	50756460	
chr22	50878422	50923490	
chr22	50962309	50971267	
chr3	4753453	4819425	BHLHE40
chr3	5017868	5069286	
chr3	9436892	9444823	
chr3	12432252	12523307	
chr3	12985029	13067745	TMEM40, TSEN2
chr3	13512116	13536382	WNT7A
chr3	14298104	14378584	TMEM43, RP11-434D12.1
chr3	14399898	14514536	TMEM43
chr3	32440189	32475162	CMTM7
chr3	38025920	38036323	ACAA1
chr3	38126526	38196711	
chr3	39166795	39197717	WDR48
chr3	41240003	41247161	
chr3	42052123	42077635	
chr3	42159038	42232502	VIPR1
chr3	42514128	42587318	VIPR1, HIGD1A
chr3	42841612	42871959	VIPR1, HIGD1A
chr3	45097761	45183848	
chr3	45624504	45689506	
chr3	46424499	46496770	PRSS50
chr3	46730797	46756211	TDGF1
chr3	46930314	47040581	
chr3	47436522	47486519	
chr3	48463128	48478174	NCKIPSD
chr3	48505764	48520060	PRKAR2A
chr3	48654866	48675762	IP6K2
chr3	48731379	48785020	SLC25A20
chr3	49054656	49060694	PRKAR2A
chr3	49934784	49945759	
chr3	50160297	50217836	
chr3	50247646	50312226	HYAL3
chr3	50334300	50341598	HYAL1

chr3	50351804	50372211	
chr3	50390536	50495807	RBM5
chr3	50623949	50676454	
chr3	51421594	51438331	
chr3	52057782	52125318	IQCF3
chr3	52318123	52326957	
chr3	53166834	53230973	
chr3	53256227	53310221	
chr3	53854783	53881180	
chr3	57928945	58057559	
chr3	58612512	58692124	FAM3D
chr3	66490553	66558796	
chr3	71074080	71128384	
chr3	71164955	71204485	
chr3	71612437	71634637	
chr3	101553239	101597714	
chr3	107241446	107245110	
chr3	119811908	119817478	COX17
chr3	124670251	124722800	
chr3	126175121	126216648	UROC1
chr3	126679289	126725730	PLXNA1
chr3	126734654	126772223	
chr3	127449108	127543524	
chr3	128722169	128787733	EFCAB12
chr3	129266205	129352965	EFCAB12
chr3	134018149	134102139	
chr3	135905201	135917190	
chr3	141045619	141097069	RNF7
chr3	141112097	141175373	SPSB4
chr3	142314195	142344869	
chr3	149057776	149120135	TM4SF4
chr3	150124943	150129391	
chr3	150441886	150482605	
chr3	151976190	151993419	
chr3	152876999	152881412	
chr3	156787741	156808707	
chr3	156823048	156855928	
chr3	169375755	169387326	PHC3
chr3	169755228	169768436	
chr3	176845712	176942803	

chr3	176997149	177021902	
chr3	177053032	177080948	
chr3	182963086	183004865	
chr3	184062273	184091023	HTR3E
chr3	184264246	184322960	EPHB3
chr3	185244883	185279994	
chr3	187455191	187469615	MASP1
chr3	193530541	193629340	
chr3	193746969	193879826	GP5
chr3	195500894	195660661	MUC20
chr3	195886533	195955303	SLC51A
chr3	196712324	196763744	WDR53
chr3	197181501	197287034	
chr4	673912	733742	ZNF141
chr4	759294	809478	ZNF141
chr4	941817	968153	DGKQ
chr4	984645	1053440	FGFRL1
chr4	1160762	1216551	FGFRL1
chr4	1234500	1244656	FGFRL1
chr4	1323168	1345842	
chr4	1492816	1584306	WHSC1
chr4	1716992	1733011	
chr4	1748180	1818534	HAUS3
chr4	1839929	1910752	LETM1
chr4	2387092	2464880	ZFYVE28
chr4	2742329	2770858	
chr4	2787960	2876249	
chr4	2924111	2945713	
chr4	3287169	3342858	HTT
chr4	6678674	6698695	S100P
chr4	6722091	6782024	TADA2B
chr4	6887698	6972889	TADA2B
chr4	7957954	7988605	ABLIM2
chr4	8077812	8132727	ABLIM2
chr4	8174090	8208427	
chr4	10076448	10126184	ZNF518B
chr4	15002818	15007221	
chr4	25836357	25867877	SEL1L3
chr4	37431895	37492229	PTTG2
chr4	38063934	38084821	PTTG2

chr4	38129750	38165338	
chr4	38663021	38693644	
chr4	40439724	40589443	
chr4	56259521	56265572	
chr4	74960723	74983685	PPBP
chr4	77484965	77548505	
chr4	77584194	77637557	
chr4	90195986	90229685	
chr4	103740245	103751132	
chr4	111114430	111120414	EGF
chr4	111531763	111555575	ENPEP
chr4	124315353	124341272	
chr4	140096935	140110107	
chr4	141056739	141080210	
chr4	141157126	141174915	
chr4	146855605	146860416	
chr4	149348610	149367220	NR3C2
chr4	152233610	152283822	
chr4	153856253	153880544	ARFIP1
chr4	154124664	154197286	
chr4	159692385	159759774	
chr4	160023546	160026111	RAPGEF2
chr4	185202302	185398387	
chr4	187644099	187688085	
chr5	474795	609377	ZDHHC11
chr5	756303	785279	SLC9A3
chr5	979520	1031369	CEP72, SLC12A7, SLC6A3, LPCAT1, ZDHHC11
chr5	1073524	1188157	CLPTM1L, TRIP13, CEP72, LPCAT1, SLC6A18, ZDHHC11
chr5	1470271	1520245	LPCAT1

chr5	6701302	6737323	NSUN2, PAPD7, MED10, SRD5A1
chr5	10284082	10310886	CMBL
chr5	10700031	10762126	
chr5	14142643	14227377	
chr5	16483144	16554639	
chr5	16845602	16933989	
chr5	32298136	32333194	SUB1
chr5	32433949	32447867	SUB1
chr5	40676729	40700663	
chr5	42982801	43011206	
chr5	43034226	43043691	
chr5	43063838	43068028	
chr5	56110126	56113613	
chr5	60614482	60640020	ZSWIM6
chr5	67510489	67581694	
chr5	68787670	68827176	MARVELD 2
chr5	72409581	72432342	TMEM171
chr5	73927127	73940199	ENC1
chr5	75752852	75789307	F2RL1
chr5	79471332	79566020	SERINC5
chr5	90675237	90680688	
chr5	95143290	95179284	
chr5	95983382	96008401	ERAP1
chr5	98108333	98130880	RGMB
chr5	115854281	115911095	SEMA6A
chr5	126333946	126378182	3-Mar
chr5	127414078	127426618	SLC12A2
chr5	130580118	130649740	CDC42SE2
chr5	131581537	131616291	RAD50
chr5	131689653	131724473	SLC22A5, SOWAHA
chr5	131749771	131841995	SOWAHA
chr5	132142089	132177705	SHROOM1
chr5	133328151	133407727	TCF7, VDAC1

chr5	133768336	133803024	C5orf24
chr5	133837574	133866308	C5orf24
chr5	134359884	134384113	PITX1
chr5	134659432	134733027	CXCL14
chr5	139013411	139091824	PSD2
chr5	139104655	139149002	PSD2
chr5	139525353	139574291	CYSTM1
chr5	139595437	139650352	WDR55
chr5	141211083	141263248	KIAA0141
chr5	142160477	142260069	
chr5	148818221	148868910	SLC26A2
chr5	148982530	149060869	ARHGEF37
chr5	149106862	149267616	PDE6A, SLC26A2
chr5	149539500	149568866	TIGD6
chr5	149586118	149597726	HMGXB3
chr5	149826297	149924557	TIGD6
chr5	149941195	150017647	GPX3
chr5	150431504	150530637	SLC36A1
chr5	154133189	154173660	
chr5	159670932	159735006	CCNJL
chr5	167696280	167757224	
chr5	168078410	168111185	PANK3
chr5	170171162	170201193	GABRP
chr5	171530041	171615934	
chr5	172195280	172206972	NEURL1B
chr5	172327125	172346151	ERGIC1
chr5	173191034	173230786	BOD1
chr5	175819506	175855866	KIAA1191
chr5	175956664	176001108	KIAA1191
chr5	176722030	176736996	
chr5	177621106	177679790	PHYKPL
chr5	179229305	179235969	HNRNPH1
chr5	179243293	179248952	HNRNPH1
chr5	180212330	180259493	
chr6	1310123	1336593	FOXQ1
chr6	1588582	1644562	FOXC1
chr6	2210828	2260848	
chr6	2783063	2870724	

chr6	2980675	2992105	
chr6	3052909	3070261	
chr6	3261068	3451653	
chr6	6677371	6760218	
chr6	6789844	6827596	
chr6	7042214	7062450	
chr6	7078402	7279359	
chr6	7531455	7563037	
chr6	10403652	10427002	MAK
chr6	11187519	11237258	
chr6	11253508	11298471	
chr6	11750299	11815661	
chr6	12007326	12027235	
chr6	18260830	18278067	
chr6	20401995	20428889	
chr6	21587001	21598331	SOX4, CDKAL1
chr6	24718942	24722740	CMAHP
chr6	30058788	30124973	ZNRD1
chr6	30691362	30762214	POU5F1
chr6	30843457	30855039	IER3
chr6	31141202	31190866	HLA-B
chr6	31701197	31709066	GPANK1
chr6	31785704	31800301	ATP6V1G2-DDX39B
chr6	31833534	31856917	GPANK1
chr6	32933910	32951015	
chr6	33544245	33549210	
chr6	33586854	33594477	
chr6	33660014	33749771	
chr6	34482430	34578000	SPDEF
chr6	35438934	35474161	
chr6	35691386	35701797	FKBP5
chr6	36079393	36101298	C6orf222
chr6	36277080	36337407	
chr6	36564821	36652372	C6orf222
chr6	36683212	36774267	CDKN1A
chr6	36965965	37029694	PPIL1
chr6	39134334	39219221	KCNK5
chr6	41392111	41438969	UNC5CL

chr6	41455576	41571011	FOXP4
chr6	41650271	41737635	FOXP4
chr6	42056271	42115051	TFEB
chr6	42729722	42758905	
chr6	43214249	43259508	CRIP3
chr6	43635117	43712583	CAPN11
chr6	43726225	43824146	VEGFA
chr6	44002034	44103449	HSP90AB1, VEGFA, SLC35B2, C6orf223
chr6	44184479	44206134	CDC5L
chr6	45914268	45984446	
chr6	52167993	52229585	
chr6	64279147	64286590	
chr6	74223561	74234363	
chr6	89788647	89793074	
chr6	106950828	106973629	
chr6	107137094	107200629	
chr6	108877707	108887837	SESN1
chr6	109611453	109673719	
chr6	109688096	109706678	
chr6	109764627	109788761	FIG4
chr6	111195070	111205022	
chr6	111910301	111954238	TRAF3IP2
chr6	114175529	114194882	
chr6	128799398	128837581	
chr6	135534680	135594210	
chr6	137278355	137291267	
chr6	137344690	137366749	IL20RA
chr6	137536873	137578524	SLC35D3
chr6	138426044	138430485	
chr6	143264915	143269254	ADAT2
chr6	143856647	143876546	
chr6	150489302	150526550	PPP1R14C
chr6	151701476	151714265	RMND1
chr6	157097603	157103712	
chr6	158350244	158390905	ZDHHC14
chr6	158405444	158493053	
chr6	158980595	158996671	OSTCP1

chr6	159049669	159089680	
chr6	159159682	159241230	
chr6	159255811	159292507	
chr6	160100618	160123604	
chr6	167363134	167384572	
chr6	168062964	168162316	TTLL2, KIF25
chr6	168413323	168503148	KIF25
chr6	170574513	170606836	FAM120B
chr7	115873	189300	FAM20C
chr7	298552	336254	PDGFA
chr7	357264	431053	FAM20C, PRKAR1B, PDGFA
chr7	449093	615004	FAM20C, PDGFA
chr7	640661	681000	GPR146, FAM20C, PDGFA
chr7	833693	876996	
chr7	948975	956672	ADAP1
chr7	959479	969152	HEATR2
chr7	983984	990414	HEATR2, SUN1, GET4
chr7	991118	994980	HEATR2
chr7	1013987	1020134	CYP2W1, GPR146, GPER1, INTS1, PRKAR1B, PDGFA, GET4
chr7	1065425	1124042	SUN1
chr7	1184061	1233794	GPR146
chr7	1486311	1514796	TMEM184 A
chr7	1568954	1574711	
chr7	1605706	1611646	
chr7	2477451	2577892	LFNG

chr7	2645525	2776475	BRAT1, TTYH3
chr7	4721270	4794293	MMD2
chr7	5391430	5495983	ZNF815P
chr7	5565783	5654698	EIF2AK1
chr7	6401818	6443481	
chr7	20368046	20394977	
chr7	22369279	22401681	
chr7	25891379	25906154	
chr7	25979727	26008574	
chr7	26191251	26209207	NFE2L3, CBX3
chr7	26222470	26245710	
chr7	27140197	27149295	HOXA1
chr7	27180124	27188836	SKAP2
chr7	27193128	27210499	HOXA3
chr7	27210692	27226589	HOXA2
chr7	27226673	27235671	HOXA5
chr7	27236867	27251265	HOXA5
chr7	27773847	27784785	HIBADH
chr7	29229287	29248948	CHN2
chr7	29601890	29607677	CHN2
chr7	30322996	30327105	
chr7	30932614	30978578	
chr7	36297675	36332564	
chr7	44111154	44152646	
chr7	44650171	44685168	NPC1L1
chr7	47389674	47426372	
chr7	47468138	47651644	PKD1L1
chr7	48124266	48137839	
chr7	51290755	51396780	
chr7	55089642	55164643	
chr7	55572151	55641166	
chr7	66100110	66162918	KCTD7
chr7	70158103	70166365	STX1A
chr7	73113983	73134548	STX1A
chr7	73148980	73158989	MLXIPL
chr7	73180110	73189479	CLDN4
chr7	73235441	73248623	CLDN4
chr7	73385253	73483905	BAZ1B

chr7	73673176	73747387	CLIP2
chr7	73865224	73961408	GTF2IRD1
chr7	74018395	74075950	GTF2IRD1
chr7	75550140	75622127	
chr7	75864295	75949165	POR
chr7	75993211	76034140	UPK3B
chr7	77281637	77331514	
chr7	87835205	87863163	RUNDC3B
chr7	92461754	92466516	PEX1
chr7	97905866	97925528	
chr7	97972452	98050516	
chr7	98805998	98828283	TMEM130
chr7	98969610	98991863	
chr7	99592062	99634221	FAM200A
chr7	99750229	99756770	AGFG2
chr7	100030516	100053867	ZKSCAN1
chr7	100067090	100091823	ZKSCAN1
chr7	100270632	100292722	SLC12A9
chr7	100389991	100435450	TRIP6, VGF, ZAN
chr7	100482826	100510426	CLDN15
chr7	100607523	100631215	AGFG2
chr7	100720585	100766288	TRIP6, VGF, NAT16
chr7	100873719	100896396	CLDN15
chr7	101176441	101288724	VGF, NAT16
chr7	101331053	101391399	MYL10
chr7	101544929	101606786	
chr7	102786993	102794793	POLR2J2
chr7	104581371	104587535	
chr7	104645946	104657826	
chr7	113722871	113728455	
chr7	116411019	116453647	
chr7	117294035	117326750	CTTNBP2
chr7	129588117	129614129	CPA5
chr7	129780377	129795177	TMEM209
chr7	130567798	130646840	
chr7	130667636	130757779	

chr7	138780203	138811181	
chr7	139746413	139763577	
chr7	139900225	139971468	
chr7	140174574	140227308	TBXAS1
chr7	142102038	142134538	PRSS58
chr7	143064821	143092975	
chr7	150064720	150073325	REPIN1
chr7	150099712	150106164	
chr7	150477778	150505955	TMEM176B
chr7	150753888	150760769	AGAP3
chr7	150776486	150781126	
chr7	151493628	151575818	CRYGN, GALNT11, WDR86
chr7	154994800	155029752	
chr7	155076689	155092799	INSIG1
chr7	155579781	155659314	CNPY1, SHH
chr7	155743542	155757869	CNPY1, SHH
chr7	156393561	156419142	
chr7	156791706	156833174	UBE3C
chr7	157065075	157111568	
chr7	158642204	158662120	
chr8	1913890	1926709	
chr8	8263120	8326038	
chr8	8702030	8757987	
chr8	8910478	8963645	
chr8	11274378	11332746	
chr8	11652280	11681536	FAM167A
chr8	11701001	11764237	FAM167A
chr8	17636013	17664074	
chr8	22025073	22086488	SORBS3
chr8	22209326	22250863	PDLIM2
chr8	22405454	22456585	SLC39A14
chr8	22596537	22639491	SORBS3
chr8	22921421	22964018	AC037459.4
chr8	23380882	23428928	

chr8	27189458	27266603	
chr8	28196552	28270528	
chr8	29110995	29154030	
chr8	29197510	29210928	DUSP4
chr8	30356631	30426729	RBPMS
chr8	37400662	37425796	ZNF703
chr8	37446378	37513231	
chr8	37532976	37561473	ERLIN2
chr8	37639460	37773872	
chr8	38234927	38241308	
chr8	38613511	38663892	
chr8	38757313	38816236	
chr8	39897244	39920242	ADAM2
chr8	42744847	42772855	FNTA
chr8	48262147	48294509	CEBPD
chr8	48421653	48455025	SPIDR
chr8	48646118	48651966	
chr8	61811816	61840052	CLVS1
chr8	76314903	76322046	
chr8	81397760	81421513	ZBTB10
chr8	95625352	95666522	
chr8	96077211	96128322	INTS8
chr8	101312661	101349943	ANKRD46
chr8	101955916	101966785	PABPC1
chr8	101997511	102041060	ANKRD46
chr8	102089173	102179732	PABPC1
chr8	102503622	102533609	ZNF706
chr8	103628766	103675494	AZIN1
chr8	103740248	103825586	AZIN1
chr8	110591230	110620476	SYBU
chr8	119086620	119139341	
chr8	121820726	121825333	SNTB1, MTBP
chr8	126415742	126448632	SQLE, NSMCE2, KIAA0196
chr8	126649390	126677556	NSMCE2
chr8	127566250	127571455	MYC, POU5F1B
chr8	128573356	128616312	MYC

chr8	128746466	128755847	MYC, POU5F1B
chr8	134256633	134315934	NDRG1
chr8	134335470	134391007	
chr8	135843061	135845900	
chr8	141516428	141523242	
chr8	141552714	141642515	CHRAC1
chr8	142082599	142174944	
chr8	142203985	142256046	SLC45A4
chr8	142273921	142299957	SLC45A4, DENND3, PTK2, PTP4A3
chr8	142315603	142357428	PTP4A3
chr8	142422272	142429622	PTP4A3
chr8	144089481	144109882	ZC3H3, RHPN1, LY6E, LY6D, ZFP41, TOP1MT
chr8	144342533	144368943	ZC3H3, PYCRL, RHPN1, LY6D, CYP11B2, TIGD5, MAPK15, MAFA, TOP1MT
chr8	144464166	144529325	ZC3H3, TSTA3, RHPN1, CYP11B2, TIGD5, MAPK15, MAFA, TOP1MT
chr8	144653444	144663891	RHPN1

chr8	144813419	144823974	ZC3H3, PYCRL, RHPN1, GRINA, KIAA1875, SCRIB, ZFP41, TOP1MT
chr8	144886670	144908520	ZC3H3
chr8	144938952	144989216	ZC3H3
chr8	145005703	145029567	ZC3H3, KIAA1875
chr8	145043674	145052590	ZC3H3
chr8	145536936	145562791	ARHGAP39
chr8	145577602	145584798	FBXL6
chr8	145637532	145659835	ZNF34
chr8	145724875	145736551	
chr9	2014195	2023271	
chr9	14309511	14323573	
chr9	19229675	19233894	
chr9	27360698	27473689	
chr9	33128522	33168546	AQP3
chr9	33202488	33255307	SPINK4, AQP3
chr9	33287280	33298803	
chr9	33813398	33820021	
chr9	35904158	35925009	HRCT1
chr9	36120900	36166748	
chr9	36290824	36329566	HRCT1
chr9	37911456	38075786	
chr9	71324166	71364433	
chr9	73006949	73036884	KLF9
chr9	74373059	74384783	
chr9	74977205	74981427	
chr9	77686385	77727120	
chr9	80618409	80650066	
chr9	91968129	92116567	CKS2
chr9	92134062	92172043	
chr9	92263575	92302155	GADD45G
chr9	93909435	93975909	

chr9	95492230	95537743	NOL8
chr9	95929205	96033139	
chr9	96211941	96274344	
chr9	96312205	96383980	
chr9	97362246	97412003	
chr9	97765305	97771070	C9orf3
chr9	98248725	98275129	
chr9	99175902	99183433	CDC14B
chr9	100821574	100909505	
chr9	100925141	100958251	GABBR2
chr9	100971057	101016512	
chr9	108009532	108095645	
chr9	110243788	110268173	KLF4
chr9	110348570	110402367	
chr9	112008471	112108750	EPB41L4B
chr9	112943440	112984316	
chr9	113014239	113022612	
chr9	114653204	114671387	PTGR1
chr9	114908851	114943306	
chr9	115067730	115101623	
chr9	116131964	116161408	
chr9	116265295	116396869	
chr9	117124745	117165720	
chr9	123618299	123698895	PHF19
chr9	124023380	124102025	GSN
chr9	124360204	124528771	
chr9	126081274	126144032	ZBTB26
chr9	127018737	127085026	
chr9	129092046	129142270	
chr9	130149295	130165256	SH2D3C, ENG, SLC2A8, ANGPTL2, GARNL3, LRSAM1
chr9	130256294	130351541	
chr9	130587141	130647070	STXBP1
chr9	130664513	130684554	ST6GALNAC6
chr9	130704349	130755491	NAIF1

chr9	130864490	130880714	FAM102A
chr9	130905976	130914424	PTRH1
chr9	131622527	131680147	
chr9	131811455	131850372	FAM73B
chr9	131895420	131909835	PHYHD1
chr9	131924858	131947259	CCBL1
chr9	132094411	132124868	PTGES
chr9	132143494	132269806	PTGES
chr9	132310662	132376115	PTGES
chr9	133223375	133329315	FNBP1
chr9	134103506	134158677	
chr9	134224427	134309073	
chr9	134496283	134555283	
chr9	134571356	134615946	
chr9	135924105	135940404	GBGT1, GTF3C5, ADAMTS13, CELP, CEL, ADAMTSL2
chr9	135966206	136027438	
chr9	136099223	136160808	
chr9	136689130	136859572	DBH
chr9	136914345	136934837	
chr9	137144521	137367806	
chr9	138857212	138909902	SEC16A
chr9	138967415	139090962	CAMSAP1
chr9	139404968	139525089	
chr9	139578763	139591286	CCDC183
chr9	139683661	139691005	MAMDC4
chr9	139739767	139758297	CCDC183
chr9	139774959	139808829	TMEM210
chr9	139836298	139847672	C9orf172
chr9	140087151	140096306	C9orf172
chr9	140122472	140135480	CCDC183
chr9	140169673	140176591	NRARP
chr9	140186990	140203083	GRIN1

chr9	140203344	140212896	MAMDC4, LCN12, C9orf173
chr9	140500027	140510499	NRARP
chrX	12988547	13048990	
chrX	23811271	23841815	
chrX	39812121	39874657	
chrX	39941636	39969370	BCOR
chrX	40000296	40036731	BCOR
chrX	41189758	41195978	
chrX	68047881	68069392	
chrX	70835396	70848211	
chrX	118611219	118647263	
chrX	129193853	129255773	XPNPEP2
chrX	152950541	152975505	TMEM187
chrX	152988183	152993943	BCAP31
chrX	153186855	153196221	ARHGAP4
chrX	153236006	153240516	LAGE3
chrX	153264816	153326419	SRPK3
chrX	153576924	153613104	MECP2

Supplementary Table 2: 2026 super-enhancers and putative gene associations by H3K27ac ChIP-seq signal and RNA-seq mRNA expression correlation, that are supported by Hi-ChIP looping contacts derived from primary CRC tumors.

	'start'	'end'	Genes associated with super-enhancer by Hi-ChIP as well as H3K27ac by mRNA correlation
chr1	839207	879441	
chr1	931032	942910	
chr1	958033	1016775	
chr1	1056306	1073787	RNF223
chr1	1076920	1083409	RNF223
chr1	1092834	1107947	
chr1	1278680	1285456	
chr1	1364974	1379781	TNFRSF4, MIB2, ANKRD65
chr1	1530021	1565186	
chr1	1680282	1729724	PRKCZ
chr1	1820906	1828473	PRKCZ
chr1	1954672	1985568	
chr1	2063722	2092933	
chr1	2157893	2167812	
chr1	2346593	2438208	
chr1	2473058	2481551	

chr1	2485426	2494310	PLCH2
chr1	3228780	3277512	
chr1	3356135	3431583	LRRC47
chr1	6326718	6362599	ESPN
chr1	6379836	6456346	
chr1	6506270	6571895	ESPN
chr1	6639352	6664607	
chr1	8120867	8182464	
chr1	8207520	8285533	PARK7
chr1	8456022	8488406	
chr1	8931738	8960596	CA6
chr1	9221417	9243600	
chr1	9326975	9508779	SPSB1
chr1	9858949	9909954	NMNAT1
chr1	10726087	10897857	MASP2
chr1	11766429	11867807	MAD2L2
chr1	12183318	12269678	MIIP
chr1	12651250	12690803	
chr1	15455486	15557536	CASP9
chr1	15634011	15698833	CASP9
chr1	15735516	15760330	CASP9
chr1	16059949	16070046	CELA2A
chr1	16155997	16164227	
chr1	16465112	16554574	SPATA21
chr1	17019382	17051807	
chr1	17215262	17307660	NBPF1
chr1	17423509	17460118	PADI2, SDHB
chr1	17551677	17582041	SDHB
chr1	17828633	17961597	PADI2
chr1	19233804	19289265	ALDH4A1
chr1	19333613	19427107	ALDH4A1
chr1	19661379	19808674	
chr1	19909319	19947764	NBL1
chr1	19965334	19984929	AKR7L
chr1	20113568	20144846	RNF186
chr1	20796697	20823686	CAMK2N1

chr1	21574800	21669795	
chr1	21932541	22000272	CDC42
chr1	22213631	22265779	CDC42
chr1	22554966	22591190	CELA3B
chr1	23000040	23122584	
chr1	23744351	23780387	FUCA1, TCEA3
chr1	23840199	23920440	ID3
chr1	24117166	24138236	ZNF436
chr1	24426788	24449990	
chr1	25217166	25289408	
chr1	25856096	25901176	C1orf63 (RSRP1)
chr1	26592392	26629600	
chr1	26662449	26705144	PIGV
chr1	26855685	26870766	
chr1	27017777	27034137	
chr1	27155163	27208661	PIGV
chr1	27316744	27360530	
chr1	27424877	27489140	PIGV
chr1	27813161	27927216	XKR8
chr1	28184862	28219220	STX12
chr1	31202847	31297732	SNRNP40
chr1	31885596	31919499	SERINC2
chr1	31974209	32057981	TINAGL1
chr1	32127999	32154793	COL16A1
chr1	32387789	32429778	
chr1	32754249	32819404	BSDC1
chr1	33177547	33241527	KIAA1522
chr1	33342282	33373754	
chr1	33390274	33490740	TMEM54
chr1	33772194	33816350	
chr1	36614482	36653881	
chr1	37936562	37964770	
chr1	39570164	39599196	
chr1	40388079	40456214	
chr1	40494460	40525992	
chr1	41823076	41899496	

chr1	41916513	41984388	
chr1	42611417	42639350	GUCA2B, GUCA2A
chr1	43389469	43447054	C1orf210
chr1	43810360	43839648	C1orf210
chr1	43971999	44054819	SLC6A9
chr1	44448905	44509737	SLC6A9
chr1	44701316	44760395	ERI3
chr1	45097037	45154968	
chr1	45270933	45276806	
chr1	46631993	46660693	
chr1	46766371	46773007	
chr1	47644434	47659486	PDZK1IP1
chr1	47787864	47802416	
chr1	47896527	47918279	FOXD2
chr1	48150864	48195520	
chr1	51761624	51802404	
chr1	53758670	53794340	
chr1	54713922	54891305	TCEANC2
chr1	55024214	55073822	C1orf177 (LEXM)
chr1	55241442	55282938	TTC22, ACOT11, C1orf177 (LEXM)
chr1	59220979	59252104	
chr1	59268562	59291681	JUN
chr1	59314538	59409852	
chr1	61508480	61523688	
chr1	65530848	65534992	
chr1	68154381	68236593	
chr1	68268228	68299628	
chr1	85740062	85774046	
chr1	94031857	94090070	BCAR3
chr1	95239197	95290129	
chr1	109674773	109729623	GNAT2
chr1	110299715	110366988	GNAI3
chr1	110419656	110490886	
chr1	111734321	111748176	

chr1	113230530	113250444	
chr1	113733834	113799569	
chr1	116914777	116952673	ATP1A1
chr1	116996706	117060164	MAB21L3
chr1	120306701	120361067	
chr1	145415373	145457367	
chr1	150530373	150553183	C1orf51 (CIART)
chr1	150568356	150595388	C1orf51 (CIART)
chr1	150942968	150952107	PSMD4
chr1	151466905	151491681	
chr1	151502988	151522989	CELF3
chr1	151907359	151974283	
chr1	152006566	152024217	
chr1	153478933	153511730	S100A6
chr1	153534043	153550588	S100A4
chr1	153571029	153590531	S100A6
chr1	153735777	153778969	
chr1	153915344	153937491	
chr1	154374178	154461718	IL6R
chr1	154942465	154949259	
chr1	154961047	154995970	ZBTB7B
chr1	155009246	155026555	ZBTB7B
chr1	155050463	155059415	EFNA3
chr1	155095442	155113225	GBA
chr1	155161643	155165270	GBA
chr1	155899428	155917420	SLC25A44

chr1	155931349	155979411	C1orf85 (GLMP)
chr1	156046329	156117267	C1orf85 (GLMP)
chr1	156425720	156498221	C1orf85 (GLMP)
chr1	156625898	156664775	RHBG, NES, BCAN, CRABP2, PEAR1
chr1	156711981	156730297	C1orf85 (GLMP)
chr1	159815583	159860425	
chr1	159891304	159896086	
chr1	160981786	160992848	APOA2
chr1	161042898	161060144	USP21
chr1	161358989	161380324	
chr1	164545582	164653369	
chr1	167032473	167105969	GPA33
chr1	167180129	167199590	
chr1	167681244	167727398	
chr1	169013166	169091553	
chr1	180123204	180143474	QSOX1
chr1	181057187	181130317	
chr1	182352118	182367034	TEDDM1
chr1	197740123	197757457	
chr1	199990289	200023200	NR5A2
chr1	200856680	200899634	
chr1	200961014	200998342	
chr1	201260301	201282790	PKP1
chr1	201322392	201383899	PKP1
chr1	201417175	201486897	
chr1	201973808	202104958	ELF3

chr1	202752017	202784691	
chr1	203236601	203316915	FMOD
chr1	204042149	204107886	REN
chr1	204228245	204368586	
chr1	204412490	204482785	
chr1	205216898	205291308	
chr1	205399713	205519696	LEMD1
chr1	205532653	205568500	SLC45A3
chr1	205628438	205650044	ELK4, MFSD4
chr1	206831654	206910943	IL19
chr1	207098037	207154650	
chr1	207174748	207208974	IL19
chr1	207223454	207244245	
chr1	207911155	207929904	
chr1	207979272	208043352	
chr1	208274061	208359483	
chr1	208373939	208424650	PLXNA2
chr1	209769236	209833030	
chr1	211502176	211527688	
chr1	211687472	211723080	
chr1	211776506	211828545	TRAF5
chr1	212768614	212783470	
chr1	223892515	223938872	
chr1	225596010	225668822	DNAH14
chr1	226029826	226110273	
chr1	226288057	226325426	TMEM63A

chr1	226815255	226927099	
chr1	228072907	228141375	ZNF678
chr1	228246419	228278681	
chr1	228326981	228331088	
chr1	228349132	228354871	RNF187
chr1	228940289	229004627	RHOU
chr1	229228138	229309977	
chr1	229356331	229390079	
chr1	230209480	230297332	
chr1	230864436	230904943	
chr1	231531350	231559064	
chr1	232714463	232771391	
chr1	233743752	233761980	
chr1	234657523	234695975	
chr1	234724833	234760650	
chr1	234834348	234888304	
chr1	234951286	235069151	
chr1	235088748	235158297	
chr1	244209587	244240054	
chr1	244995993	245002365	C1orf101 (CATSPERE)
chr1	245115380	245136153	
chr1	249137064	249168535	
chr10	634473	710051	
chr10	972598	1001352	
chr10	3777459	3830408	
chr10	3846361	3854638	
chr10	3867604	3950023	
chr10	5506961	5706245	ASB13

chr10	11704540	11759069	
chr10	14564566	14667661	
chr10	15331568	15397491	FAM171A1
chr10	21455548	21464438	
chr10	24737364	24784244	
chr10	25004669	25021540	
chr10	27519398	27532774	
chr10	30706423	30726113	
chr10	32329666	32349392	
chr10	32383993	32479292	
chr10	32621022	32674058	
chr10	33227489	33272835	
chr10	43832131	43917416	
chr10	46990958	47058393	NPY4R
chr10	70976222	70993459	CCAR1
chr10	71081085	71149948	VPS26A
chr10	71164584	71267921	
chr10	71595936	71678030	
chr10	71885733	71903754	EIF4EBP2
chr10	72969762	73056436	UNC5B
chr10	73356961	73422741	UNC5B
chr10	73444381	73542810	
chr10	74002542	74100641	ANAPC16
chr10	74420932	74463640	
chr10	75603323	75677591	
chr10	76946014	76973090	VDAC2
chr10	76985609	77056330	ZNF503
chr10	77154927	77169743	ZNF503
chr10	79623645	79682923	
chr10	80710759	80737653	ZMIZ1
chr10	80804683	80922165	
chr10	80981446	81119139	EIF5AL1
chr10	81135308	81202484	SFTPA1
chr10	81895869	81968780	DYDC1
chr10	82187931	82269537	
chr10	85918778	85965078	GHITM, C10orf99

chr10	88424408	88476592	
chr10	88727013	88732486	
chr10	88851230	88865926	
chr10	89621087	89629308	CFL1P1
chr10	90639024	90664381	
chr10	95172495	95236263	
chr10	95301521	95351810	FFAR4
chr10	96990557	97074536	
chr10	98337456	98394081	
chr10	99086316	99097482	
chr10	99457428	99486837	
chr10	102122439	102137454	
chr10	102755594	102779103	PAX2
chr10	102796517	102832180	
chr10	103872182	103881363	FGF8
chr10	104158359	104183191	ARL3
chr10	104353343	104438983	
chr10	105302757	105380116	CNNM2
chr10	105395066	105612788	NEURL1
chr10	105646533	105683374	NEURL1
chr10	106059240	106110982	GSTO1
chr10	111753970	111773182	
chr10	111962979	111981869	MXI1
chr10	112113616	112191012	
chr10	112562258	112639452	PDCD4
chr10	114090057	114173118	
chr10	114705275	114722724	
chr10	114760323	114857045	
chr10	115704372	115722023	

chr10	116254043	116284052	
chr10	119102004	119135732	
chr10	121011545	121080495	BAG3, NANOS1, GRK5
chr10	123686351	123696391	NSMCE4A
chr10	123773041	123824543	
chr10	123860764	123910041	ATE1
chr10	126275072	126437053	
chr10	126687348	126851492	
chr10	129670011	129721503	
chr10	134197375	134245029	
chr10	134257773	134301784	
chr10	134384253	134426321	LRRC27
chr10	135070841	135093527	ZNF511
chr10	135156522	135179866	CALY, MTG1, PRAP1
chr11	199803	210282	
chr11	312970	318010	ANO9, IFITM1
chr11	390974	418670	ANO9
chr11	438101	448828	DEAF1, TMEM80, CEND1, ANO9
chr11	487117	519196	
chr11	557515	564610	ANO9
chr11	606195	622242	
chr11	701921	711865	ANO9
chr11	818110	822789	ANO9
chr11	823355	835562	CEND1
chr11	1027798	1158380	
chr11	1214325	1285146	
chr11	1525034	1597870	TOLLIP
chr11	1772068	1825281	ASCL2
chr11	1850339	1864639	ASCL2
chr11	2007426	2018993	KCNQ1, CD81, ASCL2
chr11	2222838	2227981	ASCL2

chr11	2228439	2233334	ASCL2
chr11	2233511	2240175	ASCL2
chr11	2277708	2293051	ASCL2
chr11	2531969	2557416	ASCL2
chr11	2745510	2769952	ASCL2
chr11	2782893	2865875	CD81
chr11	2922397	2926733	TRPM5
chr11	2930168	2936397	OSBPL5
chr11	2947884	2955533	SLC22A18
chr11	3143708	3192459	ZNF195
chr11	7593139	7644873	
chr11	8708696	8755650	
chr11	8768419	8836787	
chr11	10306635	10374218	
chr11	12065965	12303424	
chr11	16921358	16947833	
chr11	16967632	17032859	KCNJ11
chr11	27485463	27494502	
chr11	34066224	34077172	
chr11	34171953	34296972	
chr11	34605603	34627752	
chr11	34641466	34676856	
chr11	35159997	35201316	
chr11	36366405	36403697	
chr11	44589671	44656439	CD82
chr11	44952460	45004436	TP53I11
chr11	45167037	45204540	PRDM11, TSPAN18
chr11	46258923	46340902	ATG13
chr11	47395923	47437968	ATG13
chr11	48004523	48088987	
chr11	57037148	57094593	ZDHHC5
chr11	57528674	57569477	
chr11	58939074	58975027	DTX4, GLYATL1
chr11	59521144	59568809	
chr11	60885373	60933207	ZP1
chr11	61298131	61396818	

chr11	61460952	61469382	CPSF7
chr11	61519574	61526232	MYRF
chr11	61721960	61749932	FADS3
chr11	62303222	62328979	HNRNPUL2
chr11	63529313	63537924	C11orf84 (SPINDOC)
chr11	63602327	63659507	
chr11	63682671	63690515	RTN3
chr11	64004653	64012011	MARK2
chr11	64084259	64091917	MARK2
chr11	64654508	64663543	SNX15
chr11	65043034	65085130	DPF2, CDC42EP2, TM7SF2, LTBP3, MAP4K2, EHBP1L1, SIPA1
chr11	65146042	65153316	MAP3K11
chr11	65182698	65196358	MAP3K11
chr11	65235731	65278300	KCNK7
chr11	65336369	65346081	SYVN1
chr11	65541984	65595884	DPF2
chr11	65609879	65638159	OVOL1
chr11	66621798	66651079	
chr11	66799161	66863232	PITPNM1
chr11	66880913	66897995	RBM14
chr11	67000685	67058673	CABP4
chr11	67118214	67125134	
chr11	67138085	67142641	CABP4
chr11	67231049	67237376	CABP4
chr11	67253402	67276815	PITPNM1
chr11	67396050	67415560	CABP4
chr11	67775272	67783134	
chr11	67803411	67811072	
chr11	67857262	67933103	CHKA
chr11	67963386	67984625	
chr11	68063591	68237978	
chr11	68577616	68638315	CPT1A, TPCN2
chr11	68861560	68905716	IGHMBP2, TPCN2, MRGPRF
chr11	69036308	69080715	IGHMBP2, MRGPRF, TPCN2
chr11	69142617	69324571	CCND1, MRGPRF, TPCN2, FGF19
chr11	69451044	69483535	CCND1

chr11	69777158	69847417	CTTN, PPFIA1, FGF19, FADD
chr11	70961547	71022977	
chr11	71932563	71952314	
chr11	72414607	72501733	ATG16L2
chr11	72518642	72544162	
chr11	72884579	72953894	
chr11	73076627	73104220	
chr11	73668076	73731342	
chr11	74854195	74915339	SLCO2B1
chr11	75012037	75064571	MOGAT2
chr11	75176812	75248626	GDPD5
chr11	75264295	75306314	SERPINH1
chr11	76447841	76517502	TSKU
chr11	76773786	76787721	
chr11	76796563	76803020	TSKU
chr11	77170698	77186470	
chr11	94258920	94283685	
chr11	117052240	117100620	APOA5
chr11	117679662	117714818	CEP164, FXYD6, SCN2B
chr11	117944260	117963999	
chr11	118480089	118513590	MPZL3
chr11	118659132	118663498	
chr11	118740466	118811184	DDX6
chr11	119227146	119247355	USP2, PDZD3, C2CD2L
chr11	119533622	119620178	
chr11	119980432	120018196	TRIM29
chr11	120039014	120105066	OAF
chr11	121315953	121353712	
chr11	124615370	124639616	
chr11	126180616	126350742	ST3GAL4
chr11	128317351	128389642	

chr11	129856355	129874646	
chr11	130011761	130090730	APLP2
chr12	607333	620505	SLC6A12
chr12	633556	685948	SLC6A12
chr12	718228	756396	
chr12	1682523	1718555	
chr12	1764325	1791236	LRTM2
chr12	1904657	1956059	
chr12	2210461	2280312	DCP1B, CACNA1C, LRTM2
chr12	3813953	3867210	
chr12	4378093	4417824	
chr12	6267425	6351305	PLEKHG6
chr12	6418899	6423651	LPAR5
chr12	6441984	6453122	PLEKHG6
chr12	6468473	6473930	PLEKHG6
chr12	6477590	6487215	SCNN1A
chr12	6641233	6661870	
chr12	6712215	6724398	
chr12	7033601	7038885	
chr12	7063241	7074431	
chr12	11801391	11839716	
chr12	12669886	12716384	
chr12	12856536	12911664	
chr12	12928123	12963912	GPRC5D
chr12	13023563	13064122	GPRC5A
chr12	13515032	13544386	
chr12	14338390	14374087	
chr12	26253869	26288438	
chr12	31470814	31479941	
chr12	32543708	32556907	FGD4
chr12	33022694	33073384	
chr12	46120396	46129994	
chr12	46649613	46664328	SLC38A1
chr12	46761555	46797433	RPAP3
chr12	48193261	48233528	RPAP3

chr12	48252290	48307206	VDR
chr12	48332134	48377560	ASB8
chr12	49147496	49219129	ADCY6
chr12	49450777	49455187	
chr12	50260667	50295089	AQP6
chr12	50632112	50672288	
chr12	50901291	50962394	
chr12	51656594	51670845	
chr12	51779978	51802516	GALNT6
chr12	52205241	52265790	
chr12	52403001	52481069	
chr12	52536284	52564415	KRT84
chr12	53253487	53322649	MFSD5
chr12	53335421	53403264	MFSD5
chr12	53437709	53467279	CSAD
chr12	53609386	53636317	ESPL1
chr12	53717912	53784473	MFSD5
chr12	56117619	56139964	
chr12	56320233	56334550	RDH5
chr12	56472617	56481929	RDH5
chr12	56536023	56559443	RDH5
chr12	57016619	57034040	
chr12	57063053	57094665	
chr12	57479842	57500211	R3HDM2
chr12	57519739	57575138	KIF5A
chr12	57910582	57936584	
chr12	58225534	58293317	R3HDM2
chr12	71546847	71560616	
chr12	89727142	89785423	
chr12	102084835	102107721	
chr12	106620916	106644657	TCP11L2
chr12	106667456	106705140	TCP11L2
chr12	109116029	109129394	
chr12	109871572	109906385	

chr12	110662371	110708258	
chr12	110994563	111043503	
chr12	111827599	111888807	ACAD10
chr12	112179011	112230760	TMEM116
chr12	112243969	112289496	TMEM116
chr12	113338786	113379601	
chr12	113554756	113592779	RASAL1
chr12	113631740	113709983	
chr12	115093425	115142393	TBX3
chr12	117463599	117557001	TESC, RNFT2
chr12	118489390	118502783	
chr12	120105481	120132791	PRKAB1
chr12	120636380	120680464	
chr12	121096466	121131147	C12orf43
chr12	121392155	121423524	SPPL3
chr12	121650947	121685811	P2RX4
chr12	122223345	122245252	BCL7A
chr12	122467247	122530502	DIABLO
chr12	122580629	122625739	DIABLO
chr12	123310119	123406755	CCDC62
chr12	123543736	123636760	CCDC62
chr12	124836999	124951266	
chr12	124964249	125050163	DHX37
chr12	125066524	125263109	SCARB1
chr12	125299360	125428074	SCARB1
chr12	132951183	133031675	EP400

chr12	133048434	133101921	
chr12	133396203	133415898	PXMP2
chr13	20691989	20703211	
chr13	27522316	27597035	USP12
chr13	28016432	28030077	GSX1
chr13	28479626	28498912	
chr13	28526733	28555860	
chr13	30944754	30996971	
chr13	31354551	31446668	HSPH1, USPL1
chr13	41237892	41242018	WBP4
chr13	41537775	41597025	
chr13	42600548	42616099	AKAP11
chr13	50695590	50705124	KPNA3
chr13	72437884	72449334	
chr13	73611749	73666270	
chr13	74703519	74710680	
chr13	80910634	80917643	
chr13	97873693	97931632	
chr13	99083225	99245634	
chr13	99841992	99885096	SLC15A1
chr13	100608044	100649785	PCCA
chr13	106736061	106757196	
chr13	107172698	107188987	
chr13	110419448	110455803	IRS2
chr13	111155382	111234492	
chr13	111252364	111299825	
chr13	111559728	111581992	
chr13	113324635	113382209	PCID2, ATP11A, TUBGCP3, MCF2L, F10
chr13	113526714	113566003	LAMP1, ATP11A, TUBGCP3, MCF2L, F10, PCID2, CUL4A
chr13	113607947	113677177	MCF2L, F10, PCID2, CUL4A, DCUN1D2, LAMP1
chr13	114427335	114521485	DCUN1D2, TMEM255B, LAMP1, TFDP1

chr13	114534188	114584381	CDC16, DCUN1D2, UPF3A
chr13	114739908	114932567	CDC16, UPF3A, TMEM255B, CHAMP1
chr14	21131506	21158048	TMEM253
chr14	21564681	21573947	
chr14	23006813	23040130	
chr14	24885885	24909709	
chr14	34482763	34533357	
chr14	35340799	35344775	
chr14	35799919	35875246	NFKBIA
chr14	38052216	38072548	
chr14	50090494	50110091	
chr14	50233313	50238684	
chr14	50328116	50387669	KLHDC2
chr14	50412382	50490957	
chr14	50499354	50535797	
chr14	54409834	54430681	BMP4
chr14	55031455	55035147	
chr14	55543370	55601049	LGALS3
chr14	55744059	55825561	LGALS3
chr14	61739169	61749050	
chr14	61927818	62037651	SYT16
chr14	64955758	64976110	
chr14	65085747	65233880	HSPA2
chr14	65395006	65440194	HSPA2
chr14	65689140	65772058	CHURC1-FNTB
chr14	67864295	67955825	TMEM229B
chr14	68968180	68996099	ACTN1
chr14	69013307	69079250	
chr14	69225159	69291477	
chr14	69380265	69447558	
chr14	70072262	70194859	
chr14	71786661	71807764	
chr14	74185477	74274163	ENTPD5
chr14	74427852	74495599	ENTPD5
chr14	75401472	75454960	PGF

chr14	75599429	75674050	FOS
chr14	75717115	75782305	FOS
chr14	75904523	75944066	FOS
chr14	77412867	77432371	
chr14	77461667	77568858	
chr14	89879722	89896100	FOXN3
chr14	90847362	90851754	
chr14	90966987	91023449	TTC7B
chr14	91817703	91881574	RPS6KA5
chr14	92958237	93040449	
chr14	93468082	93583489	TMEM251
chr14	94848020	94862793	SERPINA1
chr14	95696947	95828465	CLMN
chr14	95906301	95990836	GLRX5
chr14	99696766	99741599	BCL11B
chr14	99983921	100089112	SETD3
chr14	100569955	100660583	
chr14	100704039	100720064	
chr14	100848393	100910735	
chr14	100989114	101036015	
chr14	101971588	102025141	
chr14	102171937	102201097	
chr14	102547539	102564353	
chr14	102930302	103061763	
chr14	103227150	103294455	
chr14	103367432	103415466	AMN
chr14	103978637	104018685	ZFYVE21
chr14	104158621	104197021	ZFYVE21
chr14	104320927	104376746	
chr14	104546791	104586533	

chr14	104686749	104713574	
chr14	105116452	105174971	
chr14	105309426	105362522	
chr14	105423465	105449860	C14orf79
chr14	105498203	105561011	C14orf79
chr14	105621866	105674110	JAG2
chr14	105758950	105830896	
chr14	105944150	105959031	
chr14	107250879	107259790	
chr15	29962135	29982408	FAM189A1
chr15	31493186	31528909	
chr15	31546526	31573312	
chr15	31616912	31698718	
chr15	31725421	31791946	
chr15	34651240	34660801	
chr15	39876202	39938272	FSIP1
chr15	40328510	40401905	BMF, CCDC9B
chr15	40634403	40643032	PHGR1
chr15	40727561	40759825	IVD
chr15	41054990	41088148	
chr15	41135119	41140730	CHP1
chr15	41765875	41806408	ITPKA
chr15	42325351	42447162	
chr15	43414495	43430084	
chr15	44998105	45023192	
chr15	45454839	45480972	SHF
chr15	45716708	45749759	SLC30A4
chr15	45923497	45953467	SLC30A4
chr15	57576544	57636050	MYZAP
chr15	57829356	57911847	
chr15	59535671	59672981	
chr15	59821634	59863374	GCNT3

chr15	60654602	60701728	
chr15	63329419	63385012	
chr15	63637454	63689603	CA12
chr15	63729366	63814439	APH1B
chr15	64257574	64322400	DAPK2
chr15	65127549	65191429	
chr15	65372000	65393928	SLC51B
chr15	66997057	67073974	SMAD6
chr15	67315989	67483410	
chr15	68479899	68503417	FEM1B
chr15	69104516	69114591	
chr15	69583168	69623396	PAQR5
chr15	70381141	70406522	
chr15	70767028	70825700	
chr15	72514662	72530599	
chr15	74666150	74735360	PML
chr15	74890326	74911842	CSK
chr15	75470043	75516959	SIN3A
chr15	75938741	75996895	UBE2Q2
chr15	77280658	77331255	RCN2
chr15	78326848	78398281	
chr15	80951964	81004924	
chr15	85276709	85307822	SLC28A1
chr15	85359945	85411716	SLC28A1
chr15	86293117	86344492	
chr15	89158014	89193681	
chr15	89631585	89694599	
chr15	90540912	90650466	AP3S2
chr15	90752587	90759016	AP3S2, GDPGP1, ANPEP, C15orf38 (ARPIN), C15orf38-AP3S2 (ARPIN-AP3S2)
chr15	91356656	91420850	
chr15	93157113	93211680	
chr15	93344399	93400683	
chr15	93437871	93469422	ST8SIA2
chr15	96864752	96904833	
chr15	99942642	99995817	

chr16	118069	136722	RHBDF1, NPRL3
chr16	356946	415139	
chr16	428699	437016	WFIKKN1
chr16	573964	582164	WFIKKN1
chr16	582919	587823	WFIKKN1
chr16	636097	645846	RAB40C
chr16	690358	701750	GNG13
chr16	724323	731964	WFIKKN1
chr16	845690	892971	UBE2I
chr16	1308919	1363314	UBE2I, SSTR5
chr16	1457757	1491537	UBE2I
chr16	1942780	2000825	RPL3L
chr16	2027583	2037544	MEIOB
chr16	2131635	2144296	
chr16	2205619	2214366	PDPK1
chr16	2561938	2567661	
chr16	2805025	2847185	
chr16	3054054	3061124	
chr16	3069834	3074514	PKMYT1
chr16	3193126	3248938	ZNF200
chr16	3686587	3707943	DNASE1
chr16	4335195	4398116	TFAP4
chr16	4644232	4669625	GLYR1
chr16	4684153	4753034	CDIP1
chr16	4963908	4998478	PPL
chr16	8943472	8986349	METTL22
chr16	9002905	9063032	
chr16	10641327	10723661	
chr16	11290981	11360138	SOCS1
chr16	11651637	11735371	PRM2
chr16	11816017	11848057	
chr16	11876173	11893946	
chr16	14728240	14767544	NTAN1
chr16	17352730	17375255	
chr16	18999769	19019920	
chr16	19119247	19148441	

chr16	19394248	19449678	
chr16	22199573	22251193	PDZD9
chr16	22364488	22386854	
chr16	23302955	23377994	
chr16	23698811	23724946	
chr16	27200578	27256196	IL4R
chr16	27324440	27342571	
chr16	28488682	28512371	SULT1A1, SULT1A2
chr16	29815835	29822485	
chr16	30123335	30135353	MAPK3
chr16	30667396	30681967	
chr16	30816439	30856714	KAT8
chr16	31137030	31147852	C16orf58 (RUSF1)
chr16	31149383	31154958	C16orf58 (RUSF1)
chr16	31487207	31500133	C16orf58 (RUSF1)
chr16	48635742	48662738	
chr16	49886677	49893398	
chr16	50278321	50347909	
chr16	50572354	50667579	NKD1
chr16	50698862	50754682	
chr16	52578178	52613011	
chr16	53064422	53096863	
chr16	53119101	53136836	
chr16	53163214	53168804	
chr16	56945242	56968944	MT1JP
chr16	56998302	57076712	
chr16	57118390	57188393	RSPRY1
chr16	57294197	57351317	HERPUD1
chr16	57503872	57521139	RSPRY1
chr16	57611459	57682895	KIFC3
chr16	57791408	57861419	CSNK2A2, KIFC3
chr16	58055117	58083295	
chr16	66542493	66561415	CKLF
chr16	67264501	67281550	ATP6V0D1
chr16	67423853	67447927	LRRC36
chr16	67460809	67504574	ATP6V0D1, HSD11B2

chr16	68103403	68122765	NRN1L
chr16	68260190	68274411	SMPD3
chr16	68290935	68324768	
chr16	68384973	68432029	SMPD3
chr16	68444955	68469934	
chr16	68730192	68828253	SMPD3
chr16	69596240	69602314	TANGO6
chr16	69856684	69897011	
chr16	70413518	70485050	IL34
chr16	70713976	70803361	VAC14
chr16	71924300	71932810	PHLPP2
chr16	73066783	73106221	ZFHX3
chr16	74747247	74815204	LDHD
chr16	75085989	75147825	
chr16	75258266	75302516	TMEM170A
chr16	75540824	75584967	CHST5, CHST6, LDHD
chr16	81299560	81312418	ATMIN
chr16	81465362	81687328	
chr16	81718415	81789855	
chr16	84362904	84424162	ATP2C2
chr16	85013578	85303424	GSE1
chr16	85317614	85431400	GSE1
chr16	85449100	85526770	GSE1
chr16	85546638	85715065	
chr16	85925110	86032329	
chr16	87394373	87423188	
chr16	87489743	87552690	ZCCHC14
chr16	87860460	87932015	SLC7A5, ZCCHC14
chr16	87978709	88000830	
chr16	88443224	88621560	ZC3H18
chr16	88681562	88714823	
chr16	88971595	89008487	
chr16	89114297	89190040	
chr16	89208436	89277923	ANKRD11
chr16	89360311	89573034	
chr16	89630327	89708513	DPEP1, MC1R, GAS8, SPATA33

chr16	89786044	89790774	
chr16	89887868	89930922	CDK10
chr17	170735	207830	GLOD4
chr17	964335	1050931	PITPNA
chr17	1101108	1135946	PITPNA
chr17	1386109	1397462	ABR
chr17	1411167	1421098	
chr17	1449335	1525362	YWHAE
chr17	1543204	1555138	ABR
chr17	1633308	1691678	HIC1
chr17	1956802	2005409	
chr17	2286152	2316398	
chr17	2607521	2634988	PAFAH1B1
chr17	2658328	2718439	
chr17	3763128	3839913	ATP2A3
chr17	3856496	3892930	ATP2A3
chr17	4033536	4048892	ATP2A3
chr17	4377104	4447820	MYBBP1A
chr17	4847470	4854915	ZFP3
chr17	6448696	6478672	PITPNM3
chr17	6915193	6919266	
chr17	7157918	7195844	YBX2
chr17	7455758	7472180	EFNB3
chr17	7736374	7755771	SLC25A35
chr17	8053741	8059831	KRBA2
chr17	10676053	10719009	TMEM220
chr17	15847554	15895545	TTC19
chr17	16188246	16209563	
chr17	16346060	16393603	TTC19
chr17	16884214	17030840	ZNF287
chr17	17365141	17483447	COPS3
chr17	17566759	17696758	DRG, PEMT, COPS3, GID4
chr17	17710689	17763585	GID4
chr17	17795500	17864058	
chr17	18864569	18923164	
chr17	19268613	19306669	

chr17	21147218	21201645	
chr17	25641314	25681602	
chr17	25782737	25977397	
chr17	26120397	26151772	NOS2
chr17	26797440	26877241	NLK
chr17	27056274	27088768	FAM222B
chr17	27275350	27323531	TLCD1
chr17	27451350	27508908	ERAL1
chr17	27892391	27896886	
chr17	27913646	27922097	
chr17	29776133	29841499	SUZ12
chr17	29868692	29926601	
chr17	31094931	31201094	TMEM98
chr17	34090986	34129390	
chr17	34947717	34965729	
chr17	36022324	36112743	
chr17	36570831	36623254	RPL23
chr17	36713468	36774104	
chr17	36857346	36863504	
chr17	37024425	37079151	
chr17	37779956	37790775	ERBB2
chr17	37827160	37837462	FBXL20
chr17	37851220	37876195	RAPGEFL1
chr17	37892232	37913020	THRA
chr17	38214419	38291144	THRA
chr17	38327873	38351879	RAPGEFL1
chr17	38462778	38514141	LRRC3C
chr17	38598504	38717200	TNS4
chr17	39682460	39687590	KRT9
chr17	39773577	39847775	KRT9
chr17	39911008	39965227	RAB5C
chr17	40660962	40711847	RAMP2
chr17	40819122	40832643	G6PC
chr17	41437009	41447440	
chr17	41604728	41670017	VAT1
chr17	42142360	42196719	SOST

chr17	42267937	42300185	C17orf105 (CFAP97D1)
chr17	43203803	43210810	ARHGAP27, PLCD3
chr17	43246646	43251926	DBF4B
chr17	43298334	43345181	
chr17	43361630	43419461	PLCD3
chr17	43448737	43525469	PLCD3
chr17	44257573	44291311	
chr17	45886253	45900391	PRR15L
chr17	45919982	45966551	CDK5RAP3, SP6
chr17	46021753	46046385	SP6
chr17	46652361	46661153	
chr17	46678428	46685618	
chr17	46685827	46707205	SKAP1
chr17	46707317	46725544	SKAP1
chr17	46795579	46807735	PRAC1, HOXB13
chr17	47928174	48041573	DLX3, DLX4
chr17	48104548	48157833	PDK2
chr17	48176137	48195174	PDK2
chr17	48223171	48230448	DLX4
chr17	48334907	48368245	TMEM92
chr17	48609481	48629133	PDK2
chr17	48696124	48781740	SPATA20
chr17	48909993	48947019	SPATA20, RSAD1
chr17	48960368	49034364	SPATA20
chr17	49195287	49210944	
chr17	54958902	54994593	
chr17	55161780	55198568	
chr17	55478973	55541126	MRPS23
chr17	55928476	56033391	RNF43
chr17	56407935	56417941	RNF43
chr17	57442508	57459901	
chr17	57904169	57934071	
chr17	59467114	59503640	INTS2, BRIP1
chr17	59520418	59574250	TBX4
chr17	61505427	61530049	TCAM1P
chr17	62763527	62791069	POLG2

chr17	63510723	63591127	
chr17	70106480	70124078	
chr17	70367242	70537149	
chr17	70556570	70632645	
chr17	71263731	71313768	
chr17	72368428	72480556	
chr17	72736969	72766975	
chr17	72937453	72988378	
chr17	73069985	73125224	SLC16A5
chr17	73519791	73524830	LLGL2
chr17	73529524	73542716	ACOX1
chr17	73542870	73547822	ACOX1
chr17	73568797	73577073	LLGL2, ACOX1, SMIM5, SMIM6, CDK3, MYO15B
chr17	73604926	73617522	ACOX1, CDK3, MYO15B
chr17	73634586	73644264	ACOX1
chr17	73674822	73751967	
chr17	73765374	73783587	UBALD2
chr17	73805462	73845266	
chr17	73865675	73875420	
chr17	73990524	74001796	ACOX1, CDK3
chr17	74233734	74276263	ACOX1
chr17	74485442	74490614	
chr17	74625305	74713030	
chr17	75093997	75143308	
chr17	75275552	75486474	
chr17	76120080	76130605	
chr17	76164033	76173337	
chr17	76309704	76362807	
chr17	76377008	76417713	
chr17	76988200	76995813	
chr17	77064697	77166637	USP36
chr17	77763241	77790058	CBX8
chr17	77803067	77841269	CBX8, CBX4
chr17	77881082	77925857	CARD14, TBC1D16, CBX8, CBX4
chr17	77948497	78005576	CBX8, CBX2, CBX4, TBC1D16
chr17	78226455	78263038	CBX8

chr17	78419066	78486066	TBC1D16
chr17	78932703	78978711	
chr17	78991762	79075666	BAIAP2
chr17	79301713	79306416	BAIAP2, FAAP100 (FAAP100), TSPAN10, TMEM105, HGS, OXLD1
chr17	79310370	79321005	ARL16
chr17	79369621	79374838	SIRT7
chr17	79435289	79505004	SIRT7
chr17	79667522	79709019	
chr17	79763639	79788092	CCDC137
chr17	79801055	79830999	
chr17	79948174	79980814	ASPSCR1, NOTUM
chr17	80051877	80066204	MYADML2
chr17	80151157	80206536	
chr17	80223855	80233218	
chr17	80249989	80257778	
chr17	80284309	80293682	SIRT7
chr17	80482483	80562717	
chr17	80585291	80669969	RAB40B
chr17	80793052	80880974	TBCD, ZNF750
chr17	81005827	81069196	
chr18	2635791	2659462	
chr18	3008916	3088358	
chr18	3256936	3266371	
chr18	3446204	3467197	
chr18	3583274	3626174	
chr18	11972481	12059708	IMPA2, CHMP1B, MPPE1
chr18	19597999	19642477	
chr18	19741865	19781074	
chr18	28676993	28682939	
chr18	29064876	29094028	
chr18	45550577	45704288	
chr18	46276601	46378172	
chr18	46443881	46487731	SMAD7
chr18	46500457	46527954	
chr18	47337489	47345951	C18orf32

chr18	47372132	47392148	ACAA2
chr18	55395874	55467803	NEDD4L
chr18	55842452	55915151	NEDD4L
chr18	59988044	60025459	KIAA1468 (RELCH)
chr18	60189376	60194365	
chr18	60381757	60385355	KIAA1468 (RELCH)
chr18	67953211	67961246	
chr18	72904616	72943333	
chr18	74202614	74208480	
chr18	74764624	74842527	
chr18	77438452	77483985	TXNL4A
chr18	77694286	77736594	HSBP1L1
chr19	282076	296359	ODF3L2
chr19	509538	559375	GRIN3B
chr19	656677	664392	PALM
chr19	718453	803563	STK11
chr19	923608	929931	FSTL3, PALM, ARID3A
chr19	950367	958117	FSTL3, CIRBP, APC2, ARID3A, SHC2, R3HDM4
chr19	1025797	1029184	GRIN3B
chr19	1129240	1134649	WDR18
chr19	1247317	1256284	
chr19	1256513	1273670	
chr19	1282741	1294598	REEP6
chr19	1406202	1413839	CNN2
chr19	1744843	1808368	RPS15
chr19	1851612	1865911	
chr19	1929069	1948219	AMH
chr19	2021565	2096704	
chr19	2153810	2192227	
chr19	2232428	2238391	MKNK2
chr19	2268933	2274704	AMH
chr19	2326726	2361944	BTBD2
chr19	2476152	2681557	SF3A2
chr19	3093622	3109716	GNA11
chr19	3171695	3192696	NCLN
chr19	3334567	3405371	

chr19	3428287	3484190	TBXA2R, APBA3
chr19	3547728	3578659	
chr19	3687575	3727076	CREB3L3
chr19	4034556	4150644	MATK
chr19	4327469	4346051	CREB3L3
chr19	4361007	4395075	
chr19	4800346	4843360	PLIN4
chr19	5843424	5892814	
chr19	6055439	6093630	
chr19	7447585	7494263	MAP2K7
chr19	8549191	8597085	CD320
chr19	9893946	9906881	ZNF561
chr19	10609398	10625040	RAVER1
chr19	10821894	10924477	DNM2
chr19	10973604	11012392	
chr19	11195872	11213766	DNM2
chr19	11230955	11263367	SMARCA4
chr19	11353793	11380147	DOCK6
chr19	12892449	12906321	IER2
chr19	13112500	13216575	
chr19	13255009	13322263	IER2
chr19	13945967	13956449	
chr19	13956585	13967073	NANOS3
chr19	13991072	14030133	CACNA1A
chr19	14088643	14135034	C19orf57
chr19	14153611	14196841	
chr19	14444164	14497035	
chr19	14615622	14634731	
chr19	15429981	15445397	
chr19	15485544	15491673	OR1I1
chr19	15939797	15949157	
chr19	16175036	16239139	
chr19	17189056	17268464	
chr19	17331090	17366461	
chr19	17422229	17468806	
chr19	17875523	17914613	PDE4C

chr19	18388904	18394729	
chr19	18428289	18435391	PDE4C
chr19	18611824	18621473	
chr19	18942170	18961749	
chr19	19475156	19601302	
chr19	33513199	33593993	CEBPA
chr19	33723635	33815979	SLC7A10
chr19	33855368	33904395	PEPD, KCTD15, CHST8, SLC7A10, LRP3
chr19	35485150	35505427	
chr19	35599346	35623141	FXYD3, LSR
chr19	35755412	35761152	TMEM147
chr19	36182954	36231499	TMEM147
chr19	38404755	38496194	C19orf33
chr19	38752966	38759461	CATSPERG
chr19	39125397	39230893	CATSPERG
chr19	39282919	39338169	LGALS4
chr19	39616222	39661839	
chr19	39887706	39902964	
chr19	40419549	40457265	
chr19	40898619	40941282	
chr19	41105830	41131955	
chr19	41219923	41227824	PRX
chr19	41295809	41338520	CYP2B7P
chr19	41664915	41683352	RAB4B
chr19	41914345	41942398	
chr19	42210448	42221456	CEACAM6
chr19	42233945	42247383	CEACAM6
chr19	42366254	42445578	
chr19	42676789	42723237	CEACAM8
chr19	42754754	42760715	LIPE
chr19	42781416	42790645	
chr19	44171684	44217864	
chr19	44267547	44289647	KCNN4
chr19	45183522	45207578	CEACAM16
chr19	45220249	45288652	BLOC1S3
chr19	45347676	45389453	CKM

chr19	45593294	45677813	NKPD1
chr19	45922666	45989212	FOSB
chr19	46008524	46032748	
chr19	46262286	46278443	FOSB
chr19	46365538	46386839	
chr19	47103387	47124861	
chr19	47216789	47247041	
chr19	47260797	47307254	
chr19	47578444	47636755	C5AR2
chr19	47728441	47736909	
chr19	47983534	48027142	
chr19	48101934	48171365	EHD2, MEIS3, PLA2G4C, ZNF541, SLC8A2, C5AR2
chr19	48262056	48284829	
chr19	48811905	48837687	PLA2G4C
chr19	48983477	49006295	LMTK3
chr19	49043282	49072203	SULT2B1, LMTK3
chr19	49114785	49123342	TMEM143
chr19	49178577	49202140	ZNF114, SEC1P
chr19	49374781	49379649	SYNGR4
chr19	49463258	49480601	
chr19	49627767	49675265	
chr19	50046431	50075143	FCGRT
chr19	50136758	50146335	
chr19	50668992	50738128	NR1H2
chr19	51320574	51347864	KLK3
chr19	53425844	53450400	ERVV-2
chr19	54345892	54393382	CACNG6
chr19	54690066	54696355	KIR3DX1
chr19	54969550	54977800	
chr19	54979246	54985496	
chr19	55577080	55602899	TMEM150B
chr19	55756765	55767392	
chr19	55848994	55853783	
chr19	55894375	55899811	
chr19	56168399	56214725	PTPRH
chr19	56592157	56669915	

chr19	59065229	59071596	
chr2	1587869	1662339	
chr2	8613832	8688760	
chr2	8705570	8785328	
chr2	8816205	8834116	ID2
chr2	9349698	9425572	
chr2	9878806	9972083	ITGB1BP1, YWHAQ, CPSF3
chr2	10146514	10201123	KLF11
chr2	10259805	10311039	
chr2	10357524	10446140	CYS1
chr2	10465173	10642070	CYS1
chr2	11487649	11547488	
chr2	11882894	11903569	ROCK2
chr2	14762896	14776676	
chr2	20291248	20442689	TTC32
chr2	20615422	20650844	SDC1
chr2	20767397	20842076	HS1BP3, GDF7
chr2	25554944	25566165	
chr2	26214174	26299626	
chr2	26672847	26745981	DRC1
chr2	27924636	27989801	GCKR, CAD, MPV17, EIF2B4, FNDC4, SUPT7L
chr2	28542414	28677214	
chr2	28806993	28914993	
chr2	28970251	28978251	
chr2	36582240	36651757	
chr2	39346751	39352498	
chr2	42273903	42369775	
chr2	43035897	43038998	
chr2	43132650	43314387	HAAO
chr2	43327804	43504749	
chr2	46503123	46543729	ATP6V1E2
chr2	47072749	47110258	
chr2	47173078	47336580	
chr2	47535049	47614017	TTC7A
chr2	54750916	54832239	
chr2	60693031	60784118	

chr2	62514847	62536640	
chr2	64828384	64877868	CEP68
chr2	65080428	65095143	
chr2	65604444	65664937	
chr2	68477086	68480722	
chr2	69971637	70035271	
chr2	70159060	70239677	
chr2	70295063	70336961	ASPRV1
chr2	70367342	70371248	
chr2	74197943	74265345	MOB1A
chr2	74666483	74670292	TTC31
chr2	75060443	75103512	
chr2	85049037	85116562	RETSAT
chr2	85146148	85223840	CAPG
chr2	85531154	85555765	ELMOD3
chr2	85623710	85699394	ATOH8
chr2	88421901	88459969	FABP1
chr2	96797386	96830711	
chr2	97189470	97223131	
chr2	97424315	97439514	CNNM4
chr2	97526110	97598121	FAM178B
chr2	98265837	98290977	
chr2	101429505	101442449	RPL31
chr2	101850423	101884755	
chr2	103234963	103285465	SLC9A2
chr2	105986237	106029596	
chr2	106042446	106085752	
chr2	109573243	109609883	EDAR
chr2	110417091	110474383	
chr2	110855017	110874614	
chr2	111874466	111893676	
chr2	113914222	114010081	IL36RN

chr2	114026412	114049132	IL36RN
chr2	114632201	114660496	
chr2	120160007	120199997	SCTR
chr2	120961029	121004791	RALB
chr2	121279843	121391313	TMEM185B
chr2	121942134	122061482	
chr2	127807485	127893131	PROC, ERCC3, BIN1, GYPC
chr2	128144372	128181433	
chr2	128377586	128436836	MAP3K2
chr2	129018217	129083764	UGGT1
chr2	134876211	134947511	
chr2	151328605	151344209	
chr2	157176182	157207548	
chr2	159979586	160017653	
chr2	161061150	161085083	PLA2R1
chr2	171567847	171574960	GAD1
chr2	173087737	173119964	
chr2	173193391	173226870	
chr2	173291291	173332405	
chr2	174824174	174832589	
chr2	178103901	178138996	
chr2	190364993	190424764	
chr2	191744139	191748184	
chr2	200294447	200349163	SATB2
chr2	201726459	201732633	
chr2	201979988	202023465	

chr2	206948329	206952121	
chr2	208027703	208032208	CPO
chr2	208392709	208405950	
chr2	208600224	208735780	PLEKHM3
chr2	218762724	218876498	RUFY4
chr2	219148537	219162345	
chr2	219258673	219268353	CYP27A1
chr2	219720208	219774230	TTLL4, STK36
chr2	219860927	219868129	SLC23A3
chr2	219921304	219926326	ANKZF1
chr2	219972239	219993603	
chr2	220036739	220049138	
chr2	220111229	220119671	WNT10A
chr2	220290802	220360906	ANKZF1
chr2	223288466	223305254	
chr2	227655649	227666540	
chr2	231523335	231586149	
chr2	231728493	231807493	CAB39, C2orf72
chr2	232226292	232290809	C2orf72
chr2	232461975	232581372	
chr2	233718083	233766855	ATG16L1
chr2	233842332	233883448	NEU2
chr2	233904834	233990319	
chr2	234239821	234294333	
chr2	234307968	234395713	UGT1A10
chr2	235859293	235962661	SH3BP4

chr2	236401625	236503968	
chr2	236567075	236633872	
chr2	238380339	238457019	
chr2	238568988	238653737	
chr2	239005365	239084053	SCLY
chr2	239169490	239219160	
chr2	239310380	239348264	
chr2	240123018	240271815	
chr2	241503593	241519545	C2orf54 (MAB21L4)
chr2	241540948	241546332	CAPN10, C2orf54 (MAB21L4)
chr2	241810618	241873778	CAPN10, C2orf54 (MAB21L4)
chr2	241895868	241951777	PASK
chr2	242242531	242260148	
chr2	242280263	242316360	
chr2	242429052	242445571	FARP2
chr2	242704267	242763347	GAL3ST2
chr2	242784751	242844988	ING5
chr20	1305575	1319030	
chr20	1783026	1879655	TGM3
chr20	3775883	3789909	
chr20	5621987	5640483	CRLS1, TRMT6, GPCPD1, C20orf196 (SHLD1)
chr20	5684386	5770892	CRLS1, GPCPD1, C20orf196 (SHLD1)
chr20	10633038	10656729	SLX4IP
chr20	16501069	16561521	SNRPB2
chr20	17546421	17553286	BFSP1
chr20	17583250	17665867	
chr20	17787417	17877043	ZNF133
chr20	17902067	17921342	
chr20	17986049	18040038	
chr20	19715270	19741176	

chr20	19840549	19975049	NAA20
chr20	22548732	22567099	
chr20	23083553	23147505	NXT1
chr20	23336843	23343803	
chr20	24997114	25072191	ABHD12, ACSS1, APMAP
chr20	25175129	25241923	
chr20	25257781	25341866	ZNF337
chr20	30147263	30200833	ID1
chr20	30248842	30311886	FOXS1
chr20	30789745	30845916	MYLK2, CCM2L, PLAGL2, C20orf112 (NOL4L)
chr20	31033456	31129997	POFUT1
chr20	32357743	32387396	RALY
chr20	32401625	32459298	NECAB3
chr20	33100110	33150719	MYH7B, MAP1LC3A
chr20	33290652	33301074	
chr20	33466933	33548685	
chr20	33839610	33917414	TRPC4AP, NFS1, GDF5, MMP24, GGT7, CPNE1
chr20	34651878	34737744	CPNE1, NFS1
chr20	35064315	35122508	
chr20	35887970	36040998	NNAT, MANBAL
chr20	36737140	36802296	TTI1, TGM2, CTNNBL1, KIAA1755
chr20	39744328	39802691	
chr20	39945365	39973640	PLCG1
chr20	42978793	43001116	GDAP1L1
chr20	43015616	43047283	
chr20	43060069	43094649	GDAP1L1
chr20	43964313	43977507	PABPC1L, SNX21
chr20	44034062	44065730	WFDC2
chr20	45932127	45991840	
chr20	46355492	46411358	
chr20	47200410	47315383	STAU1
chr20	47332704	47422242	CSE1L
chr20	47528689	47566298	
chr20	48290359	48335090	CEBPB
chr20	48380228	48462350	CEBPB
chr20	48524357	48561400	CEBPB

chr20	48720013	48789586	
chr20	48803102	49101366	CEBPB
chr20	49344337	49364919	MOCS3
chr20	50348503	50389782	SALL4
chr20	52194348	52241570	
chr20	52255892	52296764	
chr20	52352178	52423273	
chr20	52474079	52566458	
chr20	55950022	56056724	RAE1
chr20	56104107	56204341	PCK1
chr20	56229753	56295325	BMP7, RAE1, PMEPA1
chr20	56554953	56599579	
chr20	57463250	57471189	NELFCD
chr20	57571301	57591696	NELFCD, SLMO2
chr20	60625295	60645708	MTG2, PSMA7, ADRM1, LSM14B, CABLES2, SS18L1
chr20	60709691	60731948	PSMA7
chr20	60783185	60829682	SS18L1
chr20	60866815	60895837	LSM14B
chr20	60912703	60956778	MTG2, SS18L1, MRGBP, PSMA7, TAF4, ADRM1, LSM14B, CABLES2
chr20	60979569	60991115	
chr20	61270055	61300727	TCFL5
chr20	61424298	61430672	DIDO1
chr20	61505801	61562562	NKAIN4
chr20	61583036	61629300	OGFR, DIDO1, GID8, SLC17A9, BIRC7, ARFGAP1, YTHDF1
chr20	62150329	62155053	
chr20	62164679	62175099	
chr20	62312233	62336348	PRPF6, C20orf195 (FNDC11), HELZ2, SAMD10, YTHDF1, ABHD16B
chr20	62357798	62383498	SAMD10
chr20	62493840	62498077	
chr20	62578464	62591413	
chr20	62837634	62876976	TPD52L2, DNAJC5, SLC2A4RG, SAMD10, ABHD16B, ZGPAT, UCKL1, PCMTD2, LIME1
chr21	18865047	18900691	
chr21	34774817	34815129	
chr21	36236111	36283282	

chr21	37485227	37581944	
chr21	38737141	38747131	TTC3
chr21	40138244	40204595	PSMG1
chr21	40278501	40306294	ETS2
chr21	40320708	40407095	ETS2
chr21	41004658	41060316	
chr21	42525684	42552403	
chr21	42866102	42955540	TMPRSS2
chr21	43012823	43106321	C2CD2
chr21	43119523	43150298	RIPK4
chr21	43171945	43206984	UMODL1
chr21	43429153	43433521	
chr21	43718828	43759283	C2CD2
chr21	43870340	43914220	
chr21	43932564	43959840	UMODL1
chr21	44139132	44182145	PDE9A
chr21	44375437	44401511	U2AF1
chr21	44554821	44616839	PKNOX1, RRP1B
chr21	44694400	44783664	RRP1B
chr21	44858977	44873702	AGPAT3
chr21	45116091	45203714	CSTB
chr21	45279081	45324680	PFKL, TRAPPC10
chr21	45397210	45445209	TRAPPC10
chr21	45556368	45643372	RRP1
chr21	45658980	45670752	PFKL, CSTB
chr21	45712521	45727180	TRAPPC10
chr21	46255495	46302436	
chr21	46711365	46810661	PCBP3, POFUT2
chr21	46917222	46976311	SLC19A1
chr22	18243372	18270193	
chr22	18312985	18485260	PEX26
chr22	18538170	18575590	
chr22	19156076	19173402	HIRA
chr22	19829475	19890253	C22orf29 (RTL10)
chr22	19940999	20001192	ZDHHC8
chr22	20160883	20234381	ZDHHC8

chr22	20856835	20944344	
chr22	21289918	21337350	AIFM3
chr22	22290909	22316911	SDF2L1
chr22	23524799	23631169	
chr22	23860500	23880998	SLC2A11
chr22	24536186	24571357	SUSD2
chr22	24815198	24842330	SNRPD3
chr22	24938039	24952572	GUCD1
chr22	25338068	25482898	GUCD1
chr22	25505870	25565876	KIAA1671
chr22	25759644	25802946	KIAA1671
chr22	28026276	28083459	TTC28
chr22	29177960	29239632	XBP1
chr22	29399573	29445886	ZNRF3
chr22	30631895	30659464	SLC35E4
chr22	30673897	30714644	SEC14L3
chr22	31440389	31501305	
chr22	31603912	31650966	
chr22	31666929	31694556	
chr22	31735466	31744151	
chr22	32008931	32042435	
chr22	35695169	35726874	
chr22	35822544	35885660	MB
chr22	36695421	36870195	
chr22	37406071	37423323	
chr22	37558206	37596906	TST
chr22	37662994	37830188	CSF2RB
chr22	37894340	37901230	CARD10
chr22	37955509	37961165	GGA1
chr22	38031888	38040007	
chr22	38122338	38150255	SH3BP1
chr22	38170192	38215249	CARD10
chr22	38493842	38517899	
chr22	38539997	38666282	
chr22	38678745	38715980	JOSD1
chr22	39085277	39114315	JOSD1

chr22	39128208	39162004	
chr22	39316861	39364502	APOBEC3B
chr22	39629563	39706904	PDGFB
chr22	39824725	39871784	CBX7, MGAT3, PDGFB, SYNGR1, TAB1
chr22	40380994	40420706	
chr22	41397098	41419171	MKL1
chr22	41791846	41815145	ACO2
chr22	41839294	41844713	TEF
chr22	42217956	42273543	ACO2, TEF
chr22	42299203	42340539	
chr22	42667765	42797051	
chr22	43038895	43046055	PACSIN2
chr22	43264661	43439013	
chr22	43513737	43526402	SCUBE1
chr22	46262795	46352015	GTSE1
chr22	46406761	46411260	PPARA
chr22	46445694	46452947	
chr22	46458772	46478191	
chr22	46478760	46487142	CELSR1
chr22	46497766	46507175	CELSR1
chr22	46542554	46569987	TTC38, PKDREJ, GRAMD4
chr22	46748627	46787408	CELSR1
chr22	46824524	46862283	
chr22	46937738	47092435	CERK
chr22	47118572	47226600	
chr22	50209411	50261230	
chr22	50319146	50364826	
chr22	50421427	50473904	PLXNB2
chr22	50617874	50646284	
chr22	50711151	50756460	
chr22	50878422	50923490	
chr22	50962309	50971267	
chr3	4753453	4819425	BHLHE40
chr3	5017868	5069286	
chr3	9436892	9444823	
chr3	12432252	12523307	

chr3	12985029	13067745	TMEM40, WNT7A
chr3	13512116	13536382	WNT7A
chr3	14298104	14378584	TMEM43
chr3	14399898	14514536	TMEM43
chr3	32440189	32475162	CMTM7
chr3	38025920	38036323	ACAA1
chr3	38126526	38196711	
chr3	39166795	39197717	WDR48
chr3	41240003	41247161	
chr3	42052123	42077635	
chr3	42159038	42232502	VIPR1
chr3	42514128	42587318	VIPR1, HIGD1A
chr3	42841612	42871959	VIPR1, HIGD1A
chr3	45097761	45183848	
chr3	45624504	45689506	
chr3	46424499	46496770	
chr3	46730797	46756211	
chr3	46930314	47040581	
chr3	47436522	47486519	
chr3	48463128	48478174	NCKIPSD
chr3	48505764	48520060	PRKAR2A
chr3	48654866	48675762	IP6K2
chr3	48731379	48785020	SLC25A20
chr3	49054656	49060694	PRKAR2A
chr3	49934784	49945759	
chr3	50160297	50217836	
chr3	50247646	50312226	HYAL3
chr3	50334300	50341598	HYAL1
chr3	50351804	50372211	
chr3	50390536	50495807	RBM5
chr3	50623949	50676454	
chr3	51421594	51438331	
chr3	52057782	52125318	
chr3	52318123	52326957	
chr3	53166834	53230973	
chr3	53256227	53310221	

chr3	53854783	53881180	
chr3	57928945	58057559	
chr3	58612512	58692124	FAM3D
chr3	66490553	66558796	
chr3	71074080	71128384	
chr3	71164955	71204485	
chr3	71612437	71634637	
chr3	101553239	101597714	
chr3	107241446	107245110	
chr3	119811908	119817478	COX17
chr3	124670251	124722800	
chr3	126175121	126216648	UROC1
chr3	126679289	126725730	PLXNA1
chr3	126734654	126772223	
chr3	127449108	127543524	
chr3	128722169	128787733	
chr3	129266205	129352965	EFCAB12
chr3	134018149	134102139	
chr3	135905201	135917190	
chr3	141045619	141097069	RNF7
chr3	141112097	141175373	SPSB4
chr3	142314195	142344869	
chr3	149057776	149120135	TM4SF4
chr3	150124943	150129391	
chr3	150441886	150482605	
chr3	151976190	151993419	
chr3	152876999	152881412	
chr3	156787741	156808707	

chr3	156823048	156855928	
chr3	169375755	169387326	
chr3	169755228	169768436	
chr3	176845712	176942803	
chr3	176997149	177021902	
chr3	177053032	177080948	
chr3	182963086	183004865	
chr3	184062273	184091023	
chr3	184264246	184322960	EPHB3
chr3	185244883	185279994	
chr3	187455191	187469615	MASP1
chr3	193530541	193629340	
chr3	193746969	193879826	GP5
chr3	195500894	195660661	MUC20
chr3	195886533	195955303	SLC51A
chr3	196712324	196763744	WDR53
chr3	197181501	197287034	
chr4	673912	733742	ZNF141
chr4	759294	809478	ZNF141
chr4	941817	968153	DGKQ
chr4	984645	1053440	FGFRL1
chr4	1160762	1216551	FGFRL1
chr4	1234500	1244656	FGFRL1
chr4	1323168	1345842	
chr4	1492816	1584306	
chr4	1716992	1733011	
chr4	1748180	1818534	HAUS3
chr4	1839929	1910752	LETM1
chr4	2387092	2464880	ZFYVE28
chr4	2742329	2770858	

chr4	2787960	2876249	
chr4	2924111	2945713	
chr4	3287169	3342858	HTT
chr4	6678674	6698695	S100P
chr4	6722091	6782024	TADA2B
chr4	6887698	6972889	TADA2B
chr4	7957954	7988605	ABLIM2
chr4	8077812	8132727	ABLIM2
chr4	8174090	8208427	
chr4	10076448	10126184	ZNF518B
chr4	15002818	15007221	
chr4	25836357	25867877	SEL1L3
chr4	37431895	37492229	
chr4	38063934	38084821	PTTG2
chr4	38129750	38165338	
chr4	38663021	38693644	
chr4	40439724	40589443	
chr4	56259521	56265572	
chr4	74960723	74983685	PPBP
chr4	77484965	77548505	
chr4	77584194	77637557	
chr4	90195986	90229685	
chr4	103740245	103751132	
chr4	111114430	111120414	
chr4	111531763	111555575	
chr4	124315353	124341272	
chr4	140096935	140110107	
chr4	141056739	141080210	
chr4	141157126	141174915	
chr4	146855605	146860416	
chr4	149348610	149367220	
chr4	152233610	152283822	

chr4	153856253	153880544	ARFIP1
chr4	154124664	154197286	
chr4	159692385	159759774	
chr4	160023546	160026111	
chr4	185202302	185398387	
chr4	187644099	187688085	
chr5	474795	609377	ZDHHC11
chr5	756303	785279	
chr5	979520	1031369	CEP72, ZDHHC11, SLC12A7, SLC6A3, LPCAT1
chr5	1073524	1188157	CLPTM1L, ZDHHC11, TRIP13, CEP72, LPCAT1, SLC6A18
chr5	1470271	1520245	LPCAT1
chr5	6701302	6737323	NSUN2, SRD5A1, MED10
chr5	10284082	10310886	CMBL
chr5	10700031	10762126	
chr5	14142643	14227377	
chr5	16483144	16554639	
chr5	16845602	16933989	
chr5	32298136	32333194	
chr5	32433949	32447867	
chr5	40676729	40700663	
chr5	42982801	43011206	
chr5	43034226	43043691	
chr5	43063838	43068028	
chr5	56110126	56113613	
chr5	60614482	60640020	ZSWIM6
chr5	67510489	67581694	
chr5	68787670	68827176	MARVELD2
chr5	72409581	72432342	TMEM171
chr5	73927127	73940199	ENC1
chr5	75752852	75789307	F2RL1
chr5	79471332	79566020	SERINC5
chr5	90675237	90680688	
chr5	95143290	95179284	

chr5	95983382	96008401	ERAP1
chr5	98108333	98130880	
chr5	115854281	115911095	SEMA6A
chr5	126333946	126378182	MARCHF3
chr5	127414078	127426618	
chr5	130580118	130649740	
chr5	131581537	131616291	RAD50
chr5	131689653	131724473	SLC22A5, SOWAHA
chr5	131749771	131841995	SOWAHA
chr5	132142089	132177705	SHROOM1
chr5	133328151	133407727	TCF7, VDAC1
chr5	133768336	133803024	C5orf24
chr5	133837574	133866308	C5orf24
chr5	134359884	134384113	PITX1
chr5	134659432	134733027	
chr5	139013411	139091824	PSD2
chr5	139104655	139149002	PSD2
chr5	139525353	139574291	CYSTM1
chr5	139595437	139650352	WDR55
chr5	141211083	141263248	KIAA0141
chr5	142160477	142260069	
chr5	148818221	148868910	SLC26A2
chr5	148982530	149060869	ARHGEF37
chr5	149106862	149267616	PDE6A, SLC26A2
chr5	149539500	149568866	TIGD6
chr5	149586118	149597726	HMGXB3
chr5	149826297	149924557	TIGD6

chr5	149941195	150017647	GPX3
chr5	150431504	150530637	SLC36A1
chr5	154133189	154173660	
chr5	159670932	159735006	CCNJL
chr5	167696280	167757224	
chr5	168078410	168111185	PANK3
chr5	170171162	170201193	GABRP
chr5	171530041	171615934	
chr5	172195280	172206972	NEURL1B
chr5	172327125	172346151	ERGIC1
chr5	173191034	173230786	BOD1
chr5	175819506	175855866	KIAA1191
chr5	175956664	176001108	KIAA1191
chr5	176722030	176736996	
chr5	177621106	177679790	PHYKPL
chr5	179229305	179235969	HNRNPH1
chr5	179243293	179248952	
chr5	180212330	180259493	
chr6	1310123	1336593	FOXQ1
chr6	1588582	1644562	
chr6	2210828	2260848	
chr6	2783063	2870724	
chr6	2980675	2992105	
chr6	3052909	3070261	
chr6	3261068	3451653	
chr6	6677371	6760218	
chr6	6789844	6827596	
chr6	7042214	7062450	
chr6	7078402	7279359	

chr6	7531455	7563037	
chr6	10403652	10427002	MAK
chr6	11187519	11237258	
chr6	11253508	11298471	
chr6	11750299	11815661	
chr6	12007326	12027235	
chr6	18260830	18278067	
chr6	20401995	20428889	
chr6	21587001	21598331	SOX4, CDKAL1
chr6	24718942	24722740	
chr6	30058788	30124973	ZNRD1
chr6	30691362	30762214	POU5F1
chr6	30843457	30855039	IER3
chr6	31141202	31190866	HLA-B
chr6	31701197	31709066	GPANK1
chr6	31785704	31800301	ATP6V1G2-DDX39B
chr6	31833534	31856917	GPANK1
chr6	32933910	32951015	
chr6	33544245	33549210	
chr6	33586854	33594477	
chr6	33660014	33749771	
chr6	34482430	34578000	SPDEF
chr6	35438934	35474161	
chr6	35691386	35701797	
chr6	36079393	36101298	C6orf222 (BNIP5)
chr6	36277080	36337407	
chr6	36564821	36652372	C6orf222 (BNIP5)
chr6	36683212	36774267	CDKN1A
chr6	36965965	37029694	PPIL1
chr6	39134334	39219221	KCNK5
chr6	41392111	41438969	
chr6	41455576	41571011	FOXP4
chr6	41650271	41737635	FOXP4
chr6	42056271	42115051	TFEB
chr6	42729722	42758905	
chr6	43214249	43259508	CRIP3

chr6	43635117	43712583	CAPN11
chr6	43726225	43824146	VEGFA
chr6	44002034	44103449	HSP90AB1, C6orf223, VEGFA, SLC35B2
chr6	44184479	44206134	CDC5L
chr6	45914268	45984446	
chr6	52167993	52229585	
chr6	64279147	64286590	
chr6	74223561	74234363	
chr6	89788647	89793074	
chr6	106950828	106973629	
chr6	107137094	107200629	
chr6	108877707	108887837	SESN1
chr6	109611453	109673719	
chr6	109688096	109706678	
chr6	109764627	109788761	FIG4
chr6	111195070	111205022	
chr6	111910301	111954238	TRAF3IP2
chr6	114175529	114194882	
chr6	128799398	128837581	
chr6	135534680	135594210	
chr6	137278355	137291267	
chr6	137344690	137366749	IL20RA
chr6	137536873	137578524	SLC35D3
chr6	138426044	138430485	
chr6	143264915	143269254	ADAT2
chr6	143856647	143876546	
chr6	150489302	150526550	PPP1R14C
chr6	151701476	151714265	RMND1
chr6	157097603	157103712	

chr6	158350244	158390905	ZDHHC14
chr6	158405444	158493053	
chr6	158980595	158996671	OSTCP1
chr6	159049669	159089680	
chr6	159159682	159241230	
chr6	159255811	159292507	
chr6	160100618	160123604	
chr6	167363134	167384572	
chr6	168062964	168162316	TTLL2, KIF25
chr6	168413323	168503148	KIF25
chr6	170574513	170606836	FAM120B
chr7	115873	189300	FAM20C
chr7	298552	336254	PDGFA
chr7	357264	431053	FAM20C, PDGFA, PRKAR1B
chr7	449093	615004	FAM20C, PDGFA
chr7	640661	681000	GPR146, PDGFA
chr7	833693	876996	
chr7	948975	956672	ADAP1
chr7	959479	969152	
chr7	983984	990414	SUN1, GET4
chr7	991118	994980	
chr7	1013987	1020134	CYP2W1, GPR146, GPER1, INTS1, PRKAR1B, PDGFA, GET4
chr7	1065425	1124042	SUN1
chr7	1184061	1233794	GPR146
chr7	1486311	1514796	TMEM184A
chr7	1568954	1574711	
chr7	1605706	1611646	
chr7	2477451	2577892	LFNG
chr7	2645525	2776475	BRAT1, TTYH3
chr7	4721270	4794293	
chr7	5391430	5495983	ZNF815P
chr7	5565783	5654698	EIF2AK1

chr7	6401818	6443481	
chr7	20368046	20394977	
chr7	22369279	22401681	
chr7	25891379	25906154	
chr7	25979727	26008574	
chr7	26191251	26209207	NFE2L3, CBX3
chr7	26222470	26245710	
chr7	27140197	27149295	HOXA1
chr7	27180124	27188836	
chr7	27193128	27210499	
chr7	27210692	27226589	HOXA2
chr7	27226673	27235671	HOXA5
chr7	27236867	27251265	HOXA5
chr7	27773847	27784785	HIBADH
chr7	29229287	29248948	CHN2
chr7	29601890	29607677	
chr7	30322996	30327105	
chr7	30932614	30978578	
chr7	36297675	36332564	
chr7	44111154	44152646	
chr7	44650171	44685168	
chr7	47389674	47426372	
chr7	47468138	47651644	PKD1L1
chr7	48124266	48137839	
chr7	51290755	51396780	
chr7	55089642	55164643	
chr7	55572151	55641166	
chr7	66100110	66162918	KCTD7
chr7	70158103	70166365	
chr7	73113983	73134548	STX1A
chr7	73148980	73158989	MLXIPL
chr7	73180110	73189479	CLDN4
chr7	73235441	73248623	CLDN4
chr7	73385253	73483905	BAZ1B
chr7	73673176	73747387	CLIP2
chr7	73865224	73961408	GTF2IRD1

chr7	74018395	74075950	GTF2IRD1
chr7	75550140	75622127	
chr7	75864295	75949165	POR
chr7	75993211	76034140	UPK3B
chr7	77281637	77331514	
chr7	87835205	87863163	RUNDC3B
chr7	92461754	92466516	PEX1
chr7	97905866	97925528	
chr7	97972452	98050516	
chr7	98805998	98828283	TMEM130
chr7	98969610	98991863	
chr7	99592062	99634221	FAM200A
chr7	99750229	99756770	
chr7	100030516	100053867	ZKSCAN1
chr7	100067090	100091823	ZKSCAN1
chr7	100270632	100292722	SLC12A9
chr7	100389991	100435450	TRIP6, ZAN, VGF
chr7	100482826	100510426	CLDN15
chr7	100607523	100631215	AGFG2
chr7	100720585	100766288	TRIP6, NAT16, VGF
chr7	100873719	100896396	CLDN15
chr7	101176441	101288724	VGF, NAT16
chr7	101331053	101391399	MYL10
chr7	101544929	101606786	
chr7	102786993	102794793	
chr7	104581371	104587535	
chr7	104645946	104657826	
chr7	113722871	113728455	
chr7	116411019	116453647	
chr7	117294035	117326750	CTTNBP2

chr7	129588117	129614129	
chr7	129780377	129795177	TMEM209
chr7	130567798	130646840	
chr7	130667636	130757779	
chr7	138780203	138811181	
chr7	139746413	139763577	
chr7	139900225	139971468	
chr7	140174574	140227308	TBXAS1
chr7	142102038	142134538	
chr7	143064821	143092975	
chr7	150064720	150073325	REPIN1
chr7	150099712	150106164	
chr7	150477778	150505955	TMEM176B
chr7	150753888	150760769	AGAP3
chr7	150776486	150781126	
chr7	151493628	151575818	CRYGN, WDR86, GALNT11
chr7	154994800	155029752	
chr7	155076689	155092799	INSIG1
chr7	155579781	155659314	SHH
chr7	155743542	155757869	CNPY1, SHH
chr7	156393561	156419142	
chr7	156791706	156833174	UBE3C
chr7	157065075	157111568	
chr7	158642204	158662120	
chr8	1913890	1926709	
chr8	8263120	8326038	
chr8	8702030	8757987	

chr8	8910478	8963645	
chr8	11274378	11332746	
chr8	11652280	11681536	FAM167A
chr8	11701001	11764237	FAM167A
chr8	17636013	17664074	
chr8	22025073	22086488	SORBS3
chr8	22209326	22250863	PDLIM2
chr8	22405454	22456585	SLC39A14
chr8	22596537	22639491	SORBS3
chr8	22921421	22964018	
chr8	23380882	23428928	
chr8	27189458	27266603	
chr8	28196552	28270528	
chr8	29110995	29154030	
chr8	29197510	29210928	DUSP4
chr8	30356631	30426729	RBPMS
chr8	37400662	37425796	ZNF703
chr8	37446378	37513231	
chr8	37532976	37561473	ERLIN2
chr8	37639460	37773872	
chr8	38234927	38241308	
chr8	38613511	38663892	
chr8	38757313	38816236	
chr8	39897244	39920242	
chr8	42744847	42772855	
chr8	48262147	48294509	CEBPD
chr8	48421653	48455025	SPIDR
chr8	48646118	48651966	
chr8	61811816	61840052	CLVS1
chr8	76314903	76322046	
chr8	81397760	81421513	
chr8	95625352	95666522	
chr8	96077211	96128322	INTS8
chr8	101312661	101349943	ANKRD46
chr8	101955916	101966785	PABPC1

chr8	101997511	102041060	ANKRD46
chr8	102089173	102179732	PABPC1
chr8	102503622	102533609	ZNF706
chr8	103628766	103675494	
chr8	103740248	103825586	
chr8	110591230	110620476	SYBU
chr8	119086620	119139341	
chr8	121820726	121825333	MTBP
chr8	126415742	126448632	SQLE, NSMCE2
chr8	126649390	126677556	NSMCE2
chr8	127566250	127571455	MYC
chr8	128573356	128616312	MYC
chr8	128746466	128755847	POU5F1B
chr8	134256633	134315934	NDRG1
chr8	134335470	134391007	
chr8	135843061	135845900	
chr8	141516428	141523242	
chr8	141552714	141642515	CHRAC1
chr8	142082599	142174944	
chr8	142203985	142256046	SLC45A4
chr8	142273921	142299957	SLC45A4, PTP4A3, DENND3, PTK2
chr8	142315603	142357428	PTP4A3
chr8	142422272	142429622	PTP4A3
chr8	144089481	144109882	ZC3H3, RHPN1, LY6E, LY6D, ZFP41
chr8	144342533	144368943	ZC3H3, RHPN1, CYP11B2, TIGD5, MAPK15, MAFA, TOP1MT
chr8	144464166	144529325	ZC3H3, RHPN1, TIGD5, MAPK15, MAFA

chr8	144653444	144663891	RHPN1
chr8	144813419	144823974	ZC3H3, TOP1MT, ZFP41, SCRIB, GRINA, RHPN1
chr8	144886670	144908520	ZC3H3
chr8	144938952	144989216	ZC3H3
chr8	145005703	145029567	ZC3H3
chr8	145043674	145052590	ZC3H3
chr8	145536936	145562791	ARHGAP39
chr8	145577602	145584798	
chr8	145637532	145659835	ZNF34
chr8	145724875	145736551	
chr9	2014195	2023271	
chr9	14309511	14323573	
chr9	19229675	19233894	
chr9	27360698	27473689	
chr9	33128522	33168546	AQP3
chr9	33202488	33255307	SPINK4, AQP3
chr9	33287280	33298803	
chr9	33813398	33820021	
chr9	35904158	35925009	HRCT1
chr9	36120900	36166748	
chr9	36290824	36329566	HRCT1
chr9	37911456	38075786	
chr9	71324166	71364433	
chr9	73006949	73036884	KLF9
chr9	74373059	74384783	
chr9	74977205	74981427	
chr9	77686385	77727120	
chr9	80618409	80650066	
chr9	91968129	92116567	CKS2
chr9	92134062	92172043	
chr9	92263575	92302155	GADD45G
chr9	93909435	93975909	

chr9	95492230	95537743	NOL8
chr9	95929205	96033139	
chr9	96211941	96274344	
chr9	96312205	96383980	
chr9	97362246	97412003	
chr9	97765305	97771070	C9orf3 (AOPEP)
chr9	98248725	98275129	
chr9	99175902	99183433	CDC14B
chr9	100821574	100909505	
chr9	100925141	100958251	
chr9	100971057	101016512	
chr9	108009532	108095645	
chr9	110243788	110268173	KLF4
chr9	110348570	110402367	
chr9	112008471	112108750	EPB41L4B
chr9	112943440	112984316	
chr9	113014239	113022612	
chr9	114653204	114671387	PTGR1
chr9	114908851	114943306	
chr9	115067730	115101623	
chr9	116131964	116161408	
chr9	116265295	116396869	
chr9	117124745	117165720	
chr9	123618299	123698895	PHF19
chr9	124023380	124102025	GSN
chr9	124360204	124528771	
chr9	126081274	126144032	ZBTB26
chr9	127018737	127085026	

chr9	129092046	129142270	
chr9	130149295	130165256	SH2D3C, LRSAM1, ENG, SLC2A8, GARNL3, LRSAM1
chr9	130256294	130351541	
chr9	130587141	130647070	STXBP1
chr9	130664513	130684554	ST6GALNAC6
chr9	130704349	130755491	NAIF1
chr9	130864490	130880714	FAM102A
chr9	130905976	130914424	
chr9	131622527	131680147	
chr9	131811455	131850372	
chr9	131895420	131909835	PHYHD1
chr9	131924858	131947259	
chr9	132094411	132124868	PTGES
chr9	132143494	132269806	PTGES
chr9	132310662	132376115	PTGES
chr9	133223375	133329315	FNBP1
chr9	134103506	134158677	
chr9	134224427	134309073	
chr9	134496283	134555283	
chr9	134571356	134615946	
chr9	135924105	135940404	ADAMTSL2, GBGT1, GTF3C5, ADAMTS13, CELP, CEL
chr9	135966206	136027438	
chr9	136099223	136160808	
chr9	136689130	136859572	DBH
chr9	136914345	136934837	
chr9	137144521	137367806	

chr9	138857212	138909902	
chr9	138967415	139090962	CAMSAP1
chr9	139404968	139525089	
chr9	139578763	139591286	CCDC183
chr9	139683661	139691005	MAMDC4
chr9	139739767	139758297	CCDC183
chr9	139774959	139808829	TMEM210
chr9	139836298	139847672	C9orf172 (AJM1)
chr9	140087151	140096306	C9orf172 (AJM1)
chr9	140122472	140135480	CCDC183
chr9	140169673	140176591	NRARP
chr9	140186990	140203083	GRIN1
chr9	140203344	140212896	MAMDC4, C9orf173 (STPG3), LCN12
chr9	140500027	140510499	NRARP
chrX	12988547	13048990	
chrX	23811271	23841815	
chrX	39812121	39874657	
chrX	39941636	39969370	BCOR
chrX	40000296	40036731	BCOR
chrX	41189758	41195978	
chrX	68047881	68069392	
chrX	70835396	70848211	
chrX	118611219	118647263	
chrX	129193853	129255773	XPNPEP2
chrX	152950541	152975505	TMEM187
chrX	152988183	152993943	
chrX	153186855	153196221	ARHGAP4
chrX	153236006	153240516	LAGE3

chrX	153264816	153326419	SRPK3
chrX	153576924	153613104	MECP2

Supplementary Table 3: List of commercially available CRC cell lines commonly used in laboratory studies.

HCT15
HCT116
COLO320
CACO2
HT29
DLD1
LS180
RKO
SW480
SW620
COLO741
SW480
COLO205

Supplementary Table 4: Hg19 coordinates of super-enhancers identified in primary CRC and normal colon tissue that are over- or under-represented in 13 commercial CRC cell lines.

'chr'	'start'	'end'	log2 cell lines/[illegible]	P-value
chr2	219921304	219926326	-3.933334181	4.75E-06
chr19	1282741	1294598	-3.032709913	7.60E-05
chr16	49886677	49893398	-2.949821466	0.000519631
chr19	42210448	42221456	-2.898911644	0.004772279
chr11	312970	318010	-2.894756705	1.76E-05
chr8	142422272	142429622	-2.644337941	0.001276849
chr19	950367	958117	-2.495988143	0.007449326
chr5	149539500	149568866	-2.335044971	1.38E-06
chr7	27226673	27235671	-2.171925116	0.002112967
chr7	100607523	100631215	-2.158768857	7.53E-06
chr11	76773786	76787721	-2.116481306	9.32E-10
chr3	38025920	38036323	-2.084897042	1.80E-06
chr1	47644434	47659486	-2.078868512	1.74E-06
chr7	113722871	113728455	-2.054225966	0.003951842
chr16	31137030	31147852	-2.037706389	1.65E-08
chr19	1129240	1134649	-2.035016076	5.13E-09
chr17	3856496	3892930	-2.015277075	9.50E-07
chr11	76796563	76803020	-2.005698624	1.14E-06
chr10	85918778	85965078	-1.956797767	6.89E-07
chr16	67460809	67504574	-1.945311673	1.21E-07
chr9	130905976	130914424	-1.91203976	0.000189521
chr14	94848020	94862793	-1.890862615	0.009692258
chr20	22548732	22567099	-1.887185684	0.001244224
chrX	70835396	70848211	-1.868040926	0.000105237

chr17	74485442	74490614	-1.851351981	2.51E-05
chr7	70158103	70166365	-1.815465956	2.41E-05
chr7	73180110	73189479	-1.810669041	1.10E-05
chr2	14762896	14776676	-1.803198778	0.000146674
chr8	142315603	142357428	-1.803098563	0.00033056
chr1	205532653	205568500	-1.800685286	0.000129067
chr8	48646118	48651966	-1.760217332	6.43E-08
chr7	156393561	156419142	-1.759424939	0.000451767
chr7	150477778	150505955	-1.753099976	1.77E-05
chr16	85449100	85526770	-1.752547912	0.000214569
chr16	582919	587823	-1.75068509	5.44E-06
chr4	15002818	15007221	-1.704048064	8.80E-08
chr19	18611824	18621473	-1.694070408	8.11E-07
chr19	40419549	40457265	-1.692289026	0.000447315
chr9	140186990	140203083	-1.674837677	8.94E-05
chr1	33342282	33373754	-1.670767379	3.25E-06
chr4	149348610	149367220	-1.66504665	4.70E-07
chr17	73634586	73644264	-1.663655009	0.000271978
chr8	76314903	76322046	-1.663653639	0.000200429
chr4	160023546	160026111	-1.660029638	2.21E-06
chr19	50668992	50738128	-1.654229726	1.39E-05
chr20	60979569	60991115	-1.651679206	0.001274464
chr16	31487207	31500133	-1.646142188	2.01E-05
chr1	1056306	1073787	-1.625633377	4.37E-05
chr7	27140197	27149295	-1.590910353	0.002221693
chr15	68479899	68503417	-1.588524898	3.11E-08
chr22	31666929	31694556	-1.583014828	6.13E-07
chr19	33723635	33815979	-1.576082011	4.14E-06
chr16	53163214	53168804	-1.562522215	0.001147531
chr16	68384973	68432029	-1.562204677	1.68E-07
chr17	37779956	37790775	-1.552808833	3.36E-05
chr21	43718828	43759283	-1.549372024	0.000136642
chr22	38493842	38517899	-1.539972117	5.68E-07
chr20	17902067	17921342	-1.522326512	2.30E-06
chr16	30123335	30135353	-1.518808525	9.54E-06
chr2	241540948	241546332	-1.517004878	0.000736344
chr8	144938952	144989216	-1.503213086	6.17E-06
chr19	35599346	35623141	-1.500696097	0.000173606
chr22	50319146	50364826	-1.495054142	2.29E-06
chr9	92263575	92302155	-1.49031086	1.83E-06
chr18	28676993	28682939	-1.489553055	0.001023615
chr11	2930168	2936397	-1.486304394	1.34E-05
chr9	130864490	130880714	-1.485125129	8.31E-05
chr4	1748180	1818534	-1.482977267	0.000172748
chr1	16059949	16070046	-1.480374457	0.000183684

chr9	95929205	96033139	-1.480201283	3.42E-06
chr2	219148537	219162345	-1.476482263	0.001992198
chr19	41914345	41942398	-1.473144331	1.38E-07
chr18	19741865	19781074	-1.464659281	0.000491763
chr17	38327873	38351879	-1.464426274	6.49E-05
chr2	239169490	239219160	-1.463708595	1.68E-07
chr7	29601890	29607677	-1.452014835	0.001734837
chr17	81005827	81069196	-1.442303168	1.51E-05
chr2	242704267	242763347	-1.441783842	7.54E-06
chr13	27522316	27597035	-1.42852943	0.008113301
chr15	31616912	31698718	-1.42833867	2.07E-07
chr19	55756765	55767392	-1.42726789	0.006850812
chr10	134197375	134245029	-1.427197048	5.09E-09
chr1	61508480	61523688	-1.412822061	2.25E-05
chr9	140087151	140096306	-1.410234098	0.006737055
chr16	67423853	67447927	-1.407506127	1.04E-05
chr17	79763639	79788092	-1.388498587	8.97E-08
chr10	134257773	134301784	-1.380589018	7.23E-06
chr1	199990289	200023200	-1.374864468	0.002411763
chr3	53166834	53230973	-1.374491386	8.47E-08
chr8	142203985	142256046	-1.372888112	6.06E-05
chr3	71612437	71634637	-1.372196278	0.000179723
chr19	54979246	54985496	-1.363581356	0.000548464
chr14	38052216	38072548	-1.363489967	0.002279186
chr9	134496283	134555283	-1.35721224	0.005416658
chr22	37406071	37423323	-1.357177296	4.84E-05
chr7	30322996	30327105	-1.355003336	6.64E-06
chr12	6468473	6473930	-1.354950301	0.000145933
chr4	140096935	140110107	-1.354221017	1.10E-07
chr4	7957954	7988605	-1.340452856	1.33E-05
chr17	26120397	26151772	-1.335406068	0.000614658
chr11	1525034	1597870	-1.334335	6.89E-06
chr5	40676729	40700663	-1.333969521	0.005834136
chr7	27236867	27251265	-1.333213185	0.008969102
chr5	149586118	149597726	-1.327771064	0.000134751
chr1	155050463	155059415	-1.32584885	0.000636383
chr19	4034556	4150644	-1.318671308	4.72E-08
chr17	73568797	73577073	-1.314979465	3.52E-05
chr14	55031455	55035147	-1.314499318	0.00014642
chr9	117124745	117165720	-1.313797327	0.000198119
chr17	37827160	37837462	-1.311567369	0.000914851
chr7	991118	994980	-1.306250732	0.006732574
chr8	1913890	1926709	-1.295545411	0.000272981
chr12	121650947	121685811	-1.29244219	0.001279391
chr19	1247317	1256284	-1.290320659	7.94E-05

chr7	25979727	26008574	-1.288425537	0.005901451
chr16	11290981	11360138	-1.28593865	2.36E-06
chr8	145536936	145562791	-1.282480618	2.95E-05
chr5	1073524	1188157	-1.28018389	0.000552488
chr2	43035897	43038998	-1.279825977	7.20E-09
chr9	139578763	139591286	-1.278519633	5.51E-05
chr1	51761624	51802404	-1.276336427	7.99E-06
chr19	35485150	35505427	-1.273803969	2.21E-06
chr21	42866102	42955540	-1.271553665	0.003990368
chr9	140203344	140212896	-1.268492344	0.003718446
chr7	948975	956672	-1.267631416	0.000589779
chr7	100482826	100510426	-1.262130127	4.80E-06
chr11	64654508	64663543	-1.26061998	0.001093014
chr9	110243788	110268173	-1.251133259	0.000442417
chr1	226288057	226325426	-1.248226489	8.32E-07
chr12	32543708	32556907	-1.244848297	1.23E-06
chr18	60381757	60385355	-1.235852828	3.79E-05
chr20	57463250	57471189	-1.214365622	0.000611273
chr5	32433949	32447867	-1.213848352	0.000267944
chr19	923608	929931	-1.211890102	0.004619643
chr21	45658980	45670752	-1.200354979	0.001115286
chr17	76988200	76995813	-1.19781147	0.002996369
chr3	169375755	169387326	-1.197762721	0.00030491
chr15	40634403	40643032	-1.192001967	0.003206032
chr9	100821574	100909505	-1.19111333	7.61E-05
chr11	818110	822789	-1.190429173	1.76E-05
chr1	19965334	19984929	-1.189885971	0.00011409
chr3	127449108	127543524	-1.181684473	0.000712362
chr17	73519791	73524830	-1.179960513	4.78E-05
chr5	172195280	172206972	-1.177488882	6.76E-05
chr11	117944260	117963999	-1.175196218	0.001450218
chr20	62164679	62175099	-1.174885529	0.002258229
chr19	12892449	12906321	-1.172881308	1.57E-06
chr6	35438934	35474161	-1.172216596	4.52E-06
chr1	1092834	1107947	-1.171099652	0.002064843
chr17	18864569	18923164	-1.170180749	0.005223225
chr1	17828633	17961597	-1.162184065	0.000116939
chr1	32127999	32154793	-1.1590256	0.001938253
chr1	233743752	233761980	-1.158896275	0.001413337
chrX	68047881	68069392	-1.15695134	0.001230527
chr16	81299560	81312418	-1.156884193	3.83E-05
chr2	206948329	206952121	-1.152646404	2.07E-07
chr6	157097603	157103712	-1.152413919	0.000154177
chr1	1076920	1083409	-1.149284102	0.002793414
chr15	31725421	31791946	-1.146208447	0.000106421

chr22	46497766	46507175	-1.144533411	0.005056484
chr15	31546526	31573312	-1.1437809	7.76E-06
chr7	1568954	1574711	-1.142482389	0.000235901
chr8	134335470	134391007	-1.136072787	0.002367042
chr6	35691386	35701797	-1.134733443	6.62E-05
chr1	207098037	207154650	-1.131390166	0.003585078
chr11	2922397	2926733	-1.127961438	0.000137631
chr9	14309511	14323573	-1.125174648	0.002250256
chr20	56104107	56204341	-1.122036256	0.00336621
chr20	56229753	56295325	-1.118689646	0.004286441
chr19	49178577	49202140	-1.111287415	8.88E-05
chr17	73529524	73542716	-1.109659761	0.008782904
chr21	44858977	44873702	-1.109649262	0.001078597
chr9	2014195	2023271	-1.108875373	7.58E-06
chr14	105944150	105959031	-1.106254332	0.002882295
chr19	11353793	11380147	-1.104940631	1.87E-06
chr8	81397760	81421513	-1.104026822	0.000173483
chr8	145724875	145736551	-1.102944841	0.000375006
chr10	135156522	135179866	-1.10253336	0.004821712
chr19	45220249	45288652	-1.101828316	0.000358198
chr1	155161643	155165270	-1.101255846	0.006195042
chr11	2531969	2557416	-1.099006162	0.000600571
chr10	112562258	112639452	-1.095353097	0.00213485
chr16	88443224	88621560	-1.094897966	0.002762185
chr17	10676053	10719009	-1.094674354	0.004648038
chr11	606195	622242	-1.094121442	5.99E-05
chr5	172327125	172346151	-1.093982888	2.61E-05
chr22	50962309	50971267	-1.093835024	0.003064404
chr6	168413323	168503148	-1.09366734	2.41E-05
chr8	145043674	145052590	-1.093621486	0.000623648
chr17	57442508	57459901	-1.092540175	0.003262118
chr1	228349132	228354871	-1.091068244	0.000204448
chr6	89788647	89793074	-1.089729814	8.93E-09
chr2	28542414	28677214	-1.088175117	9.13E-05
chr22	46406761	46411260	-1.088019864	0.000118634
chr1	65530848	65534992	-1.086156021	0.000454408
chr13	41237892	41242018	-1.08574238	0.000736638
chr1	37936562	37964770	-1.084054544	0.000176254
chr1	20796697	20823686	-1.080299094	0.004959933
chr8	61811816	61840052	-1.080121891	4.15E-05
chr12	106620916	106644657	-1.079291391	3.69E-05
chr14	103367432	103415466	-1.07815608	2.61E-05
chr3	169755228	169768436	-1.072797034	7.41E-06
chr14	100569955	100660583	-1.069741409	0.000293944
chr2	208027703	208032208	-1.068574986	0.00013338

chr5	149941195	150017647	-1.064230609	5.23E-06
chr4	124315353	124341272	-1.055196918	0.001277395
chr9	139739767	139758297	-1.045706755	4.37E-05
chr4	141157126	141174915	-1.044484983	0.00687941
chr1	226029826	226110273	-1.037503403	7.21E-06
chr9	139683661	139691005	-1.034391734	9.92E-05
chr5	32298136	32333194	-1.033402799	0.003793373
chr16	57118390	57188393	-1.032752273	2.17E-05
chr2	242280263	242316360	-1.030467192	0.002928121
chr1	48150864	48195520	-1.024145926	2.27E-05
chr19	39887706	39902964	-1.023371562	0.000581726
chr2	238380339	238457019	-1.019570375	0.00548691
chr12	57519739	57575138	-1.017545226	0.000325957
chr2	241895868	241951777	-1.014868666	0.004083038
chr20	62150329	62155053	-1.010226977	0.000818134
chr19	49114785	49123342	-1.009885182	0.000928071
chr19	17875523	17914613	-1.007297164	9.04E-05
chr10	98337456	98394081	1.007035335	0.002355826
chr14	91817703	91881574	1.054473572	0.001319197
chr2	242784751	242844988	1.055869807	0.000500672
chr6	30691362	30762214	1.06354511	0.001788619
chr8	27189458	27266603	1.105089489	0.006499779
chr1	839207	879441	1.23821519	0.008747137
chr4	1492816	1584306	1.253051461	0.003244989
chr18	3583274	3626174	1.292623519	4.47E-05
chr7	130567798	130646840	1.301600783	0.000842286
chr12	46761555	46797433	1.368447694	0.00332708
chr17	48176137	48195174	1.393480425	0.000480532
chr17	17795500	17864058	1.41147405	0.007475802

Supplementary Table 5: Gene signature downstream of tumor-enriched SEs. Genes are mRNA up-regulated in primary CRCs over CCLE cell lines.

PTP4A3
IFITM2
SERPINA1
TMEM176B
COL16A1
C10orf99
PDZK1IP1
NOS2
SLC7A10
TMEM82
FXYD3
SHC2
GADD45G
CEACAM6
TMEM220
SOCS1
NR3C2
CYP27A1
ATP2A3
PALM
HSD11B2
FKBP5
PADI2
LRRC36
IFITM1
KIF25
PDE4C
SMPD3
MATK
TST
SMIM5
MAPK3
AMN
LCN12
CCDC183
CAMK2N1
KLF4

Supplementary Table 6: Super-enhancers up-regulated in primary CRC over patient-matched normal tissue, and also up-regulated in primary specimens over CRC cell lines.

	'start'	'end'
chr11	312970	318010
chr8	142422272	142429622
chr19	950367	958117
chr1	47644434	47659486
chr17	74485442	74490614
chr8	142203985	142256046
chr19	923608	929931
chr20	56229753	56295325
chr2	208027703	208032208

Supplementary Table 7: Juxtaposition of primary CRC cases with mutational status and *PDZK1IP1* SE status. All patients for which data are available are shown.

Patient	PDZK1IP1 SE	Gene	Mutation	Variant_Classif	Variant Allele Frequency
18481T	Yes	PRDM2	c.4027G>A	Missense_Muta	0.150344828
18481T	Yes	SZT2	c.2138del	Frame_Shift_D	0.383838384
18481T	Yes	CACNA1S	c.5012G>A	Missense_Muta	0.182072829
18481T	Yes	KISS1	c.225del	Frame_Shift_D	0.41025641
18481T	Yes	PIK3C2B	c.2698C>T	Missense_Muta	0.399463807
18481T	Yes	C2orf71	c.3839C>T	Missense_Muta	0.2
18481T	Yes	CASP8	c.1557del	Frame_Shift_D	0.350308642
18481T	Yes	CASP8	c.1586T>C	Missense_Muta	0.396258503
18481T	Yes	TGFBR2	c.644G>A	Missense_Muta	0.351687389
18481T	Yes	TGFBR2	c.1043T>C	Missense_Muta	0.364628821
18481T	Yes	PBRM1	c.1961A>G	Missense_Muta	0.189873418
18481T	Yes	PBRM1	c.518T>C	Missense_Muta	0.390728477
18481T	Yes	ATR	c.7375C>T	Missense_Muta	0.155555556
18481T	Yes	TET2	c.371A>G	Missense_Muta	0.179447853
18481T	Yes	TET2	c.4354C>T	Nonsense_Muta	0.377919321
18481T	Yes	TET2	c.4871A>G	Missense_Muta	0.208805031
18481T	Yes	FAT1	c.12608G>A	Missense_Muta	0.376744186
18481T	Yes	FAT1	c.7366G>A	Missense_Muta	0.352859135
18481T	Yes	ARID1B	c.1988A>G	Missense_Muta	0.19895288
18481T	Yes	EGFR	c.2029C>T	Missense_Muta	0.193548387
18481T	Yes	EGFR	c.2930G>A	Missense_Muta	0.393333333
18481T	Yes	MET	c.40C>T	Missense_Muta	0.418390805
18481T	Yes	KMT2C	c.7238C>T	Missense_Muta	0.18814433
18481T	Yes	WRN	c.2743T>C	Missense_Muta	0.181081081
18481T	Yes	ANK1	c.1231G>A	Missense_Muta	0.420454545

18481T	Yes	SMARCA2	c.980G>A	Missense_Muta	0.333333333
18481T	Yes	GATA3	c.1134del	Frame_Shift_D	0.408333333
18481T	Yes	RET	c.169C>T	Missense_Muta	0.343023256
18481T	Yes	ADK	c.731C>T	Missense_Muta	0.152777778
18481T	Yes	KAT6B	c.4121G>A	Missense_Muta	0.37628866
18481T	Yes	TCF7L2	c.1485del	Frame_Shift_D	0.452830189
18481T	Yes	FGFR2	c.181C>T	Missense_Muta	0.368512111
18481T	Yes	DRD4	c.763del	Frame_Shift_D	0.344827586
18481T	Yes	FAT3	c.971A>G	Missense_Muta	0.158798283
18481T	Yes	KMT2A	c.5338del	Frame_Shift_D	0.179824561
18481T	Yes	CHD4	c.2402T>C	Missense_Muta	0.338461538
18481T	Yes	KMT2D	c.9417del	Frame_Shift_D	0.401574803
18481T	Yes	KMT2D	c.7061del	Frame_Shift_D	0.25
18481T	Yes	KMT2D	c.1300del	Frame_Shift_D	0.227272727
18481T	Yes	ERBB3	c.2033G>A	Missense_Muta	0.332129964
18481T	Yes	POLE	c.1309G>A	Missense_Muta	0.370481928
18481T	Yes	POLE	c.1280C>T	Missense_Muta	0.341397849
18481T	Yes	BRCA2	c.2957del	Frame_Shift_D	0.33611691
18481T	Yes	RB1	c.971T>A	Missense_Muta	0.333333333
18481T	Yes	PCDH8	c.1975C>T	Missense_Muta	0.36
18481T	Yes	PCDH8	c.1432C>T	Missense_Muta	0.413043478
18481T	Yes	PCDH8	c.1397C>T	Missense_Muta	0.295454545
18481T	Yes	PCDH8	c.277G>A	Missense_Muta	0.371273713
18481T	Yes	PCDH17	c.1898A>G	Missense_Muta	0.394160584
18481T	Yes	PCDH17	c.2714A>T	Missense_Muta	0.364
18481T	Yes	DICER1	c.4458del	Frame_Shift_D	0.368088468
18481T	Yes	DICER1	c.1859A>G	Missense_Muta	0.123214286
18481T	Yes	AXIN1	c.1565C>T	Missense_Muta	0.28
18481T	Yes	PIGQ	c.1679G>A	Missense_Muta	0.142857143
18481T	Yes	TSC2	c.1723C>A	Missense_Muta	0.333333333
18481T	Yes	PLEKHG4	c.1574del	Frame_Shift_D	0.214285714
18481T	Yes	PLEKHG4	c.1575C>G	Missense_Muta	0.214285714
18481T	Yes	C16orf86	c.461A>G	Missense_Muta	0.191780822
18481T	Yes	CD68	c.84del	Frame_Shift_D	0.368181818
18481T	Yes	TP53	c.998G>A	Missense_Muta	0.355102041
18481T	Yes	NF1	c.8158A>G	Missense_Muta	0.310344828
18481T	Yes	ERBB2	c.2516T>C	Missense_Muta	0.169934641
18481T	Yes	KEAP1	c.1639G>A	Missense_Muta	0.35978836
18481T	Yes	DAND5	c.37C>T	Missense_Muta	0.222222222
18481T	Yes	FCHO1	c.2354T>C	Missense_Muta	0.436170213
18481T	Yes	JAK3	c.911A>T	Missense_Muta	0.48
18481T	Yes	PIK3R2	c.862C>T	Missense_Muta	0.150943396
18481T	Yes	KMT2B	c.1833G>A	Nonsense_Muta	0.443820225
18481T	Yes	ASXL1	c.2993C>T	Missense_Muta	0.34765625
18481T	Yes	SRC	c.659G>A	Missense_Muta	0.356321839

18481T	Yes	SRC	c.800G>A	Missense_Muta	0.473684211
18481T	Yes	SMC1A	c.2701G>A	Missense_Muta	0.373608903
18481T	Yes	SMC1A	c.1732-1G>T	Splice_Site	0.363914373
18481T	Yes	SMC1A	c.401T>C	Missense_Muta	0.320486815
18481T	Yes	AR	c.2086G>A	Missense_Muta	0.349887133
18481T	Yes	STAG2	c.3584G>A	Missense_Muta	0.323943662
17627T	Yes	APC	c.3733A>T	Nonsense_Muta	0.395480226
17627T	Yes	ATM	c.6679C>T	Missense_Muta	0.310559006
17627T	Yes	SETBP1	c.4511C>T	Missense_Muta	0.363636364
17627T	Yes	SMAD2	c.1272_1279du	Frame_Shift_In	0.643410853
17627T	Yes	NXNL1	c.376C>G	Missense_Muta	0.142857143
18946T	No	NRAS	c.34G>T	Missense_Muta	0.726744186
18946T	No	APC	c.1495C>T	Nonsense_Muta	0.441201717
18946T	No	APC	c.4339del	Frame_Shift_D	0.459482038
18946T	No	SMC3	c.212C>T	Missense_Muta	0.41686747
18946T	No	ATM	c.2084T>C	Missense_Muta	0.45041816
18946T	No	ATM	c.9022C>T	Missense_Muta	0.443911793
18946T	No	CHD4	c.4216C>T	Missense_Muta	0.419981499
18946T	No	FLT3	c.106G>T	Missense_Muta	0.620921802
18946T	No	ERBB2	c.2033G>A	Missense_Muta	0.550724638
19395T	Yes	PIK3CA	c.323G>A	Missense_Muta	0.376528117
19395T	Yes	PIK3CA	c.1634A>C	Missense_Muta	0.251655629
19395T	Yes	APC	c.2336dup	Frame_Shift_In	0.39544808
19395T	Yes	APC	c.4459_4460ins	Frame_Shift_In	0.393899204
19395T	Yes	APC	c.4460C>A	Missense_Muta	0.394946809
19395T	Yes	BRAF	c.1799T>A	Missense_Muta	0.269349845
19395T	Yes	FAT3	c.2762C>A	Missense_Muta	0.3671875
19395T	Yes	FAT3	c.4718C>T	Missense_Muta	0.352380952
19395T	Yes	KMT2D	c.2021C>T	Missense_Muta	0.307692308
19395T	Yes	AGRP	c.262G>A	Missense_Muta	0.486631016
19395T	Yes	TP53	c.743G>A	Missense_Muta	0.6125
19395T	Yes	SOX9	c.736C>T	Nonsense_Muta	0.363888889
19395T	Yes	SMAD2	c.1389C>A	Nonsense_Muta	0.379496403
19395T	Yes	SMAD2	c.904T>C	Missense_Muta	0.380514706
19395T	Yes	KMT2B	c.2732C>T	Missense_Muta	0.41509434
19442T	Yes	PRDM2	c.2933C>T	Missense_Muta	0.121718377
19442T	Yes	PRDM2	c.4184G>A	Missense_Muta	0.170694864
19442T	Yes	ARID1A	c.2402dup	Frame_Shift_In	0.102857143
19442T	Yes	IGFN1	c.10258G>A	Missense_Muta	0.175
19442T	Yes	FH	c.535G>A	Missense_Muta	0.176870748
19442T	Yes	ALK	c.474dup	Frame_Shift_In	0.190082645
19442T	Yes	ALS2	c.4136_4138del	In_Frame_Del	0.178907721
19442T	Yes	ERBB4	c.3817C>T	Missense_Muta	0.151821862
19442T	Yes	BAP1	c.1628G>A	Missense_Muta	0.154185022
19442T	Yes	PBRM1	c.1601G>A	Missense_Muta	0.209191759

19442T	Yes	PIK3CA	c.331A>G	Missense_Muta	0.180987203
19442T	Yes	FGFR3	c.1075C>T	Nonsense_Muta	0.144230769
19442T	Yes	TET2	c.4317dup	Frame_Shift_In	0.193014706
19442T	Yes	TET2	c.5597C>T	Missense_Muta	0.196048632
19442T	Yes	FAT1	c.9857C>G	Missense_Muta	0.173780488
19442T	Yes	FAT1	c.4184G>A	Missense_Muta	0.121510673
19442T	Yes	FAT1	c.2833G>A	Missense_Muta	0.201787995
19442T	Yes	APC	c.4666dup	Frame_Shift_In	0.210526316
19442T	Yes	KMT2C	c.13940dup	Frame_Shift_In	0.166276347
19442T	Yes	ACTR3B	c.829C>A	Missense_Muta	0.197740113
19442T	Yes	FGFR1	c.1588G>A	Missense_Muta	0.177446103
19442T	Yes	KAT6A	c.76C>T	Missense_Muta	0.180327869
19442T	Yes	SMARCA2	c.4354dup	Frame_Shift_In	0.243589744
19442T	Yes	FAM166B	c.470C>T	Missense_Muta	0.5625
19442T	Yes	PTCH1	c.3793G>A	Missense_Muta	0.155555556
19442T	Yes	PTCH1	c.3622G>A	Missense_Muta	0.185714286
19442T	Yes	TSC1	c.1257del	Frame_Shift_D	0.217687075
19442T	Yes	NOTCH1	c.4828G>A	Missense_Muta	0.203389831
19442T	Yes	KAT6B	c.4523del	Frame_Shift_D	0.195439739
19442T	Yes	OR2D3	c.234dup	Frame_Shift_In	0.19524618
19442T	Yes	NRXN2	c.2011C>T	Missense_Muta	0.223300971
19442T	Yes	EHD1	c.490C>T	Missense_Muta	0.18694362
19442T	Yes	FAT3	c.3998A>G	Missense_Muta	0.244705882
19442T	Yes	FAT3	c.6679C>T	Missense_Muta	0.12424608
19442T	Yes	KMT2D	c.7109G>A	Missense_Muta	0.216216216
19442T	Yes	KMT2D	c.7061del	Frame_Shift_D	0.351351351
19442T	Yes	KMT2D	c.4841G>A	Missense_Muta	0.174563591
19442T	Yes	KMT2D	c.2993del	Frame_Shift_D	0.170731707
19442T	Yes	NCOR2	c.6923C>T	Missense_Muta	0.141304348
19442T	Yes	RB1	c.1478T>A	Missense_Muta	0.149797571
19442T	Yes	PCDH17	c.2579C>T	Missense_Muta	0.202925046
19442T	Yes	AKT1	c.745C>T	Missense_Muta	0.285714286
19442T	Yes	BLM	c.2268del	Frame_Shift_D	0.213296399
19442T	Yes	AXIN1	c.1523del	Frame_Shift_D	0.407407407
19442T	Yes	CREBBP	c.2469G>T	Missense_Muta	0.111111111
19442T	Yes	FANCA	c.623C>T	Missense_Muta	0.18018018
19442T	Yes	MAP2K2	c.329C>T	Missense_Muta	0.209923664
19442T	Yes	SMARCA4	c.2714G>A	Missense_Muta	0.166240409
19442T	Yes	SMARCA4	c.4336C>G	Missense_Muta	0.156593407
19442T	Yes	OR7A17	c.113C>T	Missense_Muta	0.156097561
19442T	Yes	SLC27A1	c.1259G>A	Missense_Muta	0.183431953
19442T	Yes	PIK3R2	c.107C>T	Missense_Muta	0.2
19442T	Yes	SRC	c.1259C>T	Missense_Muta	0.174496644
19442T	Yes	RUNX1	c.1108G>T	Missense_Muta	0.193548387
19442T	Yes	EP300	c.5375A>C	Missense_Muta	0.177570093

19442T	Yes	SMC1A	c.2695G>A	Missense_Muta	0.203262233
19442T	Yes	AMER1	c.3009A>G	Missense_Muta	0.197101449
19442T	Yes	ATRX	c.3682C>T	Missense_Muta	0.169724771
19442T	Yes	PLXNB3	c.1970G>A	Missense_Muta	0.11627907
17787T	No	TP53	c.701A>G	Missense_Muta	0.618421053
17787T	No	KEAP1	c.1133G>A	Missense_Muta	0.663793103
18081T	Yes	APC	c.646C>T	Nonsense_Muta	0.635416667
18081T	Yes	PDE6A	c.2302G>A	Missense_Muta	0.333333333
18081T	Yes	POLE	c.5573G>A	Missense_Muta	0.381294964
19106T	Yes	PRDM16	c.1898C>T	Missense_Muta	0.951612903
19106T	Yes	PRDM16	c.3369_3371du	In_Frame_Ins	0.92
19106T	Yes	IDH1	c.532G>A	Missense_Muta	0.302449414
19106T	Yes	MLH1	c.655A>G	Missense_Muta	0.372986369
19106T	Yes	TACC3	c.427G>A	Missense_Muta	0.358422939
19106T	Yes	UGT2B7	c.801_802delin	Missense_Muta	0.319034853
19106T	Yes	FAT1	c.8535G>A	Missense_Muta	0.355665025
19106T	Yes	PNPLA1	c.1268C>A	Missense_Muta	0.914285714
19106T	Yes	TSC1	c.1079C>A	Missense_Muta	0.337777778
19106T	Yes	ATE1	c.499C>T	Missense_Muta	0.378723404
19106T	Yes	TACC2	c.2488C>T	Missense_Muta	0.363043478
19106T	Yes	FAT3	c.8983C>A	Missense_Muta	0.290087464
19106T	Yes	TECTA	c.1111A>G	Missense_Muta	0.33492823
19106T	Yes	TP53BP1	c.1074C>G	Missense_Muta	0.949874687
19106T	Yes	MPDU1	c.685G>A	Missense_Muta	0.969465649
19106T	Yes	TP53	c.326dup	Frame_Shift_In	0.910714286
19282T	No	SMO	c.2081del	Frame_Shift_D	0.117021277
18607T	Yes	ADCY3	c.2387G>A	Missense_Muta	0.167630058
18607T	Yes	MSH6	c.2079del	Frame_Shift_D	0.16286645
18607T	Yes	CASP8	c.1596del	Frame_Shift_D	0.121794872
18607T	Yes	PIK3CA	c.3140A>G	Missense_Muta	0.159292035
18607T	Yes	TET2	c.3157C>T	Nonsense_Muta	0.166666667
18607T	Yes	FAT1	c.13717G>A	Missense_Muta	0.153439153
18607T	Yes	EGFR	c.1282G>A	Missense_Muta	0.174208145
18607T	Yes	PVRIG	c.892del	Frame_Shift_D	0.115942029
18607T	Yes	BRAF	c.1799T>A	Missense_Muta	0.165254237
18607T	Yes	WT1	c.496G>A	Missense_Muta	0.142857143
18607T	Yes	CCDC73	c.1341del	Frame_Shift_D	0.363636364
18607T	Yes	KMT2D	c.1940del	Frame_Shift_D	0.24
18607T	Yes	BRCA2	c.10248del	Frame_Shift_D	0.136842105
18607T	Yes	C14orf79	c.83C>A	Missense_Muta	0.171929825
18607T	Yes	IDH2	c.435del	Frame_Shift_D	0.184466019
18607T	Yes	SPNS1	c.1133G>A	Missense_Muta	0.217391304
18607T	Yes	TRADD	c.286C>T	Missense_Muta	0.285714286
18607T	Yes	ERBB2	c.2446C>T	Missense_Muta	0.154589372
18607T	Yes	ERBB2	c.3235G>A	Missense_Muta	0.159663866

18607T	Yes	SMARCE1	c.903G>T	Missense_Muta	0.157258065
18607T	Yes	SOX9	c.984_1036del	Frame_Shift_D	0.121428571
18607T	Yes	RGL3	c.925G>A	Missense_Muta	0.141025641
18607T	Yes	CHEK2	c.1081C>T	Missense_Muta	0.158798283
18810T	Yes	PIK3CA	c.1633G>A	Missense_Muta	0.406779661
18810T	Yes	FAT1	c.7699C>T	Missense_Muta	0.437694704
18810T	Yes	APC	c.1958+2T>G	Splice_Site	0.42019544
18810T	Yes	APC	c.4464_4467del	Frame_Shift_D	0.382075472
18810T	Yes	KRAS	c.38G>A	Missense_Muta	0.426273458
18810T	Yes	FZD10	c.1127C>T	Missense_Muta	0.446327684
18810T	Yes	SMAD2	c.961C>T	Nonsense_Muta	0.112676056
18810T	Yes	SMAD4	c.1065C>A	Missense_Muta	0.535947712
18739T	Yes	PIK3CA	c.1624G>A	Missense_Muta	0.290187891
18739T	Yes	FBXW7	c.941del	Frame_Shift_D	0.367454068
18739T	Yes	APC	c.4166_4167ins	Frame_Shift_In	0.430107527
18739T	Yes	KRAS	c.35G>A	Missense_Muta	0.486166008
18739T	Yes	TP53	c.743G>A	Missense_Muta	0.696296296
19441T	Yes	APC	c.2711_2712del	Frame_Shift_D	0.392703863
19441T	Yes	NOTCH1	c.4864C>T	Missense_Muta	0.465116279
19441T	Yes	TP53	c.880G>T	Nonsense_Muta	0.361313869
19441T	Yes	FAM129C	c.1967G>A	Missense_Muta	0.380614657
19342T	Yes	SMARCA2	c.2363G>A	Nonsense_Muta	0.339756592
19342T	Yes	TP53	c.853G>T	Nonsense_Muta	0.611515553
19342T	Yes	TP53	c.659A>G	Missense_Muta	0.231943629
19342T	Yes	SUZ12	c.1105T>C	Missense_Muta	0.601747815
19342T	Yes	SMAD4	c.1594_1596del	In_Frame_Del	0.573435504

Supplementary Table 8: Juxtaposition of primary CRC cases with patient demographics and *PDZK1IP1* SE status.

Samples	Has PDZK1IP1	Sex	Age	Colon Anatomy
18481T	Yes	no data		
17753T	Yes	Male	63	Sigmoid colon and rectum
18607T	Yes	Male	71	Terminal ileum appendix and ascending colon
18810T	Yes	Male	67	Terminal ileum and cecum
19441T	Yes	Male	71	Descending colon
19282T	No	Female	82	Terminal ileum and ascending colon
19106T	Yes	Male	74	Sigmoid colon and rectum
19342T	Yes	Male	53	Transverse colon
18739T	Yes	Male	63	Sigmoid colon
18946T	No	Male	83	Descending colon
18081T	Yes	Male	62	Sigmoid colon and rectum

19442T	Yes	Female	61	Terminal ileum appendix and ascending colon
19395T	Yes	Male	61	Terminal ileum, cecum, appendix, and ascending colon
17627T	Yes	Male	64	Sigmoid colon
17787T	No	Male	67	Descending and sigmoid colon

Supplementary Table 9: Juxtaposition of primary CRC cases with pathological features and *PDZK1IP1* SE status.

Samples	Has PDZK1IP1 super-enhancer?	Tumor Staging	Primary Tumor Description
18481T	Yes		No data
17753T	Yes	pT4aN2a	Invasive, moderately differentiated adenocarcinoma with mucinous features. The tumor invades through the wall to the serosal surface with acute inflammatory reaction. Lymphovascular and perineural invasion identified. Extramural large vessel invasion identified.
18607T	Yes	pT3N1a	Invasive poorly differentiated adenocarcinoma with medullary feature. Invades into subserosal soft tissue. Perineural invasion also present. Extensive lymphatic invasion seen. IHC shows MLH1 and PMS2 loss, possible MSI.

18810T	Yes	pT1N0	Invasive moderately differentiated adenocarcinoma. The tumor invades the submucosa. Foci suspicious for lymphovascular invasion are identified. Perineural invasion is not identified.
19441T	Yes	pT4aN0	Invasive moderately differentiated adenocarcinoma extending through the serosal surface. Perineural and lymphovascular space invasion are presnet. Peritumoral abscess with foreign body giant cell reaction. Large extramural vein invasion.
19282T	No	no data	Poorly differentiated adenocarcinoma. No adenomatous component is found to confirm primary colonic origin. The tumor is almost entirely intralymphatic and there is focal intramural venous invasion. The tumor is CDX2 negative which is uncommon for primary CRC but not exlcusionary. Additional immunostains are negative for primary breast, GYN, or pulmonary origin.

19106T	Yes	pT3N1a	Invasive moderately differentiated adenocarcinoma with invasion to the pericolorectal adipose tissue. Lymphovascular and neural invasions present.
19342T	Yes	pT2N0	Moderately differentiated mucinous adenocarcinoma with invasion into the muscularis propria. India ink tattoo noted.
18739T	Yes	pT4aN0	Moderately differentiated adenocarcinoma with transmural invasion to the visceral peritoneum. Focal high-grade tumor budding and extramural large vein invasion are identified.
18946T	No	pT4N0	Invasive, moderately differentiated adenocarcinoma extending to the serosal surface. Submucosal ink tattoo. Lymphovascular or perineural space invasion is not identified. Exuberant fibrohistiocytic reaction of the subserosal soft tissue.
18081T	Yes	pT3N0	Invasive moderately differentiated adenocarcinoma. The tumor invades the mucscularis propria and into subserosal soft tissue. Tumor budding.

19442T	Yes	pT4aN0	Invasive moderately differentiated mucinous adenocarcinoma. Extended through the serosal surface into the visceral peritoneum with associated fibroinflammatory response. Lymphovascular or perineural space invasion is not identified. No evidence of large extramural vein invasion.
19395T	Yes		Moderately differentiated mucinous adenocarcinoma. The tumor invades through muscularis propria into pericolic fat. Vascular invasion is not identified. Perineural invasion is not identified.
17627T	Yes	no data	No data
17787T	No	pT4bN2b	Moderate to poorly differentiated carcinoma with mixed morphology. Extensive lymphovascular and perineural invasion are noted. Extramural large vessel invasion is identified. Background colon with mildly active chronic colitics and melanosis coli. Long history with quiescent ulcerative colitis.

Supplementary Table 10: H3K27ac ChIP-seq metrics.

17627T: 74,355 called peaks stitched to 17,726 enhancers.

17627N: 75,466 called peaks stitched to 21,636 enhancers.

17753T: 59,688 called peaks stitched to 13,726 enhancers.

17753N: 175,589 called peaks stitched to 5,392 enhancers.

17787T: 76,011 called peaks stitched to 20,341 enhancers.

17787N: 66,728 called peaks stitched to 17,567 enhancers.

18081T: 54,343 called peaks stitched to 15,075 enhancers.

18081N: 52,082 called peaks stitched to 14,110 enhancers.

18481T: 29,640 called peaks stitched to 7,921 enhancers.

18481N: 75,102 called peaks stitched to 24,268 enhancers.

18607T: 47,801 called peaks stitched to 24,704 enhancers.

18607N: 80,274 called peaks stitched to 19,847 enhancers.

18739T: 56,757 called peaks stitched to 15,073 enhancers.

18739N: 34,545 called peaks stitched to 5,692 enhancers.

18946T: 51,652 called peaks stitched to 19,214 enhancers.

18946N: 27,010 called peaks stitched to 15,655 enhancers.

18810T: 72,790 called peaks stitched to 13,804 enhancers.

18810N: 57,932 called peaks stitched to 6,217 enhancers.

19106T: 58,973 called peaks stitched to 16,857 enhancers.

19106N: 61,476 called peaks stitched to 15,331 enhancers.

19282T: 63,909 called peaks stitched to 17,020 enhancers.

19282N: 59,106 called peaks stitched to 15,434 enhancers.

19342T: 53,155 called peaks stitched to 14,616 enhancers.

19342N: 52,206 called peaks stitched to 13,616 enhancers.

19395T: 63,693 called peaks stitched to 18,157 enhancers.

19395N: 47,387 called peaks to 13,483 enhancers.

19441T: 70,089 called peaks stitched to 18,884 enhancers.

19441N: 63,324 called peaks stitched to 16,074 enhancers.

19442T: 62,613 called peaks stitched to 16,760 enhancers.

19442N: 77,621 called peaks stitched to 19,239 enhancers.

HT29 cells: 43,055 called peaks stitched to 10.784 enhancers.

HT29 xenograft tumor rep. 1: 47,940 called peaks stitched to 13,097 enhancers.

HT29 xenograft tumor rep. 2: 61,713 called peaks stitched to 18,869 enhancers.

HT29 xenograft tumors rep. 3: 27,347 called peaks stitched to 7,049 enhancers.

HT29 TNF-alpha, IFN-gamma, and IL-6 treated cells: 60,741 called peaks stitched to 15,139 enhancers.

Supplementary Table 11: Consensus molecular subtypes of primary CRCs and PDZK1IP1 expression by RNA-seq.

Primary CRC	prediction	P value	FDR	PDZK1IP1 mRNA expression (TPM)
X17243T	CMS2	0.001	0.00133333	138.915
X17547T	CMS4	0.001	0.00133333	1.31922
X17561T	CMS1	0.002997	0.00368862	118.964
X17627T	CMS3	0.001	0.00133333	96.4203
X17753T	CMS1	0.00499501	0.00570858	224.632
X17787T	CMS4	0.001	0.00133333	14.4398
X18081T	CMS2	0.001	0.00133333	130.041
X18481T	CMS3	0.001	0.00133333	61.7672
X18607T	CMS1	0.001	0.00133333	146.586
X18810T	CMS3	0.001	0.00133333	90.4694
X19057T	CMS4	0.001	0.00133333	1.48811
X19107T	CMS2	0.001	0.00133333	67.1396
X19282T	CMS4	0.001	0.00133333	0.865068
X19342T	NA	0.94205794	0.94205794	130.711
X19395T	NA	0.44155844	0.47099567	70.1376

Supplementary Table 12: H3K27ac and H3K4me1 calling of the PDZK1IP1 super-enhancer using ROSE.

Sample	H3K27ac?	H3K4me1?
N1 (normal)	No	No
N3 (normal)	No	No
N4 (normal)	No	No
N5 (normal)	No	No
N6 (normal)	No	No
N8 (normal)	No	No
N11 (normal)	No	No
N12 (normal)	No	No
N13 (normal)	No	No
N15 (normal)	No	No
N17 (normal)	No	No
N18 (normal)	No	No
N20 (normal)	No	No

T1 (tumor)	Yes	No
T3 (tumor)	No	No
T4 (tumor)	Yes	No
T5 (tumor)	No	No
T7 (tumor)	No	No
T8 (tumor)	No	No
T11 (tumor)	No	No
T12 (tumor)	No	No
T13 (tumor)	No	No
T14 (tumor)	Yes	No
T15 (tumor)	Yes	Yes
T16 (tumor)	No	No
T17 (tumor)	Yes	No
T18 (tumor)	Yes	Yes
