# Supplementary material for: A local tumor microenvironment acquired super-enhancer induces an oncogenic driver in colorectal carcinoma
Source: Nat Commun. 2022 Oct 17;13:6041. doi: 10.1038/s41467-022-33377-8 (PMC9576746; doi:10.1038/s41467-022-33377-8)
Supplement: Supplementary file 3 — Reporting Summary [file 41467_2022_33377_MOESM3_ESM.pdf]

nature research

Corresponding author(s): Ramon Parsons

Last updated by author(s): Aug 3, 2022

Reporting Summary

Nature Research wishes to improve the reproducibility of the work that we publish. This form provides structure for consistency and transparency in reporting. For further information on Nature Research policies, see our Editorial Policies and the Editorial Policy Checklist.

Statistics

For all statistical analyses, confirm that the following items are present in the figure legend, table legend, main text, or Methods section.

n/a	Confirmed	
☐	☒	The exact sample size (*n*) for each experimental group/condition, given as a discrete number and unit of measurement
☐	☒	A statement on whether measurements were taken from distinct samples or whether the same sample was measured repeatedly
☐	☒	The statistical test(s) used AND whether they are one- or two-sided<br>*Only common tests should be described solely by name; describe more complex techniques in the Methods section.*
☒	☐	A description of all covariates tested
☒	☐	A description of any assumptions or corrections, such as tests of normality and adjustment for multiple comparisons
☐	☒	A full description of the statistical parameters including central tendency (e.g. means) or other basic estimates (e.g. regression coefficient) AND variation (e.g. standard deviation) or associated estimates of uncertainty (e.g. confidence intervals)
☐	☒	For null hypothesis testing, the test statistic (e.g. *F*, *t*, *r*) with confidence intervals, effect sizes, degrees of freedom and *P* value noted<br>*Give P values as exact values whenever suitable.*
☒	☐	For Bayesian analysis, information on the choice of priors and Markov chain Monte Carlo settings
☒	☐	For hierarchical and complex designs, identification of the appropriate level for tests and full reporting of outcomes
☐	☒	Estimates of effect sizes (e.g. Cohen's *d*, Pearson's *r*), indicating how they were calculated

Our web collection on statistics for biologists contains articles on many of the points above.

Software and code

Policy information about availability of computer code

Data collection	None.
Data analysis	The following software was used for the analysis of ChIP-seq data: bedtools v2.21.0, deeptools v3.1.2, macs2 v2.1.0.20140616, Rank Ordering of Super-Enhancers (ROSE) http://younglab.wi.mit.edu/super_enhancer_code.html, The Transcription Factor Affinity Prediction (TRAP) http://trap.molgen.mpg.de/cgi-bin/home.cgi, samtools v1.10, Integrated Genomics Viewer (IGV browser) v.2.9.4, FastQC v0.11.8, LiftOver tool on UCSC Genome Browser (used online, no Version number available). The following software was used for the analysis of RNA-seq data: salmon v0.9.1, DESEQ2 v3.12, R version 4.0, GSEA v4.10. The following software was used for statistical analyses: GraphPad Version 7, Microsoft Excel for Mac Version 16. Immunoblot protein quantification was performed using ImageJ Version 1.53.

For manuscripts utilizing custom algorithms or software that are central to the research but not yet described in published literature, software must be made available to editors and reviewers. We strongly encourage code deposition in a community repository (e.g. GitHub). See the Nature Research guidelines for submitting code & software for further information.

Data

Policy information about availability of data

All manuscripts must include a data availability statement. This statement should provide the following information, where applicable:

- Accession codes, unique identifiers, or web links for publicly available datasets
- A list of figures that have associated raw data
- A description of any restrictions on data availability

H3K27ac ChIP-seq and RNA-seq datasets performed in this study are deposited at the NCBI Gene Expression Omnibus under the accession GSE166254. H3K27ac ChIP-seq datasets previously performed in FAP adenomas and normal colon crypts were accessed from the Gene Expression Omnibus under the accession GSE7773717. Broad Institute Cancer Cell Line Encyclopedia (CCLE) RNA-seq datasets for CRC cell lines were accessed from the Gene Expression Omnibus under the

accession PRJNA52338023. COLO205 in vitro and orthotopic tumor H3K27ac ChIP-seq datasets presented in fig. S2C-E, 3A-B were accessed under the accession GSE12618843. RELA ChIP-seq datasets from adipocytes were accessed under the accession GSM156673577; RELA ChIP-seq datasets from A549 cells were accessed under the accession GSM84787678; STAT1 ChIP-seq datasets from HeLa cells were accessed under the accession GSM38550579; STAT1 ChIP-seq datasets from monocytes were accessed under the accession GSM105701180; STAT3 ChIP-seq datasets from MDA-MB-468 cells were accessed under the accession GSM227800381; CTCF ChIP-seq from normal colon tissue was accessed from ENCODE82. Osteosarcoma H3K27ac ChIP-seq datasets accessed under accession GSE7423083; gastric cancer H3K27ac ChIP-seq datasets accessed under accession GSE76153 & GSE7589884; luminal breast cancer H3K27ac ChIP-seq datasets accessed at the ENA under accession PRJEB2275785; prostate cancer H3K27ac ChIP-seq datasets accessed under accession GSE9665286; GBM H3K27ac ChIP-seq datasets accessed under accession GSE11983487; chordoma H3K27ac ChIP-seq datasets accessed under accession GSE10979488; ccRCC H3K27ac ChIP-seq datasets accessed under accession GSE8609589; DLBCL H3K27ac ChIP-seq datasets accessed under accession GSE4666390. OncoLnc was used to generate Kaplan Meier curves for PDZK1IP1-high and -low mRNA expression cases based off TCGA data. TCGA public datasets used include ATAC-seq data and RNA-seq from all available CRC samples (listed as COAD for colon adenocarcinoma). Single-cell RNA-seq data from primary CRC and normal colon epithelium was accessed under the accession EGAS00001003779 and EGAS00001003769 from the European Genome-phenome Archive database31. Raw H3K27ac ChIP-seq sequencing data from primary CRC and matched human colon organoids, also referred to in the manuscript as the Milan cohort, were accessed under E-MTAB-8416, and raw RNA-seq sequencing data was accessed under E-MTAB-844830. Raw H3K27ac ChIP-seq sequencing data from commercially available human CRC cell lines were accessed under GSE96069, GSE73319, GSE126188, and GSE7773717,19,20,43. H3K27ac and H3K4me1 ChIP-seq datasets previously published were accessed under the accession GSE88945) 15. Hi-ChIP datasets previously published were accessed under the accession GSE133928; primary tumor samples MGH1904, MGH5328, MGH8416 from the original publication were used in this analysis16. The minimum dataset needed to interpret, verify, and extend the research as provided as Raw Source Data in the Supplementary Files; for larger datasets, please see NCBI Gene Expression Omnibus under the accession GSE166254. The following human genome accessions were used: The NCBI accession of the UCSC hg38 genome is GCA_000001405.15, and the UCSC hg19 genome is GCA_000001405.1. The following mouse genome accessions were used: GRCm38/mm10 assembly via UCSC under GCA_000001635.2.

Field-specific reporting

Please select the one below that is the best fit for your research. If you are not sure, read the appropriate sections before making your selection.

☒ Life sciences ☐ Behavioural & social sciences ☐ Ecological, evolutionary & environmental sciences

For a reference copy of the document with all sections, see nature.com/documents/nr-reporting-summary-flat.pdf

Life sciences study design

All studies must disclose on these points even when the disclosure is negative.

Sample size	No statistical analyses were performed to pre-determine sample size. Power analyses performed retroactively validate our 15 patient cohort saturated and was sufficiently powered for novel super-enhancer discovery. For all other experiments, a minimum of 3 biological replicates were used for the determination of statistical analysis in accordance with standard rigor and reproducibility practice guidelines by the NIH.
Data exclusions	No data were excluded in this study.
Replication	The majority of in vitro experiments were performed 3 or more times on independent samples, and all results were reproducible. Where experiments were performed once, the phenotypes were robust and validated using orthogonal methods and/or the same experiment performed in different cell lines. The exact n for each experiment is noted in the Figure Legends. Mouse studies were not replicated but included sufficient sample size to account for biological variability.
Randomization	For mouse studies, cages were allocated randomly. Randomization of samples into experimental groups did not apply for all other experiments (cell culture).
Blinding	Investigators were not blinded for the analysis of experiments as it was not feasible.

Reporting for specific materials, systems and methods

We require information from authors about some types of materials, experimental systems and methods used in many studies. Here, indicate whether each material, system or method listed is relevant to your study. If you are not sure if a list item applies to your research, read the appropriate section before selecting a response.

Materials & experimental systems

n/a	Involved in the study	
☐	☒	Antibodies
☐	☒	Eukaryotic cell lines
☒	☐	Palaeontology and archaeology
☐	☒	Animals and other organisms
☐	☒	Human research participants
☒	☐	Clinical data
☒	☐	Dual use research of concern

Methods

n/a	Involved in the study	
☐	☒	ChIP-seq
☒	☐	Flow cytometry
☒	☐	MRI-based neuroimaging

Antibodies

Antibodies used	The following primary antibodies were used for IHC: anti-CD11c (CST #97585), anti-CD68 (CST #97778), anti-perforin (CST #31647), and anti-Ly-6G (CST #87048). The following primary antibodies were used for immunoblot: anti-PDZK1IP1 (Sigma #HPA014907), anti-GAPDH (Cell Signaling Technologies, CST #2118), anti-V5 (CST #13202), anti-phosphorylated STAT3 at Y705 (CST #9145). H3K27ac ChIP-seq antibodies were purchased from abcam (#ab177178). Horse radish peroxidase conjugated secondary antibodies: mouse (Thermo #31432) and rabbit (Thermo #31460) both used at 1:2000 concentrations).
Validation	The following antibodies were validated by the Ramon Parsons laboratory using knockout or overexpression studies: anti-CD68 (CST #97778), anti-PDZK1IP1 (Sigma #HPA014907), anti-V5 (CST #13202). The following antibodies were validated by the manufacturer using knockout or overexpression studies: anti-Ly-6G (CST #87048), anti-CD68 (CST #97778), anti-V5 (CST #13202), anti-PDZK1IP1 (Sigma #HPA014907). The anti-phosphorylated STAT3 at Y705 (CST #9145) was validated using an inhibitor of STAT3 C188-9, and by the manufacturer via interferon stimulation of cell culture. The H3K27ac antibody was validated by the manufacturer through the use of histone deacetylase inhibitor Trichostatin A as well as a peptide array to assess for binding to other histone modifications.

Eukaryotic cell lines

Policy information about cell lines

Cell line source(s)	The following cell lines were used in this study: HT29, COLO205, DLD1, and HEK293T all purchased from ATCC.
Authentication	HT29 was authenticated using short tandem repeat (STR) profiling at the Mount Sinai Oncological Sciences Sequencing Core Facility. HEK293T, COLO205, and DLD1 were not authenticated.
Mycoplasma contamination	All cell lines tested negative for mycoplasma.
Commonly misidentified lines (See ICLAC register)	No commonly misidentified cell lines were used.

Animals and other organisms

Policy information about studies involving animals; ARRIVE guidelines recommended for reporting animal research

Laboratory animals	Homozygous Nu/J mice (#002019, Jax), at 6-8 weeks of age were used for xenograft studies. Tumors were initiated 7 days after delivery to allow acclimation of animals. Mice were euthanized before tumors reached a maximum size of 2000mm^3. Male mice were used in this study to recapitulate the majority of CRC cases observed in humans.
Wild animals	No wild animals were used.
Field-collected samples	No field collected samples were used.
Ethics oversight	Mice were maintained at Mount Sinai Animal Research Facilities according to practices prescribed by the NIH and the Mount Sinai Institutional Animal Care and Use Committee (IACUC). The Mount Sinai Animal Research Facilities are fully accredited by the Association for Assessment and Accreditation of Laboratory Animal Care International (AAALAC). All animal experiments were approved by the Mount Sinai Institutional Animal Care and Use Committee (IACUC) (protocol #IACUC-2019-0048 & LA13-00024).

Note that full information on the approval of the study protocol must also be provided in the manuscript.

Human research participants

Policy information about studies involving human research participants

Population characteristics	No population characteristics--de-identified patient surgical samples, exempt.
Recruitment	No recruitment--de-identified patient surgical samples, exempt.
Ethics oversight	All human subjects work was approved by the Mount Sinai Hospital Institutional Review Board (IRB) under Protocol IRB-19-01860. All samples were de-identified by The Mount Sinai Biorepository.

Note that full information on the approval of the study protocol must also be provided in the manuscript.

ChIP-seq

Data deposition

☒ Confirm that both raw and final processed data have been deposited in a public database such as GEO.

☒ Confirm that you have deposited or provided access to graph files (e.g. BED files) for the called peaks.

Data access links *May remain private before publication.*	Raw and processed sequencing data has been deposited at the Gene Omnibus under the accession GSE166254. The token: sfmteagypdghhyd may be used for reviewer access.

Files in database submission

H3K27ac ChIP-seq files and corresponding sample inputs are provided for the following human CRC tumors (suffix T) and adjacent normal human colon epithelium (suffix N): 17627T, 17267N, 17753T, 17753N, 17787T, 18081T, 18081N, 18481T, 18481N, 18607T, 18607N, 18739T, 18739N, 18810T, 18810N, 18946T, 18946N, 19106T, 19106N, 19282T, 19282N, 19342T, 19342N, 19395T, 19395N, 19441T, 19441N, 19442T, 19442N. The following H3K27ac ChIP-seq files are included for human CRC cell lines and xenograft tumors: HT29 cells, HT29 xenograft tumor rep. 1, HT29 xenograft tumor rep. 2, HT29 xenograft tumor rep. 3, HT29 input.

Genome browser session (e.g. UCSC)

All H3K27ac bigwig files for 15 CRC tumors (5 digit de-identified patient ID + T suffix), 15 patient-matched adjacent normal colon epithelium (5 digit de-identified patient ID + N suffix), HT29 cells baseline (HT29_normalized), HT29 xenograft (HT29_xeno1, HT29_xeno2, HT29_xeno3), and HT29 cells treated with TNF-alpha, IFN-gamma, and IL-6 (HT29_x3), are loaded into this DropBox link for accessibly viewing in a genome browser of choice: https://www.dropbox.com/sh/1ihwskrgrnipj6f/AADCqsKP3OAWEFMQezXNl3eya?dl=0

Methodology

Replicates

For primary human tissue, 15 biologically independent tumors and 15 biologically independent patient-matched adjacent normal colon epithelium from 15 independent patients were used. For HT29 xenograft tumors, 3 biologically independent xenograft tumors from 3 independent nude mice were used, all of which were in agreement.

Sequencing depth

All ChIP-seq experiments were single-ended 75 base pair length sequenced and mapped to the hg19 human reference genome. 17627N H3K27ac: 66,782,983 total reads, 38,421,653 uniquely mapped reads. 17627N input: 89,317,166 total reads, 67,555,735 uniquely mapped reads. 17627T H3K27ac: 72,970,439 total reads, 38,558,899 uniquely mapped reads. 17627T input: 82,146,595 total reads, 75,353,415 uniquely mapped reads. 17753N H3K27ac: 54,102,212 total reads, 14,545,653 uniquely mapped reads. 17753N input: 74,248,279 total reads, 56,717,800 uniquely mapped reads. 17753T H3K27ac: 45,071,945 total reads, 36,050,836 uniquely mapped reads. 17753T input: 70,412,704 total reads, 59,632,555 uniquely mapped reads. 17787N H3K27ac: 63,888,545 uniquely mapped reads. 17787N input: 50,391,038 total reads, 45,988,945 uniquely mapped reads. 17787T H3K27ac: 36,050,836 uniquely mapped reads. 17787T input: 59,632,555 uniquely mapped reads. 18081N H3K27ac: 37,421,049 total reads, 26,879,125 uniquely mapped reads. 18081N input: 18081N input: 60,725,408 total reads, 57,025,494 uniquely mapped reads. 18081T H3K27ac: 53,636,709 total reads, 48,714,667 uniquely mapped reads. 18081T input: 52,718,387 uniquely mapped reads. 18481N H3K27ac: 234,024,666 total reads, 101,681,971 uniquely mapped reads. 18481N input: 47,231,094 uniquely mapped reads. 18481T H3K27ac: 35,959,598 uniquely mapped reads. 18481T input: 46,791,007 total reads. 18607N H3K27ac: 71,108,506 total reads, 51,703,983 uniquely mapped reads. 18607N input: 83,443,918 total reads. 18607T H3k27ac: 114,086,103 total reads, 76,709,462 uniquely mapped reads. 18607T input: 97,250,060 total reads, 38,914,390 uniquely mapped reads. 18739N H3K27ac: 34,929,706 total reads, 19,978,111 uniquely mapped reads. 18739N input: 112,064,433 total reads, 36,599,290 uniquely mapped reads. 18739T H3K27ac: 38,708,222 total reads, 21,960,654 uniquely mapped reads. 18739T input: 144,727,580 total reads, 37,606,317 uniquely mapped reads. 18810N H3K27ac: 54,758,251 total reads, 28,021,115 uniquely mapped reads. 18810N input: 54,863,640 total reads, 54,386,517 uniquely mapped reads. 18810T H3K27ac: 45,077,930 total reads, 28,237,472 uniquely mapped reads. 18810T input: 48,409,542 total reads, 45,064,909 uniquely mapped reads. 18946N H3K27ac: 66,269,923 total reads, 33,154,400 uniquely mapped reads. 18946N input: 73,332,644 total reads, 67,268,892 uniquely mapped reads. 18946T H3K27ac: 34,317,015 total reads, 23,959,037 uniquely mapped reads. 18946T input: 111,911,868 total reads, 105,175,823 uniquely mapped reads. 19106N H3K27ac: 76,974,361 total reads, 51,434,158 uniquely mapped reads. 19106N input: 39,816,610 total reads, 37,671,689 uniquely mapped reads. 19106T H3K27ac: 109,718,640 total reads, 51,968,407 uniquely mapped reads. 19106T input: 54,611,731 uniquely mapped reads. 19282N H3K27ac: 75,021,399 total reads, 32,194,116 uniquely mapped reads. 19282N input: 33,916,057 total reads, 30,526,959 uniquely mapped reads. 19282T H3K27ac: 61,651,475 total reads, 41,440,697 uniquely mapped reads. 19282T input: 56,959,861 total reads, 49,870,866 uniquely mapped reads. 19342N H3K27ac: 71,042,523 total reads, 40,509,697 uniquely mapped reads. 19342N input: 29,910,519 total reads, 28,737,350 uniquely mapped reads. 19342T H3K27ac: 55,161,458 total reads, 33,434,438 uniquely mapped reads. 19342T input: 107,075,700 total reads, 104,650,274 uniquely mapped reads. 19395N H3K27ac: 99,397,629 total reads, 44,537,250 uniquely mapped reads. 19395N input: 47,686,547 total reads, 34,891,336 uniquely mapped reads. 19395T H3K27ac: 58,527,712 total reads, 32,705,535 uniquely mapped reads. 19395T input: 171,565,491 total reads, 111,627,571 uniquely mapped reads. 19441N H3K27ac: 55,212,986 total reads, 42,044,084 uniquely mapped reads. 19441N input: 64,641,851 total reads, 61,307,369 uniquely mapped reads. 19441T H3K27ac: 97,023,349 total reads, 46,169,449 uniquely mapped reads. 19941T input: 78,520,201 total reads, 70,972,786 uniquely mapped reads. 19442N H3K27ac: 89,366,838 total reads, 43,352,016 uniquely mapped reads. 19442N input: 103,515,145 total reads, 100,526,505 uniquely mapped reads. 19442T H3K27ac: 104,123,548 total reads, 68,629,671 uniquely mapped reads. 19442T input: 134,341,375 total reads, 131,971,221 uniquely mapped reads. HT29 cells: 65,524,054 uniquely mapped reads. HT29 input: 165,525,214 uniquely mapped reads. HT29 xenograft tumor rep. 1: 32,525,157 uniquely mapped reads. HT29 xenograft tumor rep. 2: 82,915,677 uniquely mapped reads. HT29 xenograft tumor rep. 3: 48,711,330 uniquely mapped reads. HT29 TNF-alpha, IFN-gamma, and IL-6 stimulated cells: 82,167,137 uniquely mapped reads.

Antibodies

H3K27ac ChIP-seq antibodies were purchased from abcam (#ab177178).

Peak calling parameters

Adapter sequences were removed from reads using Cutadapt. Reads were mapped to the hg19 human genome using bowtie. Duplicate reads were removed using samtools. Matching input control was used to call peaks. Peak calling was performed using MACS2. For H3K27ac, a P-value cutoff of 10-10 was used for peak calling. Bigwig tracks were generated using deepTools bamCoverage with RPKM normalization. H3K27ac ChIP-seq tracks were promoter normalized prior to direct comparison within and between patients. Blacklisted regions (Duke_Hg19SignalRepeatArtifactRegions.bed, downloaded from the Broad Institute) were excluded from called peaks using bedtools. Super-enhancer and enhancer calling were performed using Rank Ordering of Super-Enhancers (ROSE) on H3K27ac enrichment using default parameters.

Data quality

H3K27ac ChIP-seq datasets were visually inspected as tracks using the IGV genome browser. All ChIP-seq datasets exhibited exceptionally high signal-to-noise ratios. Using the peak-calling parameters described above, the following number of peaks were called above input using a P-value cut-off of 10^-10. Stitching and subsequent enhancer identification was performed using ROSE. 17627T: 74,355 called peaks stitched to 17,726 enhancers. 17627N: 75,466 called peaks stitched to 21,636 enhancers. 17753T: 59,688 called peaks stitched to 13,726 enhancers. 17753N: 175,589 called peaks stitched to 5,392 enhancers. 17787T: 76,011 called peaks stitched to 20,341 enhancers. 17787N: 66,728 called peaks stitched to 17,567 enhancers. 18081T: 54,343 called peaks stitched

to 15,075 enhancers. 18081N: 52,082 called peaks stitched to 14,110 enhancers. 18481T: 29,640 called peaks stitched to 7,921 enhancers. 18481N: 75,102 called peaks stitched to 24,268 enhancers. 18607T: 47,801 called peaks stitched to 24,704 enhancers. 18607N: 80,274 called peaks stitched to 19,847 enhancers. 18739T: 56,757 called peaks stitched to 15,073 enhancers. 18739N: 34,545 called peaks stitched to 5,692 enhancers. 18946T: 51,652 called peaks stitched to 19,214 enhancers. 18946N: 27,010 called peaks stitched to 15,655 enhancers. 18810T: 72,790 called peaks stitched to 13,804 enhancers. 18810N: 57,932 called peaks stitched to 6,217 enhancers. 19106T: 58,973 called peaks stitched to 16,857 enhancers. 19106N: 61,476 called peaks stitched to 15,331 enhancers. 19282T: 63,909 called peaks stitched to 17,020 enhancers. 19282N: 59,106 called peaks stitched to 15,434 enhancers. 19342T: 53,155 called peaks stitched to 14,616 enhancers. 19342N: 52,206 called peaks stitched to 13,616 enhancers . 19395T: 63,693 called peaks stitched to 18,157 enhancers. 19395N: 47,387 called peaks to 13,483 enhancers. 19441T: 70,089 called peaks stitched to 18,884 enhancers. 19441N: 63,324 called peaks stitched to 16,074 enhancers. 19442T: 62,613 called peaks stitched to 16,760 enhancers. 19442N: 77,621 called peaks stitched to 19,239 enhancers. HT29 cells: 43,055 called peaks stitched to 10.784 enhancers. HT29 xenograft tumor rep. 1: 47,940 called peaks stitched to 13,097 enhancers. HT29 xenograft tumor rep. 2: 61,713 called peaks stitched to 18,869 enhancers. HT29 xenograft tumors rep. 3: 27,347 called peaks stitched to 7,049 enhancers. HT29 TNF-alpha, IFN-gamma, and IL-6 treated cells: 60,741 called peaks stitched to 15,139 enhancers.

Software

The following software was used for the analysis of ChIP-seq data: bedtools v2.21.0, deeptools v3.1.2, macs2 v2.1.0.20140616, Rank Ordering of Super-Enhancers (ROSE) http://younglab.wi.mit.edu/super_enhancer_code.html, The Transcription Factor Affinity Prediction (TRAP) http://trap.molgen.mpg.de/cgi-bin/home.cgi, samtools v1.10, Integrated Genomics Viewer (IGV browser) v.2.9.4, FastQC v0.11.8.
